# An annotated checklist of vascular plants of Cherangani hills, Western Kenya

**DOI:** 10.3897/phytokeys.120.30274

**Published:** 2019-04-18

**Authors:** Yuvenalis Morara Mbuni, Yadong Zhou, Shengwei Wang, Veronicah Mutele Ngumbau, Paul Mutuku Musili, Fredrick Munyao Mutie, Brian Njoroge, Paul Muigai Kirika, Geoffrey Mwachala, Kathambi Vivian, Peninah Cheptoo Rono, Guangwan Hu, Qingfeng Wang

**Affiliations:** 1 Wuhan Botanical Garden, Chinese Academy of Sciences, Wuhan 430074, Hubei, PR China; 2 University of Chinese Academy of Sciences, Beijing 100049, PR China; 3 Sino-Africa Joint Research Center (SAJOREC), Chinese Academy of Sciences, Wuhan 430074, Hubei, PR China; 4 East African Herbarium, National Museums of Kenya, P. O. Box 45166 00100 Nairobi, Kenya

**Keywords:** Checklist, Cherangani hills, vascular plants, diversity, endemism

## Abstract

Cherangani hills, located in Western Kenya, comprises of 12 forest blocks, maintaining great plant diversity. However, little attention to plant diversity studies has been paid to it in the past years. Here, we present a checklist of the vascular plants of this region obtained through intensive field investigations and matching of herbarium specimens. In total, 1296 species, including 17 endemic species are documented, belonging to 130 families and 608 genera. This flora represents 18.50%, 43.83% and 54.17% of the Kenyan species, genera and families, respectively. The habit, habitat and voucher specimens, as well as brief notes on the distribution of each taxon recorded are presented in this checklist. It is the first exhaustive inventory of the terrestrial vascular plants in Cherangani hills which is a significant regional centre for plant diversity.

## Introduction

One of the most critical issues on the global, regional and national agenda is the need to preserve biodiversity for future generations and concurrently strive to understand the indigenous knowledge of resource management practices (Nehal et. al. 2004). The concern for biodiversity preservation by government has highlighted the importance derived from broad and small scale vegetation (Whittaker et al. 1979; Frood and Calder 1987; Lunt 1990). Well managed forests are very useful in biodiversity conservation (Sayer et al. 1995). Conserving plant species of special interest in the tropical forest ecosystems ensures that the vital socio-economic and environmental services (e.g. soil and water conservation and wood and non-wood products) are in constant supply to the local communities living near these forests.

Much of the world’s terrestrial plant diversity occurs in the tropical forests, where they are facing rapid deforestation (FAO 1997). Agricultural farming increased by 200% at the expense of the tropical forests between 1900 and 1990 (UNEP 2009a). Currently, many forests in the tropics are facing a myriad of threats including: unsustainable harvesting practices, illegal encroachment and degradation by the dependent communities (UNDP 2006). Over the last 20 years, the local inhabitants have encroached upon the forest land, converting it into settlement and farmlands (UNEP 2009a). Other factors contributing to the forest loss include change of ecological processes due to climate change, resulting in alteration of biogeochemical cycles and potential species loss (Wang et al. 2001). Moreover, the rapidly increasing human population is the main reason for the large forest loss in East Africa (Lung and Schaab 2009). In addition, ineffective forest policy implementation has also led to the biodiversity loss in Kenya.

The main goal of a checklist is the provision of high-quality baseline data of a given area or forest on all the accepted taxa. The collection of species resource information, namely species inventory, is the primary component of the biodiversity catalogue and the easiest to understand and operate (Heywood et al. 1997), with the nomenclature being the chief focus, as it is fundamental in the communication about plants and the key to biodiversity status. The process involves investigation, classification, ordering, quantification, mapping, analysis and synthesis of species information. Around the world, research on check listing of plants has been done or is on-going in various places (Chong et al. 2009). Although numerous botanists have collected plant specimens from Cherangani hills forest, no comprehensive species studies have been undertaken in all forest blocks (Synott 1979). In Kenya, numerous studies on plant checklists covering different regions have been undertaken on various ecological regions. Some of the major plant checklists include, Bura Tana River (Gachathi et al. 1994), Ol Ari Nyiro Range (Muasya et al. 1994), Mount Elgon (Tweedie 1976), Taita hills (Beentje 1988); (Thijs et al. 2013), Mount Nyiru (Bytebier and Bussmann 2000), Shimba hills (Luke 2005), Kakamega forest (Fischer et al. 2010), Nandi Forests (Girma et al. 2015) and Mount Kenya (Zhou 2017). For Cherangani hills forest, few ad hoc expeditions resulted in several herbarium collections but failed to give the actual floristic listing, composition and distribution of the species (Blackett 1994).

The aim of this paper is to provide the first comprehensive checklist of the vascular species of Cherangani hills. The checklist will provide the basis for further studies of the indigenous species. It will also assist in implementing policies and strategies for the ecological research and conservation of this unique Highland forest ecosystem in Western Kenya.

## Materials and methods

### Study site and current vegetation status

**Figure 1.**
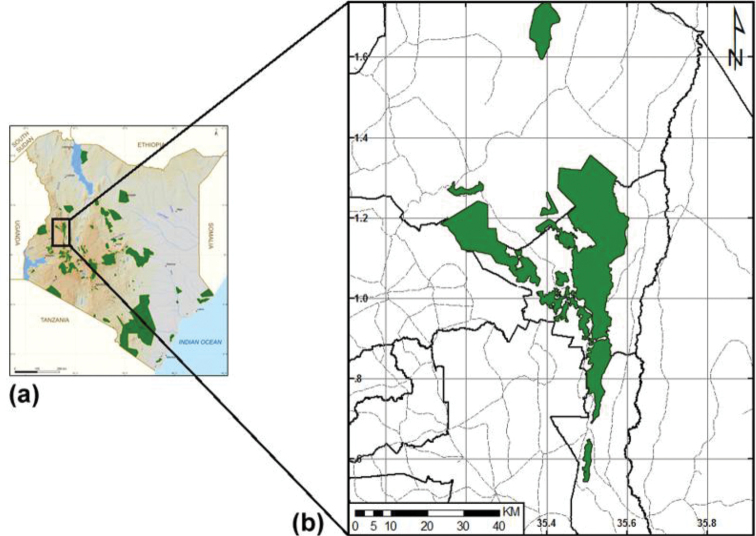
The locality of Cherangani hills. **a** Map of Kenya **b** the forest blocks of Cherangani hills.

Cherangani hills are located in the western side of Kenya within an area defined by 1°16'N, 35°26'E and comprises 12 forest blocks, including Kapolet, Cheboyit, Chemurkoi, Embobut, Kaisungor, Kererr, Kipkunurr, Kiptaberr, Sogotio, Toropket, Kapkanyar and Lelan (UNEP 2009a) (Fig. 1). Within Cherangani hills, relatively undisturbed (nearly primary) forest, as well as disturbed, secondary forest, swamps and riverine forest and natural glades are present. Kapkanyar, Kapolet and Kiptaberr blocks compose the western block Forest Reserves and are comprised of bushland (Pratt et al. 1966), which have undisturbed forest ecology and together total approximately 20,000 ha. They are closed forests as there are no indications of human activity, hence there is a higher plant diversity, which is dominated by *Aningeria*-*Strombosia*-*Drypetes* forest, with a large area of mixed *Podocarpuslatifolius* forest on the higher slopes. According to FAO (2010), all terrestrial ecosystems classified as primary forests are characterised as the most species-rich and diverse. This area is therefore classified as closed forest since it has a tree cover of 40% or more (UNEP et al. 2009b). To the east, forest reserves’ blocks of Lelan, Embobut, Kererr, Kaisungor, Toropket, Chemurkoi, Kipkunurr, Cheboyit, Sogotio and Kapchemutwa are also comprised of woodland and grassland which are separate and have been interfered with by humans and domestic animals. Areas with vegetation cover other than forests, but are not under intensive land use, are defined as woodlands and are mostly used for cattle grazing (Holmgren 2006).

The forests on the south-eastern block along the escarpment crest are fragmented and separated by extensive natural grasslands, scrub and farmland especially in the central part. This area holds *Juniperus*-*Nuxia*-*Podocarpus* forest, with heavily disturbed *Podocarpusfalcatus* forest on the eastern slopes. Generally, most of this area consists of middle-aged secondary forest, but much of it is characterised by very young secondary forest. This area is generally highly degraded and fragmented and the composition of plant communities has been greatly influenced by past commercial logging activities and other anthropogenic disturbances (Mitchell and Schaab 2008). Within the valleys in the upper peak areas, remnants of *Juniperus*-*Maytenusundata*-*Rapanea*-*Hagenia* forest could be found. *Cyatheamanniana* occurs in the stream valleys and there are patches of Bamboo (*Arundinariaalpina*), even though there is no bamboo zone along the Cherangani hills. At higher altitudes, the forest is interspersed with a mixture of heath vegetation and swamps and the latter with *Lobeliaaberdarica*, *Seneciojohnstonii* and *Dendroseneciocheranganiensis*. There are different types of vegetation especially in the east, with little obvious altitudinal zonation and an establishment of plantations of *Cupressuslusitanica*, *Pinuspatula* and a few *Eucalyptus* species.

### Specimen collection and identification

Between July 2015 and May 2018, the botanical team consisting of botanists from the National Museums of Kenya and Sino-Africa Joint Research Center held several explorations in the Cherangani hills. A combination of general walk-over survey method (Filgueiras et al. 1994) and a plotless landscape defined sampling methods for plant specimen collection and sight observation was used to aid the characterisation of the vegetation (Hall and Swaine 1981). Plant specimens bearing flower or fruit were collected and identified. Details of identified specimens were recorded. Specimen details included habit, habitat, distribution and collector details. The specimens were then preserved by pressing.

Voucher specimens were deposited at the East African Herbarium, Nairobi, Kenya (EA) and Herbarium of Wuhan Botanical Gardens, Wuhan, China), labelled with the plant’s scientific name and common names, collection date, GPS coordinates, habitat, collector, identifier and a herbarium specimen number. Standard references were used for plant identification (FTEA 1952–2012; Blundell 1987; Agnew and Agnew 1994; Beentje 1994; Stewart and Campbell 2006; Agnew 2013).

### Checklist

A comprehensive checklist of the vascular plant taxa of the Cherangani hills is enumerated below. Within this checklist, species are organised alphabetically in each family. The families are divided into four groups, namely lycophytes, monilophytes, gymnosperms and angiosperms. Families of lycophytes and monilophytes are organised based on the PPG I system (PPG I 2016), while those of gymnosperms are organised based on Christenhusz et al. (2011) and families of angiosperms are organised based on the APG IV system (APG IV 2016).

## Results and discussion

### Families, genera and species diversity

The current checklist contains a total of 1296 vascular species belonging to 130 families and 608 genera that are indigenous, naturalised or introduced to Cherangani hills. This represents 18.50% of the total number of 7004 Kenyan vascular species obtained, 43.83% of the total number of 1387 Kenyan genera and 54.17% of the total number of 240 Kenyan families (MEWNR and RDA 2015). However, the number of species is still increasing because of the discovery of new records, new naturalised species and the introduction of invasive species.

The top 10 species-rich families are Asteraceae (66 genera/165 species), Fabaceae (39/98), Labiatae (32/68), Orchidaceae (23/65), Rubiaceae (25/65), Poaceae (40/59), Cyperaceae (8/40), Apocynaceae (20/38), Acanthaceae (17/36) and Malvaceae (10/34) (Table 1). Fabaceae, Poaceae and Asteraceae are amongst the three largest families in Cherangani hills and are also the largest families in the world, having 745 genera/19560 species, 707 genera/11337 species and 1620 genera/23600 species, respectively (Stevens 2012) and also the largest families in Kenya, having 98 genera/576 species, 137 genera/565 species and 99 genera/403 species (Zhou et al. 2017a). The 9 most species-rich genera are *Helichrysum* (20, Asteraceae), *Cyperus* (19, Cyperaceae), *Plectranthus* (17, Lamiaceae), *Crotalaria* (14, Fabaceae), *Vernonia* (13, Asteraceae), Euphorbia (13, Euphorbiaceae), Solanum (13, Solanaceae), *Carex* (12, Cyperaceae) and *Justicia* (10, Acanthaceae) (Table 1).

**Table 1.** The 10 largest families and genera of the vascular plants of Cherangani hills.

Family	Species	Genera	Genera	Family	Species
Asteraceae	165	66	* Helichrysum *	Asteraceae	20
Fabaceae	98	39	* Cyperus *	Cyperaceae	19
Lamiaceae	68	32	* Plectranthus *	Lamiaceae	17
Orchidaceae	67	23	* Crotalaria *	Fabaceae	14
Rubiaceae	64	25	* Vernonia *	Asteraceae	13
Poaceae	59	40	* Euphorbia *	Euphorbiaceae	13
Cyperaceae	40	09	* Solanum *	Solanaceae	13
Apocynaceae	36	20	* Carex *	Cyperaceae	12
Acanthaceae	36	17	* Justicia *	Acanthaceae	10
Malvaceae	34	10			

### Growth habit

The growth habit of Cherangani hills signifies that herbaceous species have the highest percentage of 54.39%. Herbs are plants without persistent woody stems. Shrubs are represented with 23.76% and can be defined as self-supporting woody plants branching at or near the ground or with several stems from the base or a single stem less than 2 m. A climber is also defined as a plant that grows upwards by attaching itself to other structures which it uses as support. A tree is a perennial woody plant with a secondary thickening, with a clear main trunk, is dependent on the single trunk and has a height of more than 3 m (Beentje 2016). Trees are also represented with 11.57% of the total species (Table 2). Plant species richness is usually higher in the herbaceous layer than in any other forest stratum. It is often stated that most plant biodiversity in the forest ecosystems is found in the herbaceous layer (Roberts 2004; Whigham 2004), which concludes that herbaceous flora in Cherangani hills has invaded most of the areas and hence has the highest percentage contributing to the high plant diversity.

**Table 2.** Growth habit of the plants of Cherangani Hills.

Habit	No. of species	Percentage
Trees	150	11.57%
Shrubs	308	23.76%
Climbers/Lianas	81	06.25%
Herbs	705	54.39%
Epiphytes	46	3.55%
Parasitic herb	6	0.46%
Total	1296	100%

### Endemic and threatened species

There are 17 endemic plant species in Cherangani hills (Source FTEA), belonging to 5 families and 13 genera and accounting for 1.23% of the total species of which most are from the herb layer (Table 3). The region is not well protected, hence poses great danger to these species, for example, *Dendroseneciocheranganiensis* is classified as a vulnerable species by IUCN (2013). In total, there are 34 threatened plant species (Source FTEA), belonging to 17 families and 28 genera representing 2.62% of the total plants in Cherangani hills (Table 4). Over-exploitation and habitat loss have led to species becoming rare and threatened, for example, *Polysciaskikuyuensis* is highly valued for its excellent timber, hence highly extracted.

**Table 3.** The endemic plants of Cherangani hills.

Family	Endemic species	Growth habit
Acanthaceae	*Justicialeikpiensis* S. Moore	Herb
	*Megalochlamyskenyensis* Vollesen	Herb
Asteraceae	*Conyzaagnewii* Mesfin	Herb
	*Seneciocheranganiensis* Cotton & Blakelock	Herb
	*Seneciohedbergii* C. Jeffrey	Shrub
	*Senecioplantagineoides* C. Jeffrey	Herb
	*Seneciopseudosubsessilis* C. Jeffrey	Herb
	*Seneciosnowdenii* Hutch	Shrub
	*Kleiniascottioides* C. Jeffrey	Herb
	*Bidenselgonensis* (Sherff) Agnew	Herb
	*Carduusschimperi* Sch. Bip.	Herb
	*Dendroseneciocheranganiensis* (Cotton & Blakelock) E.B. Knox	Shrub
	*Emiliatricholepis* C. Jeffrey	Herb
	*Helichrysumbrownei* S. Moore	Shrub
Moraceae	*Dorsteniaafromontana* R.E. Fr.	Herb
Boraginaceae	*Cynoglossumcheranganiense* Verdc.	Herb
Orobanchaceae	*Buchnerascabridula* E.A. Bruce	Herb

**Table 4.** The threatened plants of Cherangani hills.

Family	Species
Apiaceae	*Peucedanumaculeolatum* Engl., *Pimpinellalindblomii* H. Wolff
Araliaceae	*Polysciaskikuyuensis* Summerh.
Asteraceae	*Ethuliavernonioides* (Schweinf.) M.G. Gilbert, *Euryopsbrownei* S. Moore, *Gutenbergiarueppellii* Sch. Bip., *Seneciopseudosubsessilis* C. Jeffrey, *S.rhammatophyllus* Mattf., *Helichrysummeyeri-johannis* Engl., *Guizotiajacksonii* (S. Moore) J. Baagøe.
Balsaminaceae	*Impatienshoehnelii* T.C.E. Fr., *I.meruensis* Gilg, *I.pseudoviola* Gilg, *I.tinctoria* A. Rich.
Boraginaceae	*Cynoglossumcheranganiense* Verdc.
Campanulaceae	*Lobeliaaberdarica* R.E. Fr. & T.C.E. Fr., *L.cheranganiensis* Thulin, *L.deckenii* (Asch.) Hemsl., *L.duriprati* T.C.E. Fr., Wahlenbergiascottii Thulin
Euphorbiaceae	*Euphorbiabrevicornu* Pax
Fabaceae	*Trifoliumcheranganiense* J.B. Gillett
Hypericaceae	*Hypericumkiboense* Oliv.
Leguminosae	*Galegalindblomii* (Harms) J.B. Gillett
Malvaceae	*Abutilonmauritianum* (Jacq.) Medik.
Orchidaceae	*Diaphananthemontana* (Piers) P.J. Cribb & J. Stewart, *Habenariaaltior* Rendle, *Polystachyabella* Summerh.
Poaceae	*Calamagrostishedbergii* Melderis
Ranunculaceae	*Delphiniummacrocentrum* Oliv.
Rosaceae	*Rubusscheffleri* Engl.
Rubiaceae	*Galiumkenyanum* Verdc. *Spermacoceminutiflora* (K. Schum.) Verdc.
Vitaceae	*Cyphostemmacyphopetalum* (Fresen.) Desc. ex Wild & R.B. Drumm.

### Exotic species

A total of 47 species of exotic plants, belonging to 42 genera and 21 families, were realised from Cherangani hills (Table 5). Introduced, cultivated or naturalised species represent 3.62% of the total species of Cherangani hills. The top 4 exotic plant families include: Asteraceae (12 species), Fabaceae (5), Solanaceae (4) and Lamiaceae (3). Out of the 48 plant species introduced, herbs have a higher percentage 63% (30), followed by shrubs 24% (11) and then trees 13% (6). Most of the species have been introduced from South America (14), North America (11), Mexico (10), Central America (5), South Asia (5) and from other regions below 4.

**Table 5.** The exotic plants of Cherangani hills.

Species	Growth Habit	Family	Regions of origin
*Acanthospermumglabratum* (DC.) Wild	Herb	Asteraceae	South America
*Ageratinaadenophora* (Spreng.) R.M. King & H. Rob.	Herb	Asteraceae	Mexico and South America
*Brassicarapa* L.	Herb	Brassicaceae	North America
*Bryophyllumproliferum* Bowie ex Hook.	Herb	Crassulaceae	Madagascar
*Caesalpiniadecapetala* (Roth) Alston	Shrub	Fabaceae	India
*Cestrumaurantiacum* Lindl.	Shrub	Solanaceae	North and South America
*Conyzaboranensis* (S. Moore) Cufod.	Herb	Asteraceae	North America and East Asia
*Cupressuslusitanica* Mill.	Tree	Cupressaceae	North America
*Cuscutacampestris* Yunck.	Climber	Convolvulaceae	North America
*Daturastramonium* L.	Shrub	Solanaceae	Mexico
*Desmodiumadscendens* (Sw.) DC.	Herb	Fabaceae	South America
*Desmodiumuncinatum* (Jacq.) DC.	Shrub	Fabaceae	South Africa
*Erigeronbonariensis* L.	Herb	Asteraceae	South America
*Eucalyptussaligna* Sm.	Tree	Myrtaceae	Australia
*Euphorbiahirta* L.	Herb	Euphorbiaceae	North America
*Galinsogaparviflora* Cav.	Herb	Asteraceae	Central and South America
*Gomphocarpusphysocarpus* E. Mey.	Herb	Apocynaceae	Australia
*Grevillearobusta* A.Cunn. ex R. Br.	Tree	Proteaceae	Australia
*Hyptispectinata* (L.) Poit.	Herb	Lamiaceae	Central America
*Kalanchoeprolifera* (Bowie ex Hook.)	Herb	Crassulaceae	Madagascar
*Lantanacamara* L.	Shrub	Verbenaceae	South America
*Lantanatrifolia* L.	Shrub	Verbenaceae	North America
*Oxalislatifolia* Kunth	Herb	Oxalidaceae	Mexico
*Passifloraedulis* Sims	Climber	Passifloraceae	North America
*Passiflorasubpeltata* Ortega	Climber	Passifloraceae	Mexico and Central America
*Perseaamericana* Mill.	Tree	Lauraceae	Mexico and South America
*Physalisperuviana* L.	Herb	Solanaceae	South America
*Phytolaccaoctandra* L.	Shrub	Phytolaccaceae	Mexico and Central America
*Pinuspatula* Schiede ex Schltdl.	Tree	Pinaceae	Mexico
*Plectranthusbarbatus* Andrews	Shrub	Lamiaceae	South Africa
*Psidiumguajava* L.	Tree	Myrtaceae	Central and South America
*Ricinuscommunis* L.	Herb	Euphorbiaceae	India
*Rubusniveus* Thunb.	Shrub	Rosaceae	South Asia
*Rubusrosifolius* Sm.	Herb	Rosaceae	South Asia
*Rumexacetosella* L.	Herb	Polygonaceae	South Asia
*Salvialeucantha* Cav.	Herb	Lamiaceae	Mexico
*Schkuhriapinnata* (Lam.) Kuntze	Herb	Asteraceae	South America
*Sennaseptemtrionalis* (Viv.) H.S. Irwin & Barneby	Shrub	Fabaceae	Mexico and Central America
*Sennaspectabilis* (DC.) H.S. Irwin & Barneby	Shrub	Fabaceae	Mexico and Central America
*Sigesbeckiaorientalis* L.	Herb	Asteraceae	Australia
*Solanummauritianum* Scop.	Shrub	Solanaceae	South America
*Sonchusoleraceus* (L.) L.	Herb	Asteraceae	Europe and West Asia
*Tagetesminuta* L.	Herb	Asteraceae	South America
*Tithoniadiversifolia* (Hemsl.) A. Gray	Herb	Asteraceae	Mexico & Central America
*Tridaxprocumbens* (L.) L.	Herb	Asteraceae	West Africa
*Xanthiumstrumarium* L.	Herb	Asteraceae	North America

### New records

In the recent years, investigation researchers from Kenya and China have found many new species and several new records in Kenya, including, *Sedumkeniense* (Crassulaceae) (Zhou et al. 2016a), *Zehneriasubcoraicea* (Cucurbitaceae) (Zhou et al. 2016b), *Z.longiflora* (Cucurbitaceae) (Wei et al. 2017), *Cissampeloskeniensis* (Menispermaceae) (Zhou et al. 2017b) and *Adeniaangulosa* (Ngumbau et al. 2017). So far, no new species from this region has been found, although, *Parastrigaalectroides* (Scrophulariaceae), is recorded for the first time in Kenya, particularly in Cherangani hills. An updated description, comparisons with closely related species and revised conservation status for this species has been described.

## Conclusion

Efforts to ensure the conservation of Cherangani forest vegetation habitat should be prioritised with much emphasis on the endangered, rare or endemic species, according to the checklist result. The adopted approach in these studies may help to focus on certain species and the setting of research and conservation priorities. The high proportion of Cherangani endemic species and the geographic rarity component of the flora associated with that of Mount Kenya and Mount Elgon plants, are aspects that makes these habitats interesting. Cherangani hills have not been inventoried exhaustively, including all of its 12 forest blocks. Our study provides the baseline data for further studies on the invasion process and on the patterns of biodiversity. More study approaches may also contribute to the identification of species which are threatened by anthropogenic impacts and therefore require protection. Community-based forest conservation is also suggested to ensure sustainable utilisation of these forests.

## Checklist

The following lists of species include references to the elevation, and where metres are stated, this means metres above sea level. EA means the East African Herbarium, Nairobi, Kenya; HIB means the Herbarium of Wuhan Botanical Garden, Wuhan, China.

### Part 1 Lycophytes

**F1. Lycopodiaceae**

2 Genera, 4 Species

***Huperziadacrydioides* (Baker) Pic. Serm.** Habit: Epiphyte. Habitat: Montane forest, 2000–2500 m. Vouchers: SAJIT 006813 (EA, HIB), FOKP 10913 & 11707 (EA), Mbuni 707 (EA), Evans 811 (EA).

***Huperziaophioglossoides* (Lam.) Rothm.** Habit: Epiphyte. Habitat: Wet forest and montane forest, 1500–2500 m. Vouchers: SAJIT 007111 (EA, HIB), Burtt 1837 & 1838 (EA).

***Huperziasaururus* (Lam.) Travis.** Habit: Epiphyte. Habitat: Moorland, 2550–3500 m. Voucher: Mabberley & McCall 185 (EA).

***Lycopodiumclavatum* L.** Habit: Herb. Habitat: Montane & bamboo zone, 2000–2800 m. Vouchers: SAJIT 006821 (EA, HIB), Mabberley & McCall 42 (EA).

**F2. Selaginellaceae**

1 Genus, 1 Species

***Selaginellaabyssinica* Spring.** Habit: Herb. Habitat: Often moist rock crevices, 1450–2300 m. Voucher: Agnew 5167 (EA).

### Part 2 Monilophytes

**F3. Marattiaceae**

1 Genus, 1 Species

***Marattiafraxinea* Sm.** Habit: Herb. Habitat: Montane forest, 1500–2300 m. Voucher: FOKP 1119 (EA, HIB).

**F4. Hymenophyllaceae**

1 Genus, 1 Species

***Crepidomanesmelanotrichum* (Schltdl.) J.P. Roux** Habit: Epiphyte. Habitat: Wet forest on rocks, 1460–2570 m. Voucher: Thulin & Tidigs 55 (EA).

**F5. Anemiaceae**

1 Genus, 1 Species

***Mohriacaffrorum* (L.) Desv.** Habit: Herb. Habitat: Rock crevices, 1800–2500 m. Voucher: Faden 824 (EA).

**F6. Cyatheaceae**

1 Genus, 1 Species

***Cyatheamanniana* Hook.** Habit: Tree. Habitat: Moist montane forest, in dense shade along streams, 1500–2500 m. Voucher: Mabberley 19 (EA).

**F7. Pteridaceae**

5 Genera, 10 Species

***Adiantumpoiretii* Wikstr.** Habit: Herb. Habitat: Dry montane forest, ericaceous zone, 100–3000 m. Vouchers: FOKP 1709 & 1077 (EA, HIB).

***Cheilanthesfarino*sa (Forssk.) Kaulf.** Habit: Herb. Habitat: Bamboo and moorland zone, 1450–3000 m. Vouchers: SAJIT 004834 (EA, HIB), Mabberley & McCall 240 (EA).

***Cheilanthesbergiana* Schltdl.** Habit: Herb. Habitat: Moist shaded river bank, 1600–2300 m. Voucher: FOKP 1721 (EA, HIB).

***Cheilanthesbergiana* Schltdl.** Habit: Herb. Habitat: Bamboo and moorland zone, 1450–3000 m. Voucher: FOKP 1721 (EA, HIB).

***Cheilanthesquadripinnata* Kuhn** Habit: Herb. Habitat: Moist montane forest, bamboo zone, 1275–2750 m. Vouchers: FOKP 1090 (EA, HIB), Tweedie 3378 (EA).

***Pteriscatoptera* Kunze** Habit: Herb. Habitat: Forest swamp extending to bamboo zones, 1000–3050 m. Voucher: SAJIT 007091 (EA, HIB).

***Pteriscretica* L.** Habit: Herb. Habitat: Dry montane forest, 1500–2700 m. Vouchers: SAJIT 004775 (EA, HIB), FOKP 1003 (EA, HIB), Thulin 56 (EA).

***Pterisdentata* Forssk.** Habit: Herb. Habitat: Upland forest, moist areas, 1000–3000 m. Vouchers: FOKP 1871 & 11367 (EA, HIB), Mbuni 415 (EA).

***Pterisvittata* L.** Habit: Herb. Habitat: Near streams in dense shade, 1250–1600 m. Voucher: Tweedie 2960 (EA).

***Pterispteridioides* (Hook.) Ballard** Habit: Herb. Habitat: Montane forest, 1550–2100 m. Voucher: FOKP 1726 (EA, HIB).

**F8. Aspleniaceae**

1 Genus, 10 Species

***Aspleniumabyssinicum* Fée** Habit: Herb. Habitat: Shade areas and bamboo zone, 1800–3500 m. Vouchers: Evans 812 (EA).

***Aspleniumaethiopicum* (Burm. f.) Bech.** Habit: Epiphyte. Habitat: Moist bushland, 1400–3600 m. Voucher: Part II Botany 221 (EA).

***Aspleniumbugoiense* Hieron.** Habit: Herb. Habitat: Moist montane forest, 2000–2700 m. Voucher: FOKP 1118 (EA, HIB).

***Aspleniumcordatum* (Thunb.) Sw.** Habit: Herb. Habitat: Moist, bush land, woodland, 1530–1830 m. Voucher: SAJIT Z0067 (EA, HIB).

***Aspleniumerectum* Bory ex Willd.** Habit: Herb. Habitat: Moist forest up to bamboo zone, 1300–2800 m. Voucher: FOKP 1122 (EA, HIB).

***Aspleniumlinckii* Kuhn** Habit: Herb. Habitat: Moist forest up to bamboo zone, 1750–2500m. Voucher: FOKP 1896 (EA, HIB).

***Aspleniumloxoscaphoides* Baker** Habit: Herb. Habitat: Dense shade, 2000–2700m. Voucher: SAJIT 007117 (EA, HIB).

***Aspleniummonanthes* L.** Habit: Herb. Habitat: Bamboo zone, 2100–3400 m. Vouchers: FOKP 1078 (EA, HIB), Part II Botany 19 (EA).

***Aspleniumprotensum* Schrad.** Habit: Epiphyte. Habitat: Moist montane forest, 1400–2900 m. Vouchers: SAJIT 004779 (EA, HIB), Part II Botany 18 (EA).

***Aspleniumtheciferum* (Kunth) Mett.** Habit: Epiphyte. Habitat: Moist montane forest, 850–2900 m. Vouchers: FOKP 11642 (EA, HIB), Mbuni 642 (EA), Part II Botany 20 (EA).

**F9. Athyriaceae**

1 Genus, 1 Species

***Depariaboryana* (Willd.) M. Kato** Habit: Herb. Habitat: Moist forest, 1460–2550 m. Voucher: FOKP 1893 (EA, HIB).

**F10. Dryopteridaceae**

4 Genera, 9 Species

***Arachniodeswebbiana* (A. Braun) Schelpe.** Habit: Herb. Habitat: Wet forest, 1550–2600 m. Voucher: SAJIT 007087 (EA, HIB).

***Dryopterispentheri* (Krasser) C. Chr.** Habit: Herb. Habitat: Moist forest, 1550–2500 m. Voucher: Tweedie 2681 (EA).

***Dryopterisschimperiana* (Hochst.) C. Chr.** Habit: Herb. Habitat: Riverine forest, 2750–3220 m. Voucher: Tweedie 2844 (EA).

***Elaphoglossumacrostichoides* (Hook. & Grev.) Schelpe** Habit: Epiphyte. Habitat: Moist montane forest, 1750–2600 m. Voucher: Tweedie 2684 (EA).

***Elaphoglossumdeckenii* (Kuhn) C. Chr** Habit: Epiphyte. Habitat: Moist montane forest and bamboo zone, 2200–3300 m. Voucher: Beentje 3382 (EA).

***Polystichumfuscopaleaceum* Alston** Habit: Herb. Habitat: Moist montane forest, along streams, 2100–2800 m. Voucher: Faden & Evans 810 (EA).

***Polystichumsinense* (Christ) Christ.** Habit: Herb. Habitat: Moist montane forest and Bamboo zone up to moorland, 2430–3500 m. Voucher: FOKP 1004 (EA, HIB).

***Polystichumsetiferum* (Forssk.) Moore ex Woyn.** Habit: Herb. Habitat: Moist montane forest, up to 3500 m. Voucher: FOKP 11382 (EA, HIB).

***Polystichumvolkensii* C. Chr.** Habit: Herb. Habitat: Moist montane forest, up to 3300 m. Voucher: FOKP 1004 (EA, HIB).

**F2. Tectariaceae**

1 Genus, 1 Species

***Tectariagemmifera* (Fée) Alston** Habit: Herb. Habitat: Common in montane forest, 1200–2500 m. Voucher: Tweedie 2702 (EA).

**F12. Polypodiaceae**

4 Genera, 5 Species

***Lepisorusexcavatus* (Bory ex Willd.) Ching** Habit: Epiphyte. Habitat: Montane forest, ericaceous forest, 3150–3350 m. Vouchers: SAJIT 004784 & 007056 (EA, HIB), Thulin & Tidigs 99 & 233 (EA), FOKP 1743 (EA, HIB).

***Drynariavolkensii* Hieron.** Habit: Epiphyte. Habitat: Moist montane forest, 1600–2300 m. Voucher: Tweedie 2799 (EA).

***Loxogrammeabyssinica* (Baker) M.G. Price** Habit: Epiphyte. Habitat: Dry and wet forests, 1000–3000 m. Vouchers: FOKP 11701 (EA, HIB), Mbuni 701 (EA).

***Pleopeltisexcavata* T. Moore** Habit: Epiphyte. Habitat: Moist, riverine forest, 1000–3500 m. Voucher: FOKP 1743 (EA, HIB).

***Pleopeltismacrocarpa* (Bory ex Willd.) Kaulf.** Habit: Epiphyte. Habitat: Moist forest, 1100–3550 m. Vouchers: Verdcourt et al. 2430 (EA), Beentje 3359 (EA).

### Part 3 Gymnosperms

**F13. Podocarpaceae**

2 Genera, 3 Species

***Afrocarpusfalcatus* (Thunb.) C.N. Page.** Habit: Tree. Habitat: Upland forest, 1250–2700 m. Voucher: SAJIT 007061 (EA, HIB).

***Afrocarpusgracilior* (Pilg.) C.N. Page** Habit: Tree. Habitat: Upland forest, 1500–3300 m. Voucher: FOKP 1797 (EA, HIB).

***Podocarpuslatifolius* (Thumb.) R. Br. ex Mirb.** Habit: Tree. Habitat: Wet forest with bamboo, *Hagenia*, *Juniperus* and *Olea* forest, 1500–3300 m. Vouchers: FOKP 11611 & 11770 (EA, HIB), Mbuni 592 & 611 (EA).

**F14. Cupressaceae**

1 Genus, 1 Species

***Juniperusprocera* Hochst. ex Endl.** Habit: Tree. Habitat: Drier upland forest, 1050–2950 m. Vouchers: FOKP 1799 & 11272 (EA, HIB), Mbuni 08 (EA), Malombe & Mlangeni 931 (EA).

### Part 4 Angiosperms

**F15. Piperaceae**

2 Genera, 4 Species

***Peperomiaabyssinica* Miq.** Habit: Herb. Habitat: Upland forest, 1600–2950 m. Vouchers: FOKP 11389 & 11547 (EA, HIB), Mbuni 284 (EA), Hepper & Field 5034 (EA).

***Peperomiaretusa* (L. f.) A. Dietr.** Habit: Epiphyte. Habitat: Wet upland forest, 1500–2400 m. Vouchers: SAJIT 006889 (EA, HIB), FOKP 10989 (EA, HIB).

***Peperomiatetraphylla* (G. Forst.) Hook. & Arn.** Habit: Epiphyte. Habitat: Wet upland forest, 1400–2400 m. Vouchers: SAJIT 004816 & 005065 (EA, HIB), FOKP 1212 (EA, HIB).

***Pipercapense* L. f.** Habit: Herb. Habitat: Wet highland forest, 1200–2700 m. Voucher: FOKP 11416 (EA), Mbuni 152 (EA).

**F16. Annonaceae**

1 Genus, 2 Species

***Uvarialeptocladon* Oliv.** Habit: Shrub. Habitat: Bushland, 0–1900 m. Vouchers: FOKP 11447 (EA), Mbuni 183 (EA).

***Uvariascheffleri* Diels** Habit: Liana. Habitat: Riverine, woodland, bushland, 900–1800 m. Vouchers: FOKP 11469 (EA), Mbuni 205 (EA).

**F17. Monimiaceae**

1 Genus, 1 Species

***Xymalosmonospora* (Harv.) Baill.** Habit: Tree. Habitat: Moist forest, 1400–2700 m. Vouchers: SAJIT 004857 (EA, HIB), FOKP 11362 & 11372 (EA, HIB), Mbuni 108 & 616 (EA), Dale 912 (EA), Friis et al. 2514 (EA).

**F18. Lauraceae**

1 Genus, 1 Species

***Ocoteakenyensis* (Chiov.) Robyns & R. Wilczek** Habit: Tree. Habitat: Moist forest, 1700–2600 m. Voucher: FOKP 1808 (EA, HIB).

**F19. Araceae**

2 Genera, 3 Species

***Arisaemaenneaphyllum* Hochst. ex A. Rich.** Habit: Herb. Habitat: Montane forest edge, 2300–2900 m. Voucher: SAJIT 004859 (EA, HIB).

***Arisaemamildbraedii* Engl.** Habit: Herb. Habitat: Wet montane forest edge, 1400–2620 m. Vouchers: SAJIT 006909 (EA, HIB), FOKP 11009 (EA, HIB).

***Culcasiafalcifolia* Engl.** Habit: Herb. Habitat: Marshy forest edges in high rain forest areas, 1370–2135 m. Vouchers: SAJIT 004753 (EA, HIB), FOKP 737 & 1304 (EA, HIB).

**F20. Potamogetonaceae**

1 Genus, 1 Species

***Potamogetonschweinfurthii* A. Benn**. Habit: Herb. Habitat: Artificial ponds, 1800–2000 m. Voucher: SAJIT 007071 (EA, HIB).

**F21. Dioscoreaceae**

1 Genus, 2 species

***Dioscoreaquartiniana* A. Rich.** Habit: Climber. Habitat: Wooded grassland and bushland, 0–2300 m. Vouchers: SAJIT 005067 (EA, HIB), FOKP 11627 & 11765 (EA, HIB), Mbuni 587 & 627 (EA).

***Dioscoreaschimperiana* Hochst. ex Kunth** Habit: Climber. Habitat: Wooded grassland, 1600–2130 m. Vouchers: Waweru 32 (EA), Nappier 4150 (EA).

**F22. Colchicaceae**

3 Genera, 3 species

***Gloriosasuperba* L.** Habit: Herb. Habitat: Bushland, up to 2500 m. Vouchers: FOKP 11479 & 11694 (EA, HIB), Mbuni 215 & 694 (EA).

***Androcymbiummelanthioides* Willd.** Habit: Shrub. Habitat: Bushland, 1530–2680 m. Voucher: Blake 3090 (EA).

***Wurmbeatenuis* (Hook. f.) Baker** Habit: Herb. Habitat: Grassland, 2130–2750 m. Voucher: Stewart 1002 (EA).

**F23. Smilacaceae**

1 Genus, 1 Species

***Smilaxanceps* Willd** Habit: Climber. Habitat: Wet forest, 10–2800 m. Voucher: FOKP 1071 (EA).

**F24. Orchidaceae**

23 Genera, 65 Species

***Aerangisthomsonii* (Rolfe) Schltr.** Habit: Epiphyte. Habitat: Highland forest, on trunks of trees, above 2300 m. Vouchers: FOKP 11634 (EA), Mbuni 634 (EA).

***Ancistrorhynchusclandestinus* (Lindl.) Schltr.** Habit: Epiphyte. Habitat: On tree branches, 1400–2500 m. Voucher: FOKP 11392 (EA, HIB).

***Angraecumerectum* Summerh.** Habit: Epiphyte. Habitat: Drier forest, 1350–2000 m. Vouchers: FOKP 11303 & 11305 (EA, HIB).

***Angraecumthomsonii* Rolfe** Habit: Epiphyte. Habitat: Shady forest, 1800–2500 m. Voucher: SAJIT 007074 (EA, HIB).

***Anselliaafricana* Lindl.** Habit: Epiphyte. Habitat: Amongst tree roots, 0–2350 m. Vouchers: FOKP 11307 (EA, HIB), Mortimer 210 (EA), Webster 9023 (EA).

***Brachycorythisovata* Lindl.** Habit: Epiphyte. Habitat: Grassland, 2000–2550 m. Vouchers: Napier 1908 (EA), Maas 4691 (EA).

***Bulbophyllumbequaertii* De Wild.** Habit: Epiphyte. Habitat: Shady forest, 1700–2350 m. Voucher: SAJIT 005098 (EA, HIB).

***Bulbophyllumcochleatum* Lindl.** Habit: Epiphyte. Habitat: Shady forest, 1450–2000 m. Voucher: FOKP 11263 (EA, HIB).

***Calanthesylvatica* (Thouars) Lindl.** Habit: Herb. Habitat: Shady forest, 900–2750 m. Voucher: Mabberley 486 (EA).

***Cribbiabrachyceras* (Summerh.) Sengha** Habit: Epiphyte. Habitat: Warm forest near river streams, 1700–2000 m. Voucher: SAJIT 006888 (EA, HIB).

***Cynorkisanacamptoides* Kraenzl**. Habit: Herb. Habitat: Upland moorland and grassland, 2250–3350 m. Vouchers: Dale 3444 (EA), Thulin & Tidigs 190 (EA).

***Cynorkiskassneriana* Kraenzl** Habit: Epiphyte. Habitat: Upland rain forest, on mossy banks, 1600–2580 m. Voucher: Kirk 9418 (EA)

***Diaphanantherohrii* (Rchb. f.) Summerh.** Habit: Epiphyte. Habitat: Dry highland forest, above 2000 m. Voucher: FOKP 11264 (EA, HIB).

***Diaphananthemontana* (Piers) P.J. Cribb & J. Stewart** Habit: Epiphyte. Habitat: Bushland, roots on moss and lichen, 2300–2750 m. Voucher: Stewart 1010 (EA).

***Diaphananthepulchella* Summerh.** Habit: Epiphyte. Habitat: Dry forest, 1500–2330 m. Voucher: FOKP 11260 (EA, HIB).

**Disaaconitoidessubsp.goetzeana (Kraenzl.) H.P. Linder** Habit: Herb. Habitat: Grassland, 2300–2550 m. Voucher: Webster 9036 (EA).

**Disafragranssubsp.deckenii (Rchb. f.) H.P. Linder** Habit: Herb. Habitat: Grassland 2300–3350 m. Vouchers: Thulin & Tidigs 205 (EA), Dale 3251 (EA).

***Disaerubescens* Rendle** Habit: Herb. Habitat: Short upland grass, 1350–2550 m. Vouchers: Lucas 161 (EA), Blake 2128 (EA).

***Disaconcinna* N.E. Br.** Habit: Herb. Habitat: Grassland, 1450–2800 m. Voucher: Napier 1938 (EA).

***Disadecknii* Reichb. f.** Habit: Herb. Habitat: Grassland, 2100–3300 m. Voucher: Dale 3251 (EA).

***Disahircicornis* Rchb. f.** Habit: Herb. Habitat: Wet grassland and swamps, often near streams, 1700–2750 m. Voucher: Dale 3446 (EA).

***Disastairsii* Kraenzl.** Habit: Herb. Habitat: Alpine grassland, Moorland and swamps, 2100–3550 m. Vouchers: Dale 3267 (EA), Thulin & Tidigs 61 (EA).

***Disperiskilimanjarica* Rendle** Habit: Herb. Habitat: Woodland, 2300–3000 m. Voucher: Mabberley & McCall 13 (EA).

***Disperisnemorosa* Rendle** Habit: Herb. Habitat: Mossy areas & leaf litter, 1800–2850 m. Voucher: Dale 34431 (EA).

***Epipactisafricana* Rendle** Habit: Herb. Habitat: River banks, 2330–2830 m. Voucher: Mabberley 221 (EA).

***Eulophiahorsfallii* (Bateman) Summerh.** Habit: Herb. Habitat: Swamps and river edges, 1500–2700 m. Vouchers: Symes 525 (EA), Webster 9040 (EA).

***Eulophialatilabris* Summerh.** Habit: Herb. Habitat: Swampy grassland, 1500–2200 m. Voucher: FOKP 11261 (EA, HIB).

***Eulophiamontis-elgonis* Summerh.** Habit: Herb. Habitat: Swampy grassland, 2000–2350 m. Vouchers: Blake 2016 (EA), Webster 9043 (EA).

***Eulophiastreptopetala* Lindl.** Habit: Herb. Habitat: Grassland, 1350–2330 m. Voucher: SAJIT 007076 (EA, HIB).

***Eulophiataitensis* Pfennig & P.J. Cribb** Habit: Herb. Habitat: Grassland, 1000–2500 m. Voucher: Archer 490 (EA).

***Eulophiapetersii* (Rchb. f.) Rchb. f.** Habit. Herb. Habitat: Grassland, Growing in sandy soil amongst *Sansevieria*, 0–2000 m. Vouchers: Tweedie 68 & 139 (EA).

***Eulophiapyrophila* (Rchb. f.) Summerh.** Habit: Herb. Habitat: Grassland, 1000–2730 m. Voucher: Webster 9045 (EA).

***Habenariaaltior* Rendle** Habit: Herb. Habitat: Grassland, near streams, 1800–3500 m. Voucher: Dale 3447 (EA).

***Habenariacirrhata* (Lindl.) Rchb. f.** Habit: Herb. Habitat: Grassland, Scattered bushes, 300–1050 m. Voucher: Linday 157 (EA).

***Habenariaclavata* (Lindl.) Rchb. f.** Habit: Herb. Habitat: Grassland, 1000–2730 m. Vouchers: Jex–Blake 2127 (EA), Agnew et al. 10580 (EA).

***Habenariamalacophylla* Rchb. f.** Habit: Herb. Habitat: Upland rain, grassland forest, 1350–2700 m. Vouchers: SAJIT 005091 (EA, HIB), FOKP 11645 (EA, HIB), Mbuni 645 (EA), Part II Botany 43 (EA).

***Habenariawalleri* Rchb. f.** Habit: Herb. Habitat: Swampy grassland, 1200–2300 m. Voucher: Webster 9027 (EA).

***Habenariamacruroides* Summerh.** Habit: Herb. Habitat: Open bushland, 1850–2200 m. Voucher: Tweedie 456 (EA).

***Habenariaholubii* Rolfe** Habit: Herb. Habitat: Swamp grassland, 1200–2300 m. Voucher: Blake 2127 (EA).

***Holothrixelgonensis* Summerh.** Habit: Herb. Habitat: Grassland, 3300–3500 m. Voucher: FOKP 11313 (EA, HIB).

***Holothrixbrongniartiana* Rchb. f.** Habit: Herb. Habitat: Grassland, 2300–3200 m. Vouchers: Daicin 3442 (EA), Thulin & Tidigs 150 (EA).

***Holothrixpentadactyla* (Summerh.) Summerh.** Habit: Herb. Habitat: Upland grassland, open rocky ground, 2500–3500 m. Vouchers: Bally 5706 (EA), Townsend 2345(EA), Jex–Blake 5706 (EA), Sharpe 5706 (EA), Stewart 1005 (EA).

***Liparisdeistelii* Schltr.** Habit: Herb. Habitat: Mossy banks, tree trunks, 2000–2700 m. Vouchers: SAJIT 005094 (EA, HIB), Stewart 1099 (EA).

***Polystachyabella* Summerh.** Habit: Epiphyte. Habitat: High up in forest trees, 1800–2300 m. Vouchers: FOKP 11420 (EA), Mbuni 156 (EA).

***Polystachyabennettiana* Rchb. f.** Habit: Epiphyte. Habitat: Drier forest, 1650–2330 m. Vouchers: Webster 1981 & 9030 (EA).

***Polystachyabicarinata* Rendle** Habit: Epiphyte. Habitat: On river streams, 2350–2830 m. Vouchers: SAJIT 004826 & 005099 (EA, HIB), FOKP 11259 & 1012 (EA, HIB), Van Someren 8650 (EA).

***Polystachyacampyloglossa* Rolfe** Habit: Epiphyte. Habitat: Highland forest, 1700–3000 m. Voucher: FOKP 11263 (EA, HIB).

***Polystachyacultriformis* (Thouars) Lindl. ex Spreng**. Habit: Epiphyte. Habitat: River banks, 330–2700 m. Vouchers: SAJIT 005095 (EA, HIB), Webster 9029 (EA).

***Polystachyaeurychila* Summerh.** Habit: Epiphyte. Habitat: On rocks, 1830–2300 m. Voucher: Kirk & Irwin 50 (EA).

***Polystachyaspatella* Kraenzl.** Habit: Epiphyte. Habitat: Highland forest, 2000–2830 m. Vouchers: FOKP 11646 (EA), Mbuni 646 (EA), Friis & Hansen 2510 (EA).

***Polystachyastricta* Rolfe** Habit: Epiphyte. Habitat: Highland forest, 2000–2830 m. Voucher: Webster 9030 (EA).

***Polystachyatenuissima* Kraenzl.** Habit: Epiphyte. Habitat: Highland forests, 1700–2500 m. Vouchers: SAJIT 005096 (EA, HIB), Tweedie 449 (EA).

***Roeperocharisbennettiana* Rchb. f.** Habit: Epiphyte. Habitat: Swamps, 2300–2800 m. Voucher: Dale 3445 (EA).

***Satyriumcarsonii* Rolfe** Habit: Herb. Habitat: Open grassland, 2000–2330 m, Elgeyo open grassland. Vouchers: Napier 2126 (EA), Jex–Blake 2126 (EA).

***Satyriumcoriophoroides* A. Rich.** Habit: Herb. Habitat: Grassland, 1700–2800 m. Vouchers: Symes 95 (EA), Tweedie 1589 (EA).

***Satyriumcrassicaule* Rendle** Habit: Herb. Habitat: Swamps, 1850–3300 m. Voucher: Webster 9034 (EA).

***Satyriumfimbriatum* Summerh.** Habit: Herb. Habitat: Grassland, 2000–3300 m. Vouchers: Stewart 1000 (EA), Dale 3269 (EA), Lucas 163 (EA).

***Satyriumsceptrum* Schltr.** Habit: Herb. Habitat: Grassland, 1700–2800 m. Voucher: Thulin 144 (EA).

***Satyriumschimperi* Hochst. ex A. Rich.** Habit: Herb. Habitat: Grassland, 2300–3200 m. Vouchers: Irwin 426 (EA), Mainwaring 61 (EA).

***Satyriumyunnanense* Rolfe** Habit: Herb. Habitat: Grassland, 1700–2800 m. Voucher: FOKP 11265 (EA, HIB).

***Stolziarepens* (Rolfe) Summerh.** Habit: Epiphyte. Habitat: Riverine forest, 1950–2900 m. Voucher: FOKP 1094 (EA).

***Tridactyleanthomaniaca* (Rchb. f.) Summerh.** Habit: Epiphyte. Habitat: Warm forest, above 1650 m. Voucher: SAJIT 004791 (EA, HIB).

***Tridactylefurcistripes* Summerh.** Habit: Epiphyte. Habitat: highland forest, 2300–3100 m. Vouchers: Bally 2538 (EA), Tweedie 2173 (EA), Beentje 3055 (EA).

***Tridactylenigrescens* Summerh.** Habit: Epiphyte. Habitat: Riverine, Woodland, 1150–2200 m. Voucher: Jackson & Tweedie 3492 (EA).

***Tridactylescottellii* (Rendle) Schltr.** Habit: Epiphyte. Habitat: Rocky grassland and highland forest, 2230–3000 m. Voucher: Beentje 3050 (EA).

**F25. Hypoxidaceae**

I Genus, 5 species

***Hypoxisangustifolia* Lam**. Habit: Herb. Habitat: Grassland in upland, 1300–3000 m. Vouchers: Napier 1907 (EA), Symes 44 & 286 (EA).

***Hypoxiskilimanjarica* Baker** Habit: Herb. Habitat: Grassland, 2900–3250 m. Voucher: Thulin & Tidigs 201 (EA).

***Hypoxisobtusa* Burch. ex Ker Gawl.** Habit: Herb. Habitat: Burnt grassland, 1800–2500 m. Vouchers: Agnew 10484 (EA), Napier 1942 (EA), Symes 287 (EA), Webster 9022 (EA).

***Hypoxisurceolata* Nel** Habit: Herb. Habitat: Grassland, 1300–3000 m. Voucher: Symes 51 (EA).

***Hypoxisgoetzei* Harms** Habit: Herb. Habitat: Burnt grassland, 1830–1920 m. Voucher: Symes 285 (EA).

**F26. Iridaceae**

7 Genera, 14 Species

***Aristeaabyssinica* Pax.** Habit: Herb. Habitat: Wet grassland, 1700–3500 m. Voucher: Tweedie 2985 (EA).

***Aristeaabyssinica* Pax** Habit: Herb. Habitat: Grassland, 2200–2850 m. Voucher: Blake 3108 (EA).

***Aristeacognata* N.E. Br. ex Weim.** Habit: Herb. Habitat: Grassland, 2200–2850 m. Voucher: Symes 155 (EA).

***Dieramacupuliflorum* Klatt** Habit: Herb. Habitat: Grassland, 2400–3500 m. Voucher: Thulin & Tidings 207 (EA).

***Dieramapendulum* (L. f.) Baker** Habit: Herb. Habitat: Grassland, 2400–3500 m. Voucher: Lucas 216 (EA).

***Freesialaxa* (Thunb.) Goldblatt & J.C. Manning** Habit: Herb. Habitat: Rocky grassland, 1700 –3500 m. Voucher: Lucas 188 (EA).

***Gladiolusdalenii* Van Geel** Habit: Herb. Habitat: Damp upland grassland, 1200–3100 m. Voucher: Maas 4690 (EA).

***Gladiolusdichrous* (Bullock) Goldblatt** Habit: Herb. Habitat: Stony highland grassland, 2592–3050 m. Voucher: Mabberley & MacCall 151 (EA).

***Gladiolusschweinfurthii* (Baker) Goldblatt & M.P. de Vos** Habit: Herb. Habitat: Montane grassland, 1830–2290 m. Voucher: Dale 3449 (EA).

***Gladioluswatsonioides* Baker.** Habit: Herb. Habitat: Bamboo and alpine zone, 2400–3550 m. Voucher: FOKP 1812 (EA, HIB).

***Hesperanthapetitiana* (A. Rich.) Baker** Habit: Herb. Habitat: Subalpine and alpine grassland, 1520–3500 m. Voucher: Thulin & Tidings 216 (EA).

***Moreathomsonii* Baker** Habit: Herb. Habitat: Stony grassland, 1400–1920 m. Voucher: Symes 296 (EA).

***Romuleacamerooniana* Baker** Habit: Herb. Habitat: Subalpine, 1520–3500 m. Voucher: Maas 6040 (EA).

***Romuleafischeri* Pax** Habit: Herb. Habitat: Wet upland, 1520–3500 m. Voucher: Thulin & Tidings 110 (EA), Dowson 619 (EA).

**F27. Xanthorrhoeaceae**

2 Genera, 5 species

***Aloecheranganiensis* S. Carter & Brandham** Habit: Shrub. Habitat: Dry scrub, bushed grassland when open, 1200–1900 m. Voucher: Brandham 1727 (EA).

***Aloedawei* A. Berger** Habit: Shrub. Habitat: Rocky bushland, 1830–2120 m. Voucher: FOKP 11326 (EA, HIB).

***Aloependuliflora* Baker** Habit: Shrub. Habitat: Rocky ground, 2100–2500 m. Vouchers: FOKP 11675 (EA), Mbuni 675 (EA).

***Aloesecundiflora* Engl.** Habit: Shrub. Habitat: Sandy soils in drier areas, 900–1800 m. Vouchers: Spoerry 2 & 14 (EA).

***Kniphofiathomsonii* Baker** Habit: Herb. Habitat: Highland grassland, 1900–3550 m. Vouchers: SAJIT 005116 (EA, HIB), Mabberley & MacCall 251 (EA), Lucas 212 (EA).

**F28. Amaryllidaceae**

2 Genera, 2 Species

***Alliumneapolitanum* Cirillo** Habit: Herb. Habitat: Upland grassland, 1500–2500 m. Vouchers: FOKP 11299 (EA, HIB), Mbuni 035 (EA, HIB).

***Scadoxusmultiflorus* (Martyn) Raf.** Habit: Herb. Habitat: Rocky places in forest edges, riverine forest, 5–2250 m. Voucher: SAJIT 007081 (EA, HIB).

**F29. Asparagaceae**

5 Genera, 10 Species

***Anthericumangustifolium* Hochst. ex A. Rich.** Habit: Herb. Habitat: Grassland, 1980–2450 m. Voucher: Blake 1896 (EA).

***Asparagusafricanus* Lam.** Habit: Shrub. Habitat: Bushy wooded, 100–2680 m. Voucher: Symes 281 (EA).

***Asparagusfalcatus*** L. Habit: Climber. Habitat: Upland forest, Woodland, 10–2750 m. Vouchers: FOKP 11699 (EA), Mbuni 699 (EA).

***Asparagusracemosus* Willd.** Habit: Climber. Habitat: Drier bushland 1160–2800 m. Vouchers: FOKP 954, 1018, 11288 & 11699 (EA, HIB), Mbuni 024 & 699 (EA), Webster 9000 (EA), Mus 114 (EA).

***Chlorophytumcameronii* (Baker) Kativu** Habit: Herb. Habitat: Highland grassland, 1675–2450 m. Voucher: Symes 97 (EA).

**Chlorophytumcameroniivar.pterocaulon (Welw. ex Baker) Nordal** Habit: Herb. Habitat: Highland grassland, 1675–2450 m. Vouchers: FOKP 11689 (EA), Mbuni 689 (EA), Symes 36 (EA).

***Chlorophytumaffine* Baker** Habit: Herb. Habitat: Dry bushland, 15–1980 m. Vouchers: Symes 609 (EA), Napier & Tweedie 2119 (EA).

***Dracaenaellenbeckiana* Engl.** Habit: Tree. Habitat: Bushland, 1050–1950 m. Voucher: SAJIT 005050 (EA, HIB).

***Dracaenaafromontana* Mildbr.** Habit: Tree. Habitat: Bamboo forest, 1900–2400 m. Vouchers: FOKP 1070, 11398 & 11522 (EA), Mbuni 134 & 258 (EA), Oteke 97 (EA).

***Ornithogalumgracillimum* R.E. Fr.** Habit: Herb. Habitat: Grassland, 1800–2700 m. Voucher: SAJIT 005076 (EA, HIB).

**F30. Commelinaceae**

5 Genera, 11 Species

***Aneilemarecurvatum* Faden** Habit: Herb. Habitat: Grassland, 700–1300 m. Voucher: Faden 78 (EA).

***Aneilemasebitense* Faden** Habit: Herb. Habitat: Woodland, 1500–1550 m. Vouchers: Faden 803A (EA), Mabberley & McCall 88 & 88A (EA).

***Aneilemazebrinum* Chiov. ex Chiarugi.** Habit: Herb. Habitat: Woodland, 10–1150 m. Voucher: Faden et al. 888 (EA).

***Cyanotisbarbata* D. Don** Habit: Herb. Habitat: Grassland, 1650–3200 m. Vouchers: Stewart 1008 (EA), Symes 617 (EA).

**Commelinaafricanavar.krebsiana (Kunth) C.B. Clarke** Habit: Herb. Habitat: Grassland, 700–2300 m. Voucher: Lye 907 (EA).

***Commelinabenghalensis* L.** Habit: Herb. Habitat: Grassland, bushland, disturbed habitats, 10–2200 m. Vouchers: FOKP 11415 (EA), Mbuni 151 (EA).

***Commelinafoliacea* Chiov.** Habit: Herb. Habitat: Grassland, 350–1550 m. Vouchers: Faden 807 (EA), Mabberley & McCall 88 (EA).

***Commelinaimberbis* Ehrenb. ex Hassk.** Habit: Herb. Habitat: Cultivated ground, disturbed habitat, 1000–1750 m. Voucher: Faden 805 (EA).

***Commelinatriangulispatha* Mildbr.** Habit: Herb. Habitat: Moist roadside banks, 1650–2300 m. Vouchers: Tweedie 4083 (EA), Faden & Evans 487 (EA).

***Floscopaglomerata* (Willd. ex Schult. & Schult. f.) Hassk.** Habit: Herb. Habitat: Standing water in forest, 1650–2200 m. Vouchers: Ivens 1251 (EA), Webster 8995 (EA).

***Murdanniaedulis* (Stokes) Faden** Habit: Herb. Habitat: Grassland, bushland, 900–1300 m. Voucher: Maas 6373 (EA).

**F31. Eriocaulaceae**

1 Genus, 3 Species

***Eriocaulonabyssinicum* Hochst.** Habit: Herb. Habitat: Wet shallow soils, 1650–2730 m. Voucher: SAJIT 005160 (EA, HIB).

***Eriocauloninyangense* Arw.** Habit: Herb. Habitat: Swamps in grassland, around 900 m. Voucher: Townsend 1435 (EA).

***Eriocaulonschimperi* Körn. ex Ruhland** Habit: Herb. Habitat: Heath zones, 2440–3100 m. Vouchers: SAJIT 006822 (EA, HIB), FOKP 945, 1133, 1243 & 10922, (EA, HIB), Fries & Hansen 2533 (EA), Rauh 678 (EA).

**F32. Juncaceae**

2 Genera, 5 Species

***Juncusdregeanus* Kunth** Habit: Herb. Habitat: Upland and alpine forest, 2900–3300 m. Vouchers: FOKP 1148 (EA, HIB), Bogdan 4991 (EA).

***Juncuseffusus* L.** Habit: Herb. Habitat: Highland and moorland forest, 1500–3300 m. Voucher: FOKP 1151 (EA, HIB).

***Juncusoxycarpus* E. Mey. ex Kunth** Habit: Herb. Habitat: Upland streamsides and alpine seepage zones, 1500–2750 m. Vouchers: FOKP 1241 (EA, HIB), Verdcourt 2435 (EA).

***Luzulaabyssinica* Parl.** Habit: Herb. Habitat: High forest streamsides and wet alpine, 2000–3500 m. Voucher: Bogdan 10100 (EA).

***Luzulajohnstonii* Buchenau** Habit: Herb. Habitat: Alpine wet grassland, 2400–3500 m. Vouchers: Thulin & Tidigs 244 (EA), Mabberley 193 (EA).

**F33. Cyperaceae**

9 Genera, 40 Species

**Bulbostylisdensasubsp.afromontana (Lye) R.W. Haines** Habit: Herb. Habitat: Shallow soils, 1500–3000 m. Voucher: Knight 71 (EA).

***Carexbequaertii* De Wild.** Habit: Herb. Habitat: Alpine grassland and montane swamps, 2300–3500 m. Voucher: Thulin & Tidigs 49 (EA).

***Carexchlorosaccus* C.B. Clarke** Habit: Herb. Habitat: Swampy edges, 1500–3300 m. Vouchers: Lye 9125 (EA), Gehrke & Muasya 165 (EA).

***Carexerythrorrhiza* Boeckeler** Habit: Herb. Habitat: Streams, 2500–3500 m. Voucher: Kirk 9887 (EA).

***Carexelgonensis* Nelmes** Habit: Herb. Habitat: Alpine swamps, 1500–3300 m. Voucher: Part II Botany 75 (EA).

***Carexjohnstonii* Boeckeler** Habit: Herb. Habitat: High forest edges, 2200–3300 m. Voucher: Thulin & Tidigs 48 (EA).

***Carexlycurus* K. Schum. ex Engl.** Habit: Herb. Habitat: Montane river-sides, 1950–3300 m. Voucher: Thulin 50 (EA).

***Carexmannii* E.A. Bruce** Habit: Herb. Habitat: Montane forest grades, 2200–3300 m. Voucher: Gehrke & Muasya 167 (EA).

***Carexmonostachya* A. Rich.** Habit: Herb. Habitat: Disturbed bushland, alpine openings, above 2700 m. Voucher: Kirk 58 (EA).

***Carexperegrina* Link** Habit: Herb. Habitat: Common in alpine around, 2300 m. Voucher: Thulin & Tidigs 112 (EA).

***Carexpetitiana* A. Rich.** Habit: Herb. Habitat: Montane forest grades, 2500–3500 m. Voucher: Beentje 212 (EA).

***Carexsteudneri* Boeckeler** Habit: Herb. Habitat: Grassland & woodland edges, 2300–3000 m. Voucher: Bogdan 4972 (EA).

***Carexsimensis* Hochst. ex A. Rich.** Habit: Herb. Habitat: Alpine grassland, 1900–3500 m. Voucher: Thulin 243 (EA).

***Cyperusaterrimus* Hochst. ex Steud.** Habit: Herb. Habitat: Alpine grassland, 1900–3500 m. Voucher: Ivens 1246 (EA).

***Cyperusalternifolius* L.** Habit: Herb. Habitat: Montane forest, 2200–3300 m. Vouchers: Agnew et al. 10458 (EA), Mus 244 (EA), Mbuni 683 (EA).

***Cyperuscyperoides* (L.) Kuntze** Habit: Herb. Habitat: Montane forest, 1600–3500 m. Voucher: Hanson & Thomasson 57 (EA).

***Cyperusdistans* L.** Habit: Herb. Habitat: Temporary water, marshes and ferns up to 2500 m. Voucher: Ivens 1247 (EA).

***Cyperusderreilema* Steud.** Habit: Herb. Habitat: Upland forest and streamsides, 2000–3000 m. Voucher: Ivens 1245 (EA).

***Cyperusdichrostachyus* Hochst. ex A. Rich.** Habit: Herb. Habitat: Highland swamps, 1200–2400 m. Vouchers: SAJIT 004745 (EA, HIB), FOKP 997 & 1152 (EA, HIB).

***Cyperusinvolucratus* Rottb.** Habit: Herb. Habitat: Upland forest, 1800–3000 m. Voucher: Lugard 2793 (EA).

***Cyperusimpubes* Steud.** Habit: Herb. Habitat: Forest shade, 1400–2500 m. Voucher: Maas 4701 (EA).

***Cyperusiria* L.** Habit: Herb. Habitat: Disturbed wet forests, 1000–1500 m. Voucher: Bogdan 5169 (EA).

***Cyperuskerstenii* Boeckeler** Habit: Herb. Habitat: Alpine, Moorland 2500–3500 m. Voucher: Smith et al, 194 (EA).

***Cyperusmollipes* (C.B. Clarke) K. Schum.** Habit: Herb. Habitat: Grassland, 100–2750 m. Voucher: Bogdan 4528 (EA).

***Cyperuscruentus* Rottb.** Habit: Herb. Habitat: Grassland, 500–2000 m. Voucher: Leippert 5116 (EA).

**Cyperusnutansvar.eleusinoides (Kunth) Haines** Habit: Herb. Habitat: streamside, swampy areas, 1000–2200 m. Voucher: Bogdan 5308 (EA).

***Cyperusplatycaulis* Baker** Habit: Herb. Habitat: Wet areas, 1000–3000 m. Vouchers: FOKP 980 & 1134 (EA, HIB), Smith, Beentje & Muasya 208 (EA), Botany 2 Exp. 74 (EA).

***Cyperusrotundus* L.** Habit: Herb. Habitat: Grassland, Up to 2000 m. Vouchers: Mbuni 014 (EA), FOKP 11278 (EA).

***Cyperusschimperianus* Steud.** Habit: Herb. Habitat: River-side swamps, Sandy, stony river banks, grassland, 450–1600 m. Voucher: Thomas 2117 (EA).

***Cyperussquarrosus* L.** Habit: Herb. Habitat: Wet pool edges, up to 2000 m. Voucher: Leippert 5041 (EA).

***Fimbristylisdichotoma* (L.) Vahl** Habit: Herb. Habitat: Grassland, up to 2000 m. Voucher: Bogdan 4960 (EA).

**Fuirenastrictasubsp.chlorocarpa (Ridl.) Lye** Habit: Herb. Habitat: Wet grassland, 500–2800 m. Voucher: Bogdan 4992 (EA).

***Isolepiscostata* Hochst. ex A. Rich.** Habit: Herb. Habitat: Alpine, Sedge in dense growth, 1700–3500 m. Voucher: Bogdan 4990 (EA).

***Isolepisfluitans* (L.) R. Br.** Habit: Herb. Habitat: Montane water, 1300–3500 m. Voucher: Thulin & Tidigs 97 (EA).

***Kyllingaalba* Nees** Habit: Herb. Habitat: Dry bushland, 500–2500 m. Voucher: Napper & Tweedie 2124 (EA).

***Kyllingabrevifolia* Rottb** Habit: Herb. Habitat: Streamside, 1600–3000 m. Vouchers: FOKP 11731 (EA), Mbuni 731 (EA).

***Kyllingaodorata* Vahl** Habit: Herb. Habitat: Streamside, 1300–3300 m. Vouchers: Smith et al. 196 (EA), Bogdan 5002 (EA).

***Pycreuselegantulus* (Steud.) C.B. Clarke** Habit: Herb. Habitat: Grassland, 1000–2200 m. Vouchers: FOKP 990 & 1130 (EA, HIB).

***Kyllingapulchella* Kunth** Habit: Herb. Habitat: Woodland edge, 1500–2600 m. Voucher: Smith et al. 190 (EA).

***Schoenoplectuscorymbosus* (Roth ex Roem. & Schult.) J. Raynal** Habit: Herb. Habitat: Grassland, 1000–2500 m. Vouchers: FOKP 984, 1141 (EA, HIB) Bogdan 5313 (EA), Muasya 1284 (EA).

**F34. Poaceae**

40 Genera, 59 Species

***Agrostiskilimandscharica* Mez.** Habit: Herb. Habitat: Bamboo and upland, 2000–3500 m. Voucher: Bogdan 4974 (EA).

***Agrostisproducta* Pilg.** Habit: Herb. Habitat: Upland grassland & moorland 2500–3500 m, Vouchers: Bogdan 4977 (EA), Thulin et al. 256 (EA).

***Agrostisquinqueseta* (Steud.) Hochst.** Habit: Herb. Habitat: Moorland & alpine, 3000–3500 m. Voucher: Bogdan 4973 (EA).

***Airacaryophyllea* L**. Habit: Herb. Habitat: Grassland and moorland above 2000 m. Vouchers: Bogdan 4989 (EA), Braun 582 (EA).

***Andropogonamethystinus* Steud.** Habit: Herb. Habitat: Alpine grassland, 2000–3550 m. Vouchers: Thulin & Tidigs 225 (EA), Braun 581 (EA).

***Andropogonchinensis* (Nees) Merr.** Habit: Herb. Habitat: Savannah and bushland, 0–1800 m. Voucher: Bogdan 3829 (EA).

***Andropogonschirensis* Hochst.** Habit: Herb. Habitat: Grassland, 2000–3500 m. Voucher: Strange 82 (EA).

***Anthephoranigritana* Stapf & C.E. Hubb.** Habit: Herb. Habitat: Rocky bushland, 1200–1700 m. Vouchers: Bogdan 3407 & 5633 (EA).

***Anthoxanthumnivale* K. Schum.** Habit: Herb. Habitat: Upland grassland & moorland, above 2500 m. Vouchers: Bogdan 4971 & Bogdan 4997 (EA).

***Aristidaadscensionis* L.** Habit: Herb. Habitat: Bushland, 1500–2500 m. Voucher: Bogdan 5632 (EA).

***Aristidahordeacea* Kunth** Habit: Herb. Habitat: Bushland, 400–1600 m. Voucher: Bogdan 3414 (EA).

***Bothriochloaradicans* (Lehm.) A. Camus** Habit: Herb. Habitat: Deciduous bushland, 0–2000 m. Voucher: Bogdan 3413 (EA).

***Brachiariascalaris* Pilg.** Habit: Herb. Habitat: Upland weedy places, 900–2400 m. Vouchers: Bogdan 5306 (EA), Baner 481 (EA).

***Brachypodiumflexum* Nees** Habit: Herb. Habitat: Upland forest, 2000–3000 m. Voucher: Bogdan 4870 (EA).

***Cymbopogongiganteus* Chiov.** Habit: Herb. Habitat: Bushland, 0–2300 m. Voucher: Bogdan 3854 (EA).

***Digitariagayana* (Kunth) A. Chev.** Habit: Herb. Habitat: Disturbed place, farmland, 1200–2000 m. Voucher: Bogdan 4337 (EA).

***Digitariavelutina* (Forssk.) P. Beauv.** Habit: Herb. Habitat: Disturbed places, 0–2300 m. Voucher: Baner 480 (EA).

***Dinebraretroflexa* (Vahl) Panz.** Habit: Herb. Habitat: Black clay soils, 500–2000 m. Voucher: Pratt 5716 (EA).

***Ehrhartaerecta* Lam.** Habit: Herb. Habitat: Forest clearing, 1500–2700 m. Voucher: Bogdan 5305 (EA).

***Eragrostiscylindriflora* Hochst**. Habit: Herb. Habitat: Dry bushland, 1200–2000 m. Voucher: Bogdan 3422 (EA).

***Eragrostispatens* Oliv.** Habit: Herb. Habitat: Dry land area, 400–2000 m. Voucher: Bogdan 3423 (EA).

***Eragrostistremula* Hochst. ex Steud.** Habit: Herb. Habitat: Grassland, 0–1600 m. Voucher: Bogdan 4492 (EA).

***Exothecaabyssinica* (Hochst. ex A. Rich.) Andersson** Habit: Herb. Habitat: Upland and montane grassland, 2000–3550 m. Voucher: Strange 85 (EA).

***Festucaabyssinica* A. Rich.** Habit: Herb. Habitat: Highland grassland and moorland, 2000–3000 m. Voucher: Heady 1415 (EA).

***Hyparrheniapilgeriana* C.E. Hubb.** Habit: Herb. Habitat: Swamps, 1000–2700 m. Voucher: Bogdan 4513 (EA).

***Hyparrheniavariabilis* Stapf.** Habit: Herb. Habitat: Deciduous bushland, wooded grassland, 600–2200 m. Voucher: Bogdan 3635 (EA).

***Koeleriacapensis* Nees** Habit: Herb. Habitat: Upland Grassland and moorland, 2000–3550 m. Voucher: Thulin & Tidigs 249 (EA).

***Leersiahexandra* Sw.** Habit: Herb. Habitat: Shallow water and swamps, up to 2000 m. Voucher: Ivens 1283 (EA).

***Leptochloarupestris* C.E. Hubb.** Habit: Herb. Habitat: Deciduous bushland, rocky places, 1600–1900 m. Voucher: Bogdan 3844 (EA).

***Loudetiaflavida* (Stapf) C.E. Hubb.** Habit: Herb. Habitat: Dry bushland, 300–1600 m. Voucher: Deg 32 (EA).

***Loudetiakagerensis* (K. Schum.) C.E. Hubb.** Habit: Herb. Habitat: Stony soil, 1200–2700 m. Voucher: Kuchar 8653 (EA).

***Microchloaindica* (L. f.) P. Beauv.** Habit: Herb. Habitat: Low altitude, shallow soil, 800–1600 m. Voucher: Bogdan 5171 (EA).

***Melinisrepens* (Willd.) Zizka** Habit: Herb. Habitat: Grassland, 0–2500 m. Voucher: FOKP 1034 (EA).

***Melinisnerviglumis* (Franch.) Zizka** Habit: Herb. Habitat: Upland grassland and open woodland, 500–2000 m. Voucher: Bogdan 3426 (EA).

***Oplismenuscompositus* (L.) P. Beauv.** Habit: Herb. Habitat: Evergreen grassland, 0–2400 m. Voucher: Bogdan 4952 (EA).

***Oplismenushirtellus* (*L.*) P. Beauv.** Habit: Herb. Habitat: Forest shade, 0–2500 m. Voucher: Bogdan 4952 (EA).

***Oplismenusundulatifolius* (Ard.) Roem. & Schult.** Habit: Herb. Habitat: Forest shade, 1400–2500 m. Voucher: Mabberley & McCall 60 (EA).

***Oropetiumminimum* (Hochst.) Pilg.** Habit: Herb. Habitat: Open deciduous, dry grassland, 780–2600 m. Voucher: Bogdan 4948 (EA).

***Oropetiumthomaeum* (L. f.) Trin.** Habit: Herb. Habitat: Deciduous grassland, 500–1100 m. Voucher: Bogdan 4949 (EA).

***Panicumatrosanguineum* Hochst. ex A. Rich.** Habit: Herb. Habitat: Dry bushland, 100–2200 m. Voucher: Baner 482 (EA).

***Panicumpusillum* Hook. f.** Habit: Herb. Habitat: Grassland, moorland, Streams, 1300–3300 m. Voucher: Bogdan 5299 (EA).

***Panicumhochstetteri* Steud.** Habit: Herb. Habitat: Drier in forest shade, 1500–3000 m. Voucher: Bogdan 3835 (EA).

***Panicumhymeniochilum* Nees.** Habit: Herb. Habitat: Wet places, 1000–3000 m. Vouchers: Thulin & Tidigs 180, Thulin et al. 161 (EA).

***Pennisetummacrourum* Trin.** Habit: Herb. Habitat: River banks & Streams, 700–2300 m. Voucher: Bogdan 3637 (EA).

***Pennisetumsphacelatum* (Nees) T. Durand & Schinz.** Habit: Herb. Habitat: Bushland, 1500–3200 m. Voucher: Thulin & Tidigs 277 (EA).

***Pennisetumsquamulatum* Fresen.** Habit: Herb. Habitat: Deciduous bushland, 300–1600 m. Voucher: Bogdan 3833 (EA).

***Pogonarthriasquarrosa* (Roem. & Schult.) Pilg.** Habit: Herb. Habitat: Open moorland, 500–2000 m. Voucher: Bogdan 3447 (EA).

***Pogonarthriasquarrosa* (Roem. & Schult.) Pilg.** Habit: Herb. Habitat: Sandy grassland and open woodland, 500–1870 m. Voucher: Bogdan 3447 (EA).

***Polypogonschimperianus* (Hochst. ex Steud.) Cope** Habit: Herb. Habitat: Moorland & alpine, 3000–3500 m. Voucher: Ivens 1244 (EA).

***Sacciolepisafricana* C.E. Hubb. & Snowden.** Habit: Herb. Habitat: Shallow water up to 1600 m. Voucher: Knight 5309 (EA).

***Sacciolepisindica* (L.) Chase** Habit: Herb. Habitat: streamside marshes, 1000–2200 m. Voucher: Bogdan 5309 (EA).

***Schmidtiapappophoroides* Steud. ex J.A. Schmidt** Habit: Herb. Habitat: Bushland, 200–2000 m. Voucher: Bogdan 3845 (EA).

***Setariaorthosticha* K. Schum. ex R.A.W. Herrm.** Habit: Herb. Habitat: Evergreen grassland, 1000–2300 m. Voucher: Bogdan 3851 (EA).

***Sporoboluspanicoides* A. Rich.** Habit: Herb. Habitat: Deciduous bushland, 1100–2000 m. Voucher: Bogdan 3884 (EA).

***Stipakeniensis* (Pilg.) Freitag** Habit: Herb. Habitat: Upland forest shade and road sides, 2000–2700 m. Voucher: Bogdan 500 (EA).

***Streblochaetelongiarista* (A. Rich.) Pilg.** Habit: Herb. Habitat: forest shade & bamboo clearing, 1500–3000 m. Voucher: Thorold 2758 (EA).

***Urochloaoligotricha* (Fig. & De Not.) Henrard.** Habit: Herb. Habitat: Wooded grassland, 1300–2000 m. Voucher: Bogdan 3409 (EA).

***Vulpiabromoides* (L.) Gray** Habit: Herb. Habitat: Rocky soils, 2500–3500 m. Vouchers: Verdcourt 2439A (EA), Taylor et al. 2439 (EA).

***Yushaniaalpina* (K. Schum.) W.C. Lin** Habit: Herb. Habitat: Montane forest 3000–3300 m. Voucher: Davidse 9224 (EA).

**F35. Papaveraceae**

1 Genus, 1 Species

***Corydalismildbraedii* Fedde** Habit: Herb. Habitat: Grassland, 2200–3500 m. Voucher: Thulin et al. 235 (EA).

**F36. Menispermaceae**

2 Genera, 2 Species

***Cissampelospareira* L.** Habit: Herb. Habitat: Woodland, 2–2600 m. Vouchers: FOKP 11289 & 11604 (EA, HIB), Mbuni 593 & 604 (EA).

***Stephaniaabyssinica* (Quart.-Dill. & A. Rich.) Walp.** Habit: Liana. Habitat: bushland, 1950–2600 m. Vouchers: SAJIT 005058 (EA, HIB), FOKP 934 (EA, HIB).

**F37. Berberidaceae**

1 Genus, 1 Species

***Berberisholstii* Engl.** Habit: Shrub. Habitat: Upland bushland, 2150–3000 m. Voucher: SAJIT 007059 (EA, HIB).

**F38. Ranunculaceae**

6 Genera, 9 Species

***Anemonethomsonii* Oliv.** Habit: Herb. Habitat: Grassland, 2500–3500 m. Vouchers: Bogdan 4994 (EA), Knox 3392 (EA).

***Clematisbrachiata* Thunb.** Habit: Climber. Habitat: Wooded grassland, 720–3150 m. Vouchers: FOKP 11277 (EA, HIB), Webster 8708 (EA), Lind 5094 (EA), Ivens 1254 (EA).

***Clematissimensis* Fresen.** Habit: Climber. Habitat: Grassland 1600–3250 m. Vouchers: FOKP 993, 1022 & 11512 (EA, HIB), Mbuni 248 (EA), Lind 2851 (EA), Knox 3857 (EA), Mabberley et al. 227 (EA).

***Clematisvillosa* DC.** Habit: Herb. Habitat: Grassland, 1600–3250 m Vouchers: Symes 42 (EA), Webster 8710 (EA).

***Delphiniummacrocentrum* Oliv.** Habit: Herb. Habitat: grassland, 1650–3500 m. Vouchers: FOKP 11387 (EA, HIB), Lind 5099 (EA), Mabberley 235 (EA).

***Ranunculusmultifidus* Forssk.** Habit: Herb. Habitat: streamside, 1350–3450 m. Vouchers: FOKP 1137, 1207 & 11360 (EA, HIB), Mbuni 096 (EA), Mabberley & McCall 213 (EA), Thulin et al. 78 (EA).

***Ranunculusoreophytus* Delile** Habit: Herb. Habitat: streamside, 2550–3500 m. Vouchers: Knox 3383 (EA), Trelawny 4385 (EA).

***Ranunculusvolkensii* Engl.** Habit: Herb. Habitat: Grassland, 2550–3400 m. Vouchers: Thulin & Tidigs 194 (EA), Knox 3382 (EA).

***Thalictrumrhynchocarpum* Dillon & A. Rich** Habit: Herb. Habitat: Upland forest, 1550–3275 m. Vouchers: SAJIT 005057 (EA, HIB), FOKP 11533 (EA), Mbuni 269 (EA), Mabberley & McCall 188 (EA).

**F39. Proteaceae**

2 Genera, 6 Species

***Faureaarborea* Engl.** Habit: Tree. Habitat: Wooded grassland, 1600–2400 m. Voucher: Thulin & Tidings 181 (EA).

***Faurearochetiana* (A. Rich.) Chiov. ex Pic. Serm.** Habit: Tree. Habitat: Wooded grassland, 1600–2400 m. Vouchers: FOKP 11325 (EA, HIB), Birch 61 & 178 (EA).

***Faureasaligna* Harv.** Habit: Tree. Habitat: Wooded grassland, 1600–2400 m. Vouchers: SAJIT 004766 (EA, HIB), FOKP 1092 & 11268 (EA, HIB), Mbuni 004 (EA), Trapnell 2301 (EA), Botany Part II 61 (EA), Buch 178 (EA).

**Proteacaffrasubsp.kilimandscharica (Engl.) Chisumpa & Brummitt** Habit: Shrub. Habitat: Moorland, 2500–3500 m. Voucher: Dale 862 (EA).

***Proteagaguedi* J.F. Gmel.** Habit: Tree. Habitat: Grassland bushland, 1500–2900 m. Vouchers: SAJIT 005146 (EA, HIB), FOKP 11275 (EA), Mbuni 014 (EA), Thulin & Tidigs 73 (EA), Napper 1504 (EA).

***Proteamadiensis* Oliv.** Habit: Shrub. Habitat: Wooded grassland, 1650–1900 m. Voucher: Buch 161 (EA).

**F40. Gunneraceae**

1 Genus, 1 Species

***Gunneraperpensa* L.** Habit: Herb. Habitat: River-side marshes, 2300–3370 m. Voucher: Tweedie 3014 (EA).

**F41. Crassulaceae**

5 Genera, 16 Species

***Bryophyllumpinnatum* (Lam.) Oken** Habit: Herb. Habitat: Grassland, 1000–2100 m. Voucher: Symes 2799 (EA).

***Crassulaalata* (Viv.) A. Berger** Habit: Herb. Habitat: Grassland, 1350–2700 m. Voucher: Maas 6348 (EA).

***Crassulaalba* Forssk.** Habit: Herb. Habitat: Grassland, 2439–3048 m. Vouchers: SAJIT 005115 & 004811 (EA, HIB), Dale 3271 (EA).

***Crassulaalsinoides* (Hook. f.) Engl.** Habit: Herb. Habitat: Wet rocky, 1475–3500 m. Vouchers: SAJIT 006816 (EA, HIB), FOKP 10916 & 11530 (EA, HIB), Mbuni 266 (EA), Mabberley 572 (EA).

***Crassulagranvikii* Mildbr.** Habit: Herb. Habitat: Alpine 1650–3500 m. Vouchers: FOKP 1214 & 11733 (EA, HIB), Mbuni 112 & 733 (EA), Thulin 204 (EA).

***Crassulaschimperi* Fisch. & C.A. Mey.** Habit: Herb. Habitat: Upland grassland, 1050–3550 m. Voucher: SAJIT 004833 (EA).

***Crassulavaginata* Eckl. & Zeyh.** Habit: Herb. Habitat: Upland grassland, 2000–3500 m. Voucher: Lucas 214 (EA).

***Kalanchoecitrina* Schweinf.** Habit: Herb. Habitat: Bushland, 1150–2100 m. Voucher: Tweedie 1773 (EA).

***Kalanchoecrenata* (Andrews) Haw.** Habit: Herb. Habitat: Grassland, 1650–2300 m. Vouchers: SAJIT 006894 (EA, HIB), Symes 279 (EA).

***Kalanchoedensiflora* Rolfe** Habit: Herb; Habitat: Montane grassland, 1500–3000 m. Vouchers: SAJIT 006793 (EA, HIB), FOKP 11380 (EA), Mbuni 129 (EA), Thulin & Tidigs 85 (EA), Rauh 683 (EA).

***Kalanchoeglaucescens* Britten** Habit: Herb. Habitat: Bushland, 900–2200 m. Vouchers: FOKP 1279 (EA, HIB), Mortimer 207 (EA).

***Kalanchoelanceolata* (Forssk.) Pers.** Habit: Herb. Habitat: Grassland, Dry areas, 950–2100 m. Vouchers: SAJIT 006899 (EA, HIB), Tweedie 1774 (EA).

***Kalanchoeprittwitzii* Engl.** Habit: Herb. Habitat: Bushland, 1650–2300 m. Voucher: SAJIT 006845 (EA, HIB).

***Sedummeyeri-johannis* Engl.** Habit: Herb. Habitat: Heartland, 2100–3150 m. Vouchers: SAJIT 006824 (EA, HIB), FOKP 10924 (EA, HIB).

***Sedumruwenzoriense* Baker f.** Habit: Herb. Habitat: Upland heartland, 2800–3500 m. Vouchers: Mabberley & McCall 230 (EA), Symes 649 (EA).

***Umbilicus botryoides* Hochst. ex A. Rich.** Habit: Herb. Habitat: Montane forest, 2250–3500 m. Voucher: SAJIT 004850 (EA).

**F42. Haloragaceae**

1 Genus, 1 Species

***Laurembergiatetrandra* (Schott) Kanitz** Habit: Herb. Habitat: Wet grassland at streamside, 2000–2350 m. Vouchers: SAJIT 006823 (EA, HIB), FOKP 10923 (EA, HIB).

**F43. Vitaceae**

4 Genera, 14 Species

***Ampelocissusafricana* (Lour.) Merr.** Habit: Liana. Habitat: Riverine, woodland, 1450–1800 m. Voucher: Napier 2000 (EA).

***Cissuspetiolata* Hook. f.** Habit: Liana. Habitat: Riverine forest, 1050–1800 m. Voucher: Balley 12355 (EA).

***Cissusrotundifolia* Vahl** Habit: Shrub. Habitat: *Acacia–Commiphora* bushland, 0–2100 m. Voucher: SAJIT Z0041 (EA, HIB).

***Cissusruspolii* Gilg** Habit: Climber. Habitat: Bushland, 1300–1620 m. Voucher: Meyerhoff 102 (EA).

***Cyphostemmabambuseti* (Gilg & M. Brandt) Desc. ex Wild & R.B. Drumm.** Habit: Climber. Habitat: Dry upland forest, 1450–2400 m. Voucher: SAJIT 004750 (EA, HIB).

***Cyphostemmajunceum* (Baker) Desc. ex Wild & R.B. Drumm.** Habit: Climber. Habitat: Grassland 1650–2280 m. Voucher: Symes 292 (EA).

***Cyphostemmacyphopetalum* (Fresen.) Desc. ex Wild & R.B. Drumm.** Habit: Climber. Habitat: Bushland, 470–2450 m. Vouchers: FOKP 11752 (EA), Mbuni 752 (EA), Symes 334 (EA).

***Cyphostemmadysocarpum* (Gilg & M. Brandt) Desc.** Habit: Climber. Habitat: *Acacia–Commiphora* bushland, 1000–1600 m. Voucher: Lindsay 129 (EA).

***Cyphostemmaheterotrichum* (Gilg & R.E. Fr.) Desc. ex Wild & R.B. Drumm.** Habit: Climber. Habitat: Grassland, 1600–2300 m. Vouchers: Napier 2028 (EA), Symes 103 (EA).

***Cyphostemmakilimandscharicum* (Gilg) Desc. ex Wild & R.B. Drumm.** Habit: Climber. Habitat: Montane rain forest, 1600–3040 m. Voucher: SAJIT 005070 (EA, HIB).

***Cyphostemmamaranguense* (Gilg) Desc.** Habit: Climber. Habitat: Dry upland forest, 1600–3040 m. Voucher: FOKP 11276 (EA, HIB).

***Cyphostemmapseudosesquipedale* Verdc.** Habit: Herb. Habitat: Grassland up to 1372 m. Voucher: Agnew et al. 10230 (EA).

***Cyphostemma Serpens* (Hochst. ex A. Rich.) Desc.** Habit: Climber. Habitat: Bushed grassland, 1200–2550 m. Vouchers: SAJIT 006876 (EA, HIB), FOKP 11281 (EA, HIB).

***Rhoicissustridentata* (L. f.) Wild & R.B. Drumm.** Habit: Shrub. Habitat: Wooded, 60–2700 m. Vouchers: SAJIT 004846 (EA, HIB), FOKP 1108, 1262, 11280 & 11451 (EA, HIB), Mbuni 187 & 679 (EA).

**F44. Zygophyllaceae**

1 Genus, 1 Species

***Tribulusterrestris* L.** Habit: Herb. Habitat: Waste places and trodden earth, 10–2300 m. Vouchers: FOKP 11488 (EA), Mbuni 224 (EA).

**F45. Fabaceae**

39 Genera, 98 Species

***Vachelliaabyssinica* Benth.** Habit: Tree. Habitat: Wooded grassland, 1450–3300 m. Vouchers: FOKP 11269 (EA), Mbuni 005 (EA).

***Senegaliabrevispica* Harms** Habit: Tree. Habitat: Dry bushland, thickets, 170–1830 m. Vouchers: SAJIT Z0058 (EA, HIB), Napier 2037 (EA).

***Vachelliahockii* De Wild.** Habit: Tree. Habitat: Wooded grassland, 750–2250 m. Vouchers: FOKP 1266 (EA, HIB), Mbuni 654 (EA).

***Vachellialahai* Benth.** Habit: Tree. Habitat: Upland forest, dense woodland, 1500–2700 m. Vouchers: Mbuni 005 (EA), Napier 1952 & 2014 (EA), Padwa 56 (EA), Brown 577 (EA).

***Mimosaoerfota* (Forssk.) Schweinf.** Habit: Tree. Habitat: Riverine woodland, 1500–2900 m. Voucher: Sooboda 58 (EA).

***Racospermamearnsii* De Wild**. Habit: Tree. Habitat: Wooded grassland, 900–2600 m. Vouchers: FOKP 11561 (EA, HIB), Mbuni 298 (EA).

***Mimosamellifera* (M. Vahl) Benth.** Habit: Tree. Habitat: Bushland grassland, Dry woodland, 1–1800 m. Vouchers: SAJIT Z0064 (EA, HIB), FOKP 1252 (EA, HIB).

***Mimosanilotica* (L.) Delile** Habit: Tree. Habitat: Woodland grassland, 1–2300 m. Vouchers: Mus 169 (EA), Bogdan 4340 (EA), Collias 15382 (EA).

**Vachelliatortilissubsp.spirocarpa (A. Rich.) Brenan** Habit: Tree. Habitat: Bushland along streams, 200–1650 m. Voucher: FOKP 1249 (EA, HIB).

***Vachelliareficiens* Wawra** Habit: Tree. Habitat: Bushland, 50–1450 m. Voucher: FOKP 1257 (EA, HIB).

***Adenocarpusmannii* (Hook. f.) Hook. f.** Habit: Shrub. Habitat: Shrub Grassland, riverside, hearth, bamboo zone, 2400–3500 m. Voucher: SAJIT 007113 (EA, HIB).

***Albiziaanthelmintica* Brongn.** Habit: Tree. Habitat: Dry bushland, 1–3550 m. Voucher: Kazuaki 52 (EA).

***Argyrolobiumfischeri* Taub.** Habit: Herb. Habitat: Bushland, grassland, 1700–2700 m. Vouchers: SAJIT 005102 (EA, HIB), FOKP 1805 (EA, HIB).

***Argyrolobiumramosissimum* Baker**. Habit: Herb. Habitat: Forest margin, moorland, ericaceous, around 2300 m. Voucher: Rawlins 7 (EA).

***Astragalusatropilosulus* (Hochst.) Bunge.** Habit: Herb. Habitat: Open moorland, 1750–3550 m. Voucher: SAJIT 005084 (EA, HIB).

***Caesalpiniadecapetala* (Roth) Alston** Habit: Shrub. Habitat: Bushland, Forest edges, 650–2050 m. Voucher: FOKP 1297 (EA, HIB).

***Cassiaabbreviata* Oliv.** Habit: Shrub. Habitat: Bushland, 1–1000 m. Voucher: SAJIT 004734 (EA, HIB).

***Crotalariaanthyllopsis* Baker** Habit: Herb. Habitat: Wooded grassland, 1200–2250 m. Rocky places. Voucher: Tweedie 1921 (EA).

***Crotalariaaxillaris* Aiton** Habit: Shrub. Habitat: Secondary bushland, woodland, grassland 1–2250 m. Vouchers: FOKP 1870, 11414 & 11613 (EA, HIB), Mbuni 150 (EA), Part II Botany 13 (EA).

***Crotalariabrevidens* Benth.** Habit: Shrub. Habitat: Grassland, bushland, 1500–3000 m. Vouchers: FOKP 11482 (EA), Mbuni 218 (EA), Napier 1979 (EA).

***Crotalariachrysochlora* Harms** Habit: Herb. Habitat: Upland grassland, 1700–2900 m. Vouchers: Napier 1916 (EA), Webster 8786 (EA).

***Crotalariadeserticola* Baker f.** Habit: Shrub. Habitat: Open and wooded grassland, 100–2500 m. Vouchers: FOKP 11483 (EA, HIB), Mbuni 219 (EA).

***Crotalariafascicularis* Polhill** Habit: Shrub. Habitat: Dry evergreen forest, 1300–2300 m. Voucher: Champion 1 (EA).

***Crotalariaincana* L.** Habit: Shrub. Habitat: Upland grassland, 600–1800 m. Vouchers: FOKP 11347 (EA, HIB), Mbuni 083 (EA).

***Crotalarialachnocarpoides* Engl.** Habit: Shrub. Habitat: Bushed Grassland, forest thicket, forest margin, 1200–2650 m. Vouchers: FOKP 11573 (EA, HIB), Mbuni 310 (EA).

***Crotalariamicrocarpa* Benth.** Habit: Herb. Habitat: Bushland, Grassland, 100–1700 m. Voucher: Tweedie 2040 (EA).

***Crotalarianatalitia* Meissner** Habit: Herb. Habitat: Forest edges, bushland and wooded or grassland, 1300–2500 m. Voucher: Symes 116 (EA).

**Crotalariapallidavar.obovata (G. Don) Polhill.** Habit: Herb. Habitat: Bushland, near rivers and lakes, 450–1800 m. Voucher: Bogdan 4963 (EA).

***Crotalariapallida* Aiton** Habit: Herb. Habitat: Bushland near rivers, 1100–1900 m. Voucher: Tweedie 2097 (EA).

***Crotalariapodocarpa* DC** Habit: Herb. Habitat: Open sandy place, wooded grassland, 1300–1650 m. Voucher: Bogdan 5173 (EA).

***Crotalariastolzii* (Baker f.) Polhill** Habit: Herb. Habitat: Upland grassland, forest margin, 1800–3000 m. Voucher: Thulin & Tidigs 163 (EA).

***Dalbergialactea* Vatke** Habit: Shrub. Habitat: Riverine, 1050–2400 m. Vouchers: SAJIT 006880 (EA, HIB), FOKP 1289 (EA, HIB), Tweedie 1084 (EA).

***Desmodiumincanum* DC.** Habit: Herb. Habitat: Forest margin, 1450–2800 m. Vouchers: FOKP 11527 (EA, HIB), Mbuni 263 (EA).

***Desmodiumuncinatum* (Jacq.) DC.** Habit: Shrub. Habitat: Forest shade, 1400–2800 m. Vouchers: (FOKP 11715 EA, HIB), Mbuni 715 (EA).

***Dinebraretroflexa* (Vahl) Panz.** Habit: Herb. Habitat: Black clay soil, 500–2000 m. Voucher: Pratt 5716 (EA).

**Dolichossericeussubsp.formosus (A. Rich.) Verdc.** Habit: Climber. Habitat: Evergreen montane forest edge, 1150–2750 m. Vouchers: SAJIT 00686 (EA, HIB), Tweedie 2959 (EA).

***Dolichossericeus* E. Mey.** Habit: Herb. Habitat: Montane forest edge, 1150–2750 m. Voucher: Irwin 369 (EA).

***Entadaabyssinica* A. Rich.** Habit: Tree. Habitat: Wooded grassland, Riverine forest, 1450–2150 m. Vouchers: SAJIT 004842 & 006851 (EA, HIB), FOKP 1277 (EA, HIB).

***Entadaleptostachya* Harms.** Habit: Climber. Habitat: Dry bushland, 100–1350 m. Voucher: SAJIT 004842 (EA, HIB).

***Eriosemabuchananii* Baker f.** Habit: Herb. Habitat: upland grassland, 1900–2300 m. Voucher: Napier 1971 (EA).

***Eriosemacordifolium* A. Rich.** Habit: Herb. Habitat: Upland grassland about 2290 m. Voucher: Symes 526 (EA).

***Eriosemamacrostipulum* Baker f.** Habit: Herb. Habitat: Bushed grassland around 2100 m. Voucher: Symes 61 (EA).

***Eriosemamontanum* Baker f.** Habit: Herb. Habitat: Wooded grassland, 1500–3300 m. Voucher: Symes 203 (EA).

***Eriosemarhodesicum* R.E. Fr.** Habit: Herb. Habitat: Burnt upland grassland, rocky hill side, 1620–2400 m. Voucher: Symes 277 (EA).

***Erythrinaabyssinica* DC.** Habit: Tree. Habitat: Open woodland, forest edge, 900–2250 m. Vouchers: FOKP 11488 (EA, HIB), Mbuni 224 (EA), Trapnell 2302 (EA).

***Faidherbiaalbida* (Delile) A. Chev.** Habit: Tree. Habitat: Wooded grassland, 1500–2900 m. Voucher: Tweedie 2288 (EA).

***Galegalindblomii* (Harms) J.B. Gillett.** Habit: Herb. Habitat: Mountain grassland, 2200–2800 m. Vouchers: SAJIT Z0071, FOKP 949 & 1143, Geesteranus 6320 (EA), Gillett 18423 (EA).

***Glycinewrightii* Lopez.** Habit: Climber. Habitat: Disturbed ground, 2000–2900 m. Vouchers: FOKP 11450 (EA, HIB), Mbuni186 (EA).

***Hylodesmumrepandum* (Vahl) H. Ohashi & R.R. Mill** Habit: Herb. Habitat: Forest shade, medium altitude forest, 1450–2800 m. Vouchers: FOKP 11525 (EA, HIB), Mbuni 261 (EA).

***Indigoferaambelacensis* Schweinf**. Habit: Herb. Habitat: Grassland and bushland, 1000–2200 m. Vouchers: FOKP 11475 (EA, HIB), Mbuni 211 (EA).

***Indigoferaarrecta* A. Rich.** Habit: Herb. Habitat: Grassland and bushland and forest edges, 300–2700 m. Vouchers: FOKP 11497 & 11714 (EA, HIB), Mbuni 233 & 714 (EA), Bogdan 5358 (EA).

***Indigoferaastragalina* DC.** Habit: Herb. Habitat: Grazed grassland, 600–900 m. Vouchers: Bogdan 3410 (EA), Tweedie 2894 (EA).

***Indigoferabogdanii* J.B. Gillett** Habit: Shrub. Habitat: Dry grassland, 1500–2300 m. Voucher: Gillet 18423 (EA).

***Indigoferagarckeana* Vatke** Habit: Herb. Habitat: Grassland and bushland, 1000–2500 m. Vouchers: Agnew et al. 10388 (EA), Irwin 144 (EA).

***Indigoferahochstetteri* Baker.** Habit: Herb. Habitat: Dry grassland, 200–2000 m. Voucher: Bogdan 3842 (EA).

***Indigoferaschimperi* Jaub. & Spach.** Habit: Herb. Habitat: Grassland and bushland, 200–2200 m. Vouchers: FOKP 11747 (EA, HIB), Mbuni 747 (EA).

***Indigoferaspicata* Forssk**. Habit: Herb. Habitat: Grassland, 0–2900 m. Vouchers: FOKP 11502 (EA, HIB), Mbuni 238 (EA).

***Indigoferatrita* L. f.** Habit: Herb. Habitat: Grassland and bushland, 0–2500 m Vouchers: SAJIT 004747 (EA, HIB), FOKP 1288 (EA, HIB).

***Lablabpurpureus* (L.) Sweet**. Habit: Climber. Habitat: Riverine forest, 1–2400 m. Voucher: Webster 8788 (EA).

***Lonchocarpuseriocalyx* Harms** Habit: Tree. Habitat: Wooded grassland, 500–1680 m. Voucher: Bally 12334 (EA).

***Lotusbecquetii* Boutique.** Habit: Herb. Habitat: Rocky hill side, 2000–2800 m. Voucher: Strange 185 (EA).

***Macrotylomastipulosum* (Baker) Verdc.** Habit: Herb. Habitat: Wooded grassland, 1800–2150 m. Voucher: Symes 59 (EA).

***Neonotoniawightii* (Wight & Arn.) J.A. Lackey** Habit: Tree. Habitat: Bushland edges, 500–2100 m. Voucher: FOKP 1283 (EA, HIB).

***Neorautaneniamitis* (A. Rich.) Verdc.** Habit: Climber. Habitat: Dry bushland, 20–1800 m. Voucher: Padwa 291 (EA).

***Parochetuscommunis* D. Don.** Habit: Herb. Habitat: Moist area, 2200–3400 m. Voucher: FOKP 1908 & 1904 (EA, HIB).

***Pseudarthriaconfertiflora* (A. Rich.) Baker** Habit: Shrub. Habitat: Wooded grassland, 1–2200 m. Voucher: Webster 8793 (EA).

***Pterolobiumstellatum* (Forssk.) Brenan** Habit: Shrub. Habitat: Bushland, Woodland, 1000–2250 m. Voucher: FOKP 1265 (EA, HIB).

***Rhynchosiahirta* (Andrews) Meikle & Verdc.** Habit: Liana. Habitat: Dry woodland and bushland, 1–1800 m. Voucher: SAJIT 006841 (EA, HIB).

***Rhynchosiaorthobotrya* Harms**. Habit: Herb. Habitat: Dry rocky, bushland, 1000–2100 m. Voucher: Bogdan 3852 (EA).

***Rhynchosiaresinosa* (A. Rich.)** Baker. Habit: Liana. Habitat: Wooded grassland, bushland, 1530–2500 m. Voucher: Tweedie 1914 (EA).

***Rhynchosiausambarensis* Taub.** Habit: Herb. Habitat: Upland grassland forest edge, 1650–2400 m. Voucher: FOKP 1284 (EA, HIB).

***Sennabicapsularis* (L.) Roxb.** Habit: Shrub. Habitat: Wooded or bushed grassland, 1–1750 m. Voucher: Kuzuaki 9 (EA).

***Sennadidymobotrya* (Fresen.) H.S. Irwin & Barneby** Habit: Shrub. Habitat: Riverine, forest edge, 1500–2250 m. Vouchers: FOKP 11391 (EA, HIB), Mbuni 126 (EA).

***Sennahirsuta* (L.) H.S. Irwin & Barneby**. Habit: Shrub. Habitat: Bushed grassland 100–1750 m. Vouchers: FOKP 11422 (EA), Mbuni 158 (EA).

***Sennaobtusifolia* (L.) H.S. Irwin & Barneby**. Habit: Shrub. Habitat: Riverine, 1000–1750 m. Voucher: Brodhurst hill 677 (EA).

***Sennaoccidentalis* (L.) Link.** Habit: Shrub. Habitat: Cultivated ground, 250–1450 m. Vouchers: FOKP 11487 (EA), Mbuni 223 (EA), Ndegwa 974 (EA).

***Sennaseptemtrionalis* (Viv.) H.S. Irwin & Barneby** Habit: Shrub. Habitat: Riverine, grassland, waste places, 1450–2200 m. Voucher: FOKP 1903 (EA, HIB).

***Sesbaniasesban* (L.) Merr.** Habit: Shrub. Habitat: Water logged areas below 1900 m. Vouchers: FOKP 11493 (EA, HIB), Mbuni 229 (EA).

***Tamarindusindica* L.** Habit: Tree. Habitat: Riverine, bushland, 1–1500 m. Vouchers: Tweedie 65 (EA), Kazuaki 16 (EA).

***Tephrosiaholstii* Taub.** Habit: Herb. Habitat: Upland grassland, 1600–2600 m. Voucher: Symes 101 (EA).

***Tephrosiainterrupta* Engl**. Habit: Herb. Habitat: Scrub margin, 1400–3100 m. Vouchers: FOKP 11289 (EA), Mbuni 025 (EA), Thulin 120 (EA).

***Tephrosiavogelii* Hook. f.** Habit: Herb. Habitat: Waste ground up to 2300 m. Voucher: Hepper 5058) (EA)

***Teramnuslabialis* (L. f.) Spreng.** Habit: Climber. Habitat: Wooded grassland, 1700–2500 m. Voucher: Boonman 6627 (EA).

***Vignasubterranea* (L.) Verdc.** Habit: Herb. Habitat: Cultivated land below 1200 m. Voucher: FOKP 1716 (EA, HIB).

***Trifoliumburchellianum* Ser.** Habit: Herb. Habitat: Moist upland, 1600–3500 m. Vouchers: FOKP 951 (EA, HIB), Mabberley & McCall 242 (EA), Bogdan 4966 (EA).

***Trifoliumcheranganiense* J.B. Gillett** Habit: Herb. Habitat: Upland grassland, 2100–3100 m, Vouchers: Thorold 2759 (EA), Strange 48 (EA), Rawlins 2 (EA).

***Trifoliumcryptopodium* A. Rich.** Habit: Herb. Habitat: Grassland, 2100–3500 m. Vouchers: Thulin & Tidigs 113 (EA), Bogdan 4968 (EA).

***Trifoliumpolystachyum* Fresen.** Habit: Herb. Habitat: Forest margin, Swamp grassland, 1600–2800 m. Vouchers: Irwin 308 (EA), Symes 637 (EA).

***Trifoliumrueppellianum* Fresen.** Habit: Herb. Habitat: Upland grassland and moorland, 1700–3500 m. Vouchers: FOKP 1128 (EA, HIB), Strange 140 (EA).

***Trifoliumsimense* Fresen.** Habit: Herb. Habitat: Upland grassland, 2000–3500 m. Vouchers: Symes 638 (EA), Knight 56 (EA).

***Trifoliumsemipilosum* Fresen.** Habit: Herb. Habitat: Upland grassland, 1500–3000 m. Vouchers: FOKP 11501 (EA), Mbuni 237 (EA).

***Trifoliumusambarense* Taub.** Habit: Herb. Habitat: Forest opening, 1500–2700 m. Voucher: SAJIT Z0074 (HIB).

***Viciabenghalensis* L.** Habit: Climber. Habitat: Upland grassland 2400–2800 m. Voucher: Irwin 205 (EA).

***Vignamonophylla* Taub.** Habit: Herb. Habitat: Wooded grassland, 1650–2500 m. Voucher: Symes 86 (EA).

**Vignaunguiculatasubsp.dekindtiana (Harms) Verdc.** Habit: Herb. Habitat: Bushland and forest edges, 1–2500 m. Voucher: Lyes 57 (EA).

***Vignavexillata* (L.) A. Rich**. Habit: Herb. Habitat: Grassland, bushland and forest margins, 100–2500 m. Voucher: Symes 212 (EA).

***Zorniaglochidiata* DC.** Habit: Herb. Habitat: Grassland, rocky areas, 0–1800 m. Voucher: Bogdan 3450 (EA).

**F46. Polygalaceae**

1 Genus 4 Species

***Polygalaalbida* Schinz** Habit: Herb. Habitat: Field of Weeds and roadside, 1400–2300 m. Voucher: Symes 758 (EA).

***Polygalaarenaria* Willd.** Habit: Herb. Habitat: Disturbed ground, 60–1200 m. Voucher: Lucas 218 (EA).

***Polygalapetitiana* A. Rich.** Habit: Herb. Habitat: Shallow soils on grassland, 100–2300 m. Voucher: Symes 158 (EA).

***Polygalasphenoptera* Fresen.** Habit: Herb. Habitat: Upland grassland, disturbed soil on wet forest, 100–3200 m. Vouchers: SAJIT 005100 & Z0051 (EA, HIB), Mbuni 087 (EA), Carter & Stannard 48 (EA), Mabberley & McCall 279 (EA), Symes 217 (EA), Webster 8717 (EA).

**F47. Rosaceae**

5 Genera, 15 Species

***Alchemillacryptantha* Steud. ex A. Rich.** Habit: Herb. Habitat: Montane grassland 2900–3500 m. Vouchers: Napier 1978 (EA), Knox 4185 & 4193 (EA).

**Alchemillaelgonensis Mildbr.** Habit: Shrub. Habitat: Montane grassland, 3000–3500 m. Voucher: Thulin & Tidigs 248 (EA).

***Alchemillaellenbeckii* Engl.** Habit: Shrub. Habitat: Highland grassland, 2100–3500 m. Voucher: Knox 3384 (EA).

***Alchemillagracilipes* (Engl.) Engl.** Habit: Herb. Habitat: Montane grassland 2120–3500 m. Vouchers: SAJIT 006928 (EA, HIB), Dale 3400 (EA), Symes 619 (EA), Verdcourt 2426 (EA).

***Alchemillajohnstonii* Oliv.** Habit: Shrub. Habitat: Alpine forest, 3150–3350 m. Vouchers: Dale 340, Thulin & Tidigs 212 & 258 (EA), Knox 3385 (EA).

***Alchemillalindblomiana* (Mildbr.) T.C.E. Fr.** Habit: Shrub. Habitat: 3000–3300 m. Voucher: Dale 3401(EA)

***Alchemillarothii* Oliv.** Habit: Herb. Habitat: Bamboo zones, 2100–3500 m. Vouchers: Mabberley 579 (EA), Knox 3404 (EA).

***Cliffortianitidula* (Engl.) R.E. Fr. & T.C.E. Fr.** Habit: Shrub. Habitat: Bamboo glades, moorland, 2920–3490 m. Voucher: Dale 3432 (EA).

***Hageniaabyssinica* (Bruce ex Steud.) J.F. Gmel.** Habit: Tree. Habitat: Woodland, 2300–3300 m. Voucher: SAJIT 004854 (EA, HIB).

***Prunusafricana* (Hook. f.) Kalkman** Habit: Tree. Habitat: Woodland, Riverine, 1350–2750 m. Vouchers: SAJIT 006869 (EA, HIB), FOKP 1019, 10969 & 11609 (EA, HIB), Mbuni 609 (EA).

***Rubusapetalus* Poir.** Habit: Shrub. Habitat: Upland bushland, 1600–3000 m. Vouchers: FOKP 1060 & 11274 (EA, HIB).

***Rubuspinnatus* Willd.** Habit: Trailing Shrub. Habitat: Upland rain forest, bamboo 2400–3000 m. Voucher: Tweedie 4086 (EA).

***Rubusscheffleri* Engl.** Habit: Shrub. Habitat: Upland rain forest glades, 1800–2930 m. Vouchers: Mabberley 496 (EA), Lindsay 137 (EA).

***Rubussteudneri* Schweinf**. Habit: Shrub. Habitat: Montane forest, 2000–3480 m. Vouchers: SAJIT 003385, 005121 & 006811 (EA, HIB), FOKP 1010 & 10911 (EA, HIB), Beentje 3065 (EA), Tweedie 4086 (EA).

***Rubusvolkensii* Engl.** Habit: Shrub. Habitat: Secondary bushland, bamboo zones, 2600–3500 m. Vouchers: SAJIT 004800 (EA, HIB), Thulin & Tidigs 242 (EA).

**F48. Rhamnaceae**

6 Genera, 8 Species

***Gouanialongispicata* Engl.** Habit: Shrub. Habitat: Wooded grassland, 1450–2400 m. Vouchers: SAJIT 005057 (EA, HIB), FOKP 955, 1030, 2335 & 11278 (EA, HIB).

***Helinusintegrifolius* (Lam.) Kuntze** Habit: Shrub. Habitat: Bushland, open woodland, 300–1330 m. Voucher: FOKP 1098 (EA, HIB).

***Helinusmystacinus* (Aiton) E. Mey. ex Steud.** Habit: Shrub. Habitat: 1000–1500 m. Voucher: FOKP 1098 (EA, HIB).

***Rhamnusprinoides* L’Hér.** Habit: Tree. Habitat: Bushland, Bamboo, heath zone, 1500–3150 m. Vouchers: SAJIT 004817 & 005080 (EA, HIB), FOKP 1001, 1226, 11310 & 11366, (EA, HIB), Mbuni 046 (EA).

***Rhamnusstaddo* A. Rich.** Habit: Shrub. Habitat: Bushland, Grassland, 1500–2950 m. Vouchers: FOKP 11310 & 11652 (EA, HIB), Mbuni 046 & 652 (EA).

***Scutiamyrtina* (Burm. f.) Kurz** Habit: Shrub. Habitat: Moist, dry forest, riverine up to 2750 m. Vouchers: SAJIT 005064 (EA, HIB), FOKP 943, 11266, 11309 & 11378 (EA, HIB), Mbuni 045 (EA).

***Ziziphusabyssinica* Hochst. ex A. Rich.** Habit: Tree. Habitat: Bushland, woodland, 700–2200 m. Voucher: FOKP 11318 (EA, HIB).

***Ziziphusmucronata* Willd.** Habit: Tree. Habitat: Bushland, Wooded Grassland, 1–1950 m. Voucher: FOKP 11334 (EA, HIB).

**F49. Ulmaceae**

1 Genus, 1 Species

***Chaetachmearistata* Planch.** Habit: Tree. Habitat: Bushland, 1050–2100 m. Voucher: FORK 927 (EA, HIB).

**F50. Cannabaceae**

2 Genera, 2 Species

***Celtisafricana* Burm. f.** Habit: Tree. Habitat: Riverine forest, 1150–2400 m. Voucher: Verdcourt 11725 (EA).

***Tremaorientalis* (L.) Blume** Habit: Tree. Habitat: Wooded grassland, forest edges, 1–1800 m. Vouchers: FOKP 11562 (EA), Mbuni 299 (EA).

**F51. Moraceae**

2 Genera, 8 Species

***Ficusglumosa* Delile** Habit: Tree. Habitat: Rocky hillsides, 450–2050 m. Vouchers: SAJIT 006836 (EA, HIB), FOKP 10936 (EA, HIB).

***Ficussur* Forssk.** Habit: Tree. Habitat: Riverine, Bushland, 1–2100 m. Vouchers: SAJIT 006849 (EA, HIB), FOKP 1273 & 10949 (EA, HIB).

***Ficusexasperata* Vahl** Habit: Tree. Habitat: Wet forest up to 1850 m. Voucher: FOKP 920 (EA, HIB).

***Ficusnatalensis* Hochst.** Habit: Tree. Habitat: Riverine, 900–1800 m. Voucher: FOKP 1032 (EA, HIB).

***Ficusthonningii* Blume** Habit: Tree. Habitat: Wooded grassland, 1050–2400 m. Vouchers: SAJIT 005149 (EA, HIB).

***Dorsteniaafromontana* R.E. Fr.** Habit: Herb. Habitat: Wet montane forest, 2000–2600 m. Voucher: FOKP 1814 (EA, HIB).

**Dorsteniahildebrandtiivar.schlechteri (Engl.) Hijman** Habit: Herb. Habitat: Riverine forest, 275–2100 m. Vouchers: FOKP 11671 (EA, HIB), Mbuni 671 (EA), Napier 1915 (EA).

**Dorsteniabarnimianavar.tropaeolifolia (Schweinf.) Rendle** Habit: Herb. Habitat: Open savannah, common everywhere in short grass, Stony upland, 1000–2100 m. Voucher: Symes 627 (EA).

**F52. Urticaceae**

8 Genera, 13 Species

***Droguetiadebilis* Rendle** Habit: Herb. Habitat: Highland forest, 275–2100 m. Voucher: FOKP 1875 (EA, HIB).

***Droguetiainers* (Forssk.) Schweinf.** Habit. Herb. Habitat: Lower edge bamboo, 1600–3250m. Vouchers: SAJIT 004828 & 006920 (EA, HIB), FOKP 11020 (EA, HIB).

***Elatostemamonticola* Hook. f.** Habit: Herb. Habitat: Wet places, 1600–2800 m. Vouchers: SAJIT 006890 (EA, HIB), FOKP1735 & 10990 (EA, HIB).

***Laporteaalatipes* Hook. f.** Habit: Herb. Habitat: Disturbed ground in wet montane forest, 1560–3000 m. Habitat: Wet mountain forest, 1560–3010 m. Voucher: FOKP 1080 (EA, HIB).

***Laporteaovalifolia* (Schumach. & Thonn.) Chew.** Habit: Herb. Habitat: Disturbed woodland, 900–2910 m. Voucher: SAJIT 007125 (EA, HIB).

***Parietariadebilis* G. Forst.** Habit: Herb. Habitat: Montane forest, 1560–3010 m. Voucher: Tweedie 2698 (EA).

***Pileaangolensis* (Hiern) Rendle** Habit: Herb. Habitat: streamsides, 1400–2800 m Voucher: SAJIT 005090 (EA, HIB).

***Pileajohnstonii* Oliv.** Habit: Herb. Habitat: streamsides 1680–2910 m. Voucher: Part II Botany 52 (EA).

***Pilearivularis* Wedd.** Habit: Herb. Habitat: Montane forest, Streams & Paths, 1480–3100 m. Voucher: SAJIT 004827 (EA, HIB).

***Pileatetraphylla* (Steud.) Blume** Habit: Herb. Habitat: Montane forest, 1750–2480 m. Voucher: Gilbert & Meifin 6467 (EA).

***Urerahypselodendron* (Hochst. ex A. Rich.) Wedd.** Habit: Climber. Habitat: Montane forest, moist upland forest, 1480–3100 m. Vouchers: SAJIT 004822, 005082 & 006866 (EA, HIB), FOKP 952, 964, 11358, 11377 & 11617 (EA, HIB), Mbuni 114 & 617 (EA).

***Urticamassaica* Mildbr.** Habit: Herb. Habitat: Montane forest, 2000–3400 m. Vouchers: FOKP 961, 11312 & 11326 (EA, HIB), Mbuni 062 (EA).

***Pouzolziaparasitica* (Forssk.) Schweinf.** Habit: Herb. Habitat: Upland forest 1050–2100 m. Voucher: FOKP 1880 (EA, HIB).

**F53. Cucurbitaceae**

6 Genera, 8 Species

***Cocciniaadoensis* (Hochst. ex A. Rich.) Cogn.** Habit: Climber. Habitat: Highland grassland, 1600–2300 m. Vouchers: Napier 1899 (EA), Symes 283 (EA).

***Cocciniagrandis* (L.) Voigt** Habit: Climber. Habitat: Riverine, 250–1650 m Vouchers: SAJIT 006918 (EA, HIB), FOKP 11018 (EA, HIB).

***Cucumisficifolius* A. Rich.** Habit: Climber. Habitat: Highland grassland, 1000–1650 m. Voucher: Symes 273 (EA).

***Momordicafoetida* Schumach.** Habit: Climber. Habitat: Grassland, Woodland 150–1650 m. Vouchers: SAJIT 004743 & 006882 (EA, HIB), FOKP 11293 (EA, HIB), Mbuni 656 & 716 (EA).

***Peponiumvogelii* (Hook. f.) Engl.** Habit: Climber. Habitat: Forest edge, 10–2600 m. Vouchers: SAJIT 006870 (EA, HIB), FOKP 1256, 1269 & 11428 (EA, HIB), Mbuni 164 (EA), Brodhust hill 537 (EA), Napier 1975 (EA).

**Trochomeriamacrocarpasubsp.vitifolia R. Fern. & A. Fern.** Habit: Climber. Habitat: Grassland, 1000–2500 m. Voucher: Lindsay 130 (EA).

***Zehneriaanomala* C. Jeffrey** Habit: Climber. Habitat: 1000–2500 m. Vouchers: FOKP 11328 (EA, HIB), Mbuni 064 (EA).

***Zehneriascabra* Sond.** Habit: Climber. Habitat: Bushland, Forest edges, 1000–3300 m. Vouchers: SAJIT 004721, 006799 & 006872 (EA, HIB), FORK 933, 1005, 10899 & 11462 (EA, HIB), Mbuni 064 & 198 (EA).

**F54. Bignoniaceae**

1 Genus, 1 Species

***Stereospermumkunthianum* Cham.** Habit: Tree. Habitat: Rocky bushland, wooded grassland, 900–2100 m. Habitat: Voucher: Bogdan 265 (EA).

**F55. Celastraceae**

6 Genera, 11 Species

***Cathaedulis* (Vahl) Endl.** Habit: Tree. Habitat: Wooded grassland, 1200–2400 m. Vouchers: Munro 989 (EA), Agnew 5087 (EA).

***Cassineaethiopica* Thunb.** Habit: Shrub. Habitat: Bushland, 900–2400 m. Vouchers: FOKP 11608 (EA, HIB), Mbuni 608 (EA).

***Gymnosporiabuchananii* Loes.** Habit: Shrub. Habitat: Grassland, 1–2650 m. Vouchers: FOKP 11294 (EA, HIB), Birch 61 & 180 (EA).

***Gymnosporiaheterophylla* (Eckl. & Zeyh.) Loes.** Habit: Shrub. Habitat: Dry Upland & lowland Forest, Riverine, wooded grassland, 1150–2700 m. Vouchers: FOKP 11601 (EA, HIB), Mbuni 144 & 601 (EA, HIB).

***Gymnosporiakeniensis* (Loes.) Jordaan** Habit: Shrub Habitat: Dry Upland & lowland Forest, Riverine, thickets, Woodland, 1350–2250 m. Vouchers: SAJIT 004826 (EA, HIB), Fries & Hansen 2549 (EA).

***Gymnosporiasenegalensis* (Lam.) Loes.** Habit: Tree. Habitat: Bushed grassland, Riverine, 1–2100m. Vouchers: FOKP 11376 (EA), Buich 61 & 180 (EA), Lind et al. 5082 (EA), Webster 8812 (EA).

***Hippocrateagoetzei* Loes.** Habit: Shrub. Habitat: Woodland, forest thicket, 1100–2700 m. Voucher: FOKP 1806 (EA, HIB).

***Maytenusarbutifolia* (Hochst. ex A. Rich.) R. Wilczek** Habit: Tree. Habitat: Bushland, Grassland, 1600–2350 m. Vouchers: SAJIT 004757 (EA, HIB), FOKP 1271 (EA, HIB).

***Maytenusobscura* (A. Rich.) Cufod.** Habit: Shrub. Habitat: Bushland, 1900–2550 m. Vouchers: FOKP 1089, 1235 (EA, HIB).

***Maytenusundata* (Thunb.) Blakelock** Habit: Tree. Habitat: Evergreen forest, dry Bushland, Grassland, 1500–3200 m. Vouchers: SAJIT 004755 (EA, HIB), FOKP 1015 (EA, HIB).

***Simirestisbrianii* N. Hallé** Habit: Tree. Habitat: Forest margin, 500–2550 m. Voucher: SAJIT Z0110 (HIB).

**F56. Oxalidaceae**

1 Genus, 4 Species

***Oxalisanthelmintica* A. Rich.** Habit: Herb. Habitat: Grassland, Riverine 840–2400 m. Voucher: Symes 58 (EA).

***Oxaliscorniculata* L**. Habit: Herb. Habitat: Grassland, Disturbed ground, 0–3500 m. Voucher: Townsend 2374 (EA).

***Oxalisobliquifolia* Steud. ex A. Rich.** Habit: Herb. Habitat: Grazer pasture, Grassland, roadsides, 830–3300 m. Voucher: Thulin 81 (EA).

***Oxalisradicosa* A. Rich.** Habit: Herb. Habitat: Weed of cultivation and disturbed ground, 830– 2500 m. Voucher: Symes 46 (EA).

**F57. Rhizophoraceae**

1 Genus, 3 Species

***Cassipoureamalosana* (Baker) Alston** Habit: Tree. Habitat: Bushland, Understorey in moist forest, 750–2900 m. Voucher: SAJIT 004730 (EA, HIB).

***Cassipourearotundifolia* (Engl.) Alston.** Habit: Tree. Habitat: Woodland, 900–1700 m. Voucher: SAJIT 007077 (EA, HIB).

***Cassipourearuwensorensis* (Engl.) Alston** Habit: Tree. Habitat: Evergreen forest, woodland, 1450–1600 m, Voucher: FOKP 1125 (EA, HIB).

**F58. Peraceae**

1 Genus, 1 Species

***Clutiaabyssinica* Jaub. & Spach** Habit: Shrub; Habitat: Secondary bushland, 1600–3100 m. Vouchers: SAJIT 007042 (EA, HIB), FOKP 1035 & 11283 (EA, HIB), Mbuni 019 (EA).

**F59. Euphorbiaceae**

13 Genera, 30 Species

***Acalyphafruticosa* Forsk.** Habit: Shrub. Habitat: Lowland forest edge, 200–2000 m. Vouchers: FOKP 11676 (EA, HIB), Mbuni 676 (EA).

***Acalyphavolkensii* Pax** Habit: Shrub. Habitat: Upland grassland, 800–2700 m Vouchers: SAJIT 004732 & 006867 (EA, HIB), FOKP 11407 (EA, HIB), Mbuni 143 & 712 & (EA).

***Acalyphapolymorpha* Müll. Arg.** Habit: Herb. Habitat: Wooded, grassland, 1700–2300 m. Voucher: Symes 34 (EA).

***Crotondichogamus* Pax** Habit: Tree. Habitat: Dry bushland, Disturbed sites, 500–2100 m. Vouchers: FOKP 11466 & 11658 (EA, HIB), Mbuni 202 & 658 (EA).

***Crotonmacrostachyus* Hochst. ex Delile** Habit: Tree. Habitat: Wooded Grassland, dry or moist upland forest, 1350–2250 m. Vouchers: FOKP 1150 & 11697 (EA, HIB), Mbuni 236 & 697 (EA), Birch 61 & 169 (EA).

***Cordiamonoica* Roxb.** Habit: Shrub. Habitat: Deciduous bushland, grassland, 1–2100 m. Vouchers: SAJIT Z006 (HIB).

***Erythrococcabongensis* Pax** Habit: Tree. Habitat: Wooded Bushland, River line or thicket, 900–2100 m. Vouchers: FOKP 11430 (EA, HIB), Mbuni 166 (EA).

***Erythrococcafischeri* Pax** Habit: Shrub. Habitat: Woodland, grassland, 1500–2300 m. Vouchers: FOKP 1028 & 1234 (EA, HIB).

***Euphorbiaabyssinica* J.F. Gmel.** Habit: Tree. Habitat: Woodland, 2100–2400 m. Vouchers: SAJIT 006883 (EA, HIB), Haper & Field 4975 (EA), Mabberley 497 (EA).

***Euphorbiaampliphylla* Pax** Habit: Tree. Habitat: Woodland, grassland, 1500–2300 m. Voucher: Mabberley 497 (EA).

***Euphorbiabongensis* Kotschy & Peyr. ex Boiss.** Habit: Shrub. Habitat: Rare in grassland, 1200–2000 m. Voucher: Napper & Tweedie 2115 (EA).

***Euphorbiabrevicornu* Pax** Habit: Herb. Habitat: Drier highlands, 2200–3600 m. Voucher: Mabberley & McCall 254 (EA).

***Euphorbiacyparissioides* Pax** Habit: Herb. Habitat: Grassland, 1900–2250 m. Voucher: Webster 8773 (EA).

***Euphorbiadepauperata* Hochst. ex A. Rich.** Habit: Shrub. Habitat: Highland grassland, 1900–3200 m. Voucher: Thulin & Tidigs 224 (EA).

***Euphorbiaengleri* Pax** Habit: Herb. Habitat: Shade of montane forest, 1500–2800 m. Voucher: FOKP 1807 (EA, HIB).

***Euphorbiaheterospina* S. Carter** Habit: Shrub. Habitat: Woodland, 900–1800 m. Voucher: FOKP 1247 (EA, HIB).

***Euphorbiarepetita* Hochst. ex A. Rich.** Habit: Herb. Habitat: Grassland, 2300–2700 m. Vouchers: Thulin & Tidigs 142 (EA), Agnew et al. 10530 (EA).

***Euphorbiascarlatina* S. Carter** Habit: Shrub. Habitat: Bushland on rocky slopes, 600–2000 m. Voucher: SAJIT 006875 (EA, HIB).

***Euphorbiaschimperiana* Scheele** Habit: Herb. Habitat: Grassland, montane 1600–3500 m. Vouchers: SAJIT 005113 (EA, HIB), Thulin & Tidigs 142 (EA).

***Euphorbiatirucalli* L.** Habit: Shrub. Habitat: Dry bushland, 0–1600 m. Voucher: Tweedie 2101 (EA).

***Euphorbiaumbellata* (Pax) Bruyns** Habit: Tree. Habitat: Dry forest, riverine, 1150–2100 m. Voucher: Glover et al. 1969 (EA).

***Macarangacapensis* (Baill.) Sim** Habit: Tree Habitat: Moist forest, 300–2100 m. Voucher: SAJIT 004824 (EA, HIB).

***Macarangakilimandscharica* Pax** Habit: Tree. Habitat: Moist upland forest, 1650–2400 m. Vouchers: SAJIT 006797 (EA, HIB), FORK 991, 1000, 10896 & 11349 (EA, HIB), Birch 175 (EA), Buch 61 & 175 (EA).

**Margaritariadiscoideavar.fagifolia (Pax) Radcl.–Sm.** Habit: Herb. Habitat: Upland forest, 10–1700 m. Voucher: Obunyali & Omondi 0226 (EA).

***Micrococcamercurialis* (L.) Benth.** Habit: Herb. Habitat: Dry bushland, 10–1610 m. Voucher: Bogdan 3847 (EA).

***Mondiawhitei* (Hook. f.) Skeels.** Habit: Shrub. Habitat: Forest thickets, 800–2200 m. Voucher: FOKP 1718 (EA, HIB).

***Neoboutoniamacrocalyx* Pax** Habit: Tree. Habitat: Upland forest, evergreen forest, Riverbanks 1600–2700 m. Vouchers: SAJIT 005087 & 006798 (EA, HIB), FOKP 11351 & 11523 (EA, HIB), Mbuni 259 (EA), Tweedie 4118 (EA).

***Ricinuscommunis* L.** Habit: Herb. Habitat: Bushland, Grassland, 600–3100 m. Voucher: Mas 236 (EA).

***Shirakiopsiselliptica* (Hochst.) Esser** Habit: Tree. Habitat: Woodland, 1400–1800 m. Voucher: Birch 177 (EA).

***Tragiabrevipes* Pax** Habit: Herb. Habitat: Dry forest, 600–2600 m. Vouchers: SAJIT 004744 (EA, HIB), FOKP 1059 & 11753 (EA, HIB), Mbuni 753 (EA).

**F60. Ochnaceae**

1 Genus, 2 Species

***Ochnainermis* (Forssk.) Schweinf. ex Penz.** Habit: Shrub. Habitat: Bushland, 1–1450 m. Voucher: Carter & Stannard 70 (EA).

***Ochnathomasiana* Engl. & Gilg** Habit: Tree. Habitat: Bushland, woodland, 1500–2400 m. Vouchers: FOKP 11628 (EA), Mbuni 628 (EA).

**F61. Phyllanthaceae**

2 Genera, 7 Species

***Brideliascleroneura* Müll. Arg.** Habit: Shrub. Habitat: Wooded grassland, 900–1650 m. Voucher: Pole & Erens 1519 (EA).

***Phyllanthusfischeri* Pax** Habit: Shrub. Habitat: Woodland, Forest edge, river line bushes, 1450–2700 m. Vouchers: SAJIT 004778 (EA, HIB), FOKP 11370 (EA, HIB), Mbuni 106 (EA), Thulin & Tidigs 122 (EA), Agnew et al. 10493 (EA), Hepper 4980 (EA).

***Phyllanthusovalifolius* Forssk**. Habit: Shrub. Habitat: Bushland, 1350–2450 m. Vouchers: FOKP 11459 & 11679 (EA, HIB), Mbuni 195 & 679 (EA).

***Phyllanthussepialis* Müll. Arg.** Habit: Shrub. Habitat: Riverine Woodland, Bushland, 600–1950 m. Voucher: FOKP 11384 (EA, HIB).

***Phyllanthussuffrutescens* Pax** Habit: Herb. Habitat: Grassland 1200–2350 m. Voucher: Napier 1911 (EA).

***Phyllanthusniruroides* Müll. Arg.** Habit: Herb. Habitat: Grassland, 1200–2350 m. Voucher: Agnew et al. 10318 (EA).

***Phyllanthusboehmii* Pax** Habit: Herb. Habitat: Grassland moorland, 2000–3270 m. Voucher: FOKP 960 (EA, HIB).

**F62. Passifloraceae**

1 Genus, 1 Species

***Adeniacissampeloides* (Planch. ex Hook.) Harms** Habit: Climber. Habitat: Moist forest, 0–2500 m. Voucher: SAJIT 004724 (EA, HIB).

**F63. Salicaceae**

4 Genera, 5 Species

***Caseariabattiscombei* R.E. Fr.** Habit: Tree. Habitat: Upland moist forest, 1350–2400 m. Vouchers: SAJIT 006907 (EA, HIB), FOKP 1122 & 11007 (EA, HIB), Mbuni 588 & 618 (EA).

***Dovyalisabyssinica* (A. Rich.) Warb.** Habit: Tree. Habitat: Upland moist forest, 1450–2600 m. Vouchers: FOKP 11365 (EA), Mbuni 101 (EA).

***Dovyalismacrocalyx* (Oliv.) Warb.** Habit: Shrub. Habitat: Moist forest, grassland 120–2400 m. Vouchers: FOKP 11678 (EA), Mbuni 678 (EA).

***Flacourtiaindica* (Burm. f.) Merr.** Habit: Tree. Habitat: Wooded grassland, 1200–2400 m. Vouchers: FOKP 1264, 11288 & 11321 (EA, HIB), Mbuni 741 (EA), Brunt 1410 (EA), Kazuaki 15 (EA), Buch 164 (EA).

***Trimeriagrandifolia* (Hochst.)** Habit: Tree. Habitat: Bushland 150–2500 m. Vouchers: FOKP 11672 (EA), Mbuni 672 (EA), Beentje 3044 (EA).

**F64. Violaceae**

1 Genus, 3 Species

***Violaeminii* (Engl.) R.E. Fr.** Habit: Herb. Habitat: Forest paths & streamside, 2100–3540 m. Voucher: Mabberley & McCall 240 (EA).

***Violaabyssinica* Steud. ex Oliv.** Habit: Herb. Habitat: Alpine, along paths, 1600–3500 m. Voucher: Townsend 2383 (EA).

***Violavillosa* Walter** Habit: Climber. Habitat: Forest paths & streamside, 2100–3540 m. Voucher: Irwin 205 (EA).

**F65. Linaceae**

1 Genus, 1 Species

***Linumvolkensii* Engl.** Habit: Herb. Habitat: Upland grassland, Roadsides & waste places, 1100–2900 m. Voucher: Agnew et al. 10504 (EA).

**F66. Hypericaceae**

1 Genus, 6 Species

***Hypericumkiboense* Oliv.** Habit: Shrub. Habitat: Bamboo zone, 2250–3250 m. Voucher: Mabberley 582 (EA).

***Hypericumlalandii* Choisy.** Habit: Herb. Habitat: Grassland, 1500–2300 m. Voucher: Symes 615 (EA).

***Hypericumpeplidifolium* A. Rich.** Habit: Herb. Habitat: Subalpine grassland, 1350–3500 m. Voucher: Napier 1927 (EA), Mabberley 7 (EA), McCall 192 (EA), Symes 45 (EA).

***Hypericumquartinianum* A. Rich.** Habit: Shrub. Habitat: Rocky stream-banks, 1500–2250 m. Voucher: FOKP 11268 (EA, HIB).

***Hypericumrevolutum* Vahl** Habit: Shrub. Habitat: Heath zone, 2700–3500 m. Vouchers: SAJIT 004812 (EA, HIB), FOKP 992, 1087 & 11773 (EA, HIB), Mbuni 001 (EA), Knox 4174 (EA), Kokwaro 2532 (EA).

***Hypericumscioanum* Chiov.** Habit: Herb. Habitat: Montane forest, 1850–3500 m. Vouchers: SAJIT 005120 (EA, HIB), Knox 3451 & 3364 (EA).

**F67. Geraniaceae**

2 Genera, 4 Species

***Geraniumvagans* Baker** Habit: Herb. Habitat: Grassland in montane, 2640–3500 m. Voucher: Beentje 3072 (EA).

***Geraniumaculeolatum* Oliv.** Habit: Herb. Habitat: Montane forest 2640–3500 m. Vouchers: SAJIT 005030 (EA, HIB), Mabberley 255 (EA).

***Geraniumarabicum* Forssk.** Habit: Herb. Habitat: Montane forest, 1800–3550 m. Voucher: FOKP 1300, Napier 1972 (EA).

***Pelargoniumalchemilloides* (L.) Aiton** Habit: Herb. Habitat: Wooded grassland montane forest edge, 1000–2100 m. Voucher: FOKP 1825 (EA).

**F68. Melianthaceae**

1 Genus, 1 Species

***Bersamaabyssinica* Fresen.** Habit: Tree. Habitat: Dry & wet montane forest, 1150–2550 m. Vouchers: SAJIT 004818, 005071 & 007049 (EA, HIB), FOKP 941, 1263, 11284 & 11410 (EA, HIB), Mbuni 146 & 708 (EA).

**F69. Combretaceae**

2 Genera, 6 Species

***Combretumapiculatum* Sond.** Habit: Tree. Habitat: Wooded grassland, 250–1750 m. Voucher: SAJIT 004849 (EA).

**Combretumcollinumsubsp.binderianum (Kotschy) Okafa** Habit: Tree. Habitat: Wooded grassland, 700–2200 m. Voucher: Lucas 156 (EA).

***Combretumcapituliflorum* Fenzl ex Schweinf.** Habit: Liana. Habitat: Wooded grassland, Riverine forest, 900–1100 m. Voucher: Wilson 310 (EA).

***Combretummolle* R. Br. ex G. Don** Habit: Tree. Habitat: Wooded grassland, 150–2100 m. Vouchers: FOKP 1102, 11270 & 11682 (EA, HIB).

***Terminalia boivinii* Tul.** Habit: Tree. Habitat: Woodland, 700–1900 m. Voucher: SAJIT Z0055 (EA, HIB).

***Terminalia brownii* Fresen.** Habit: Tree. Habitat: Bushland, Woodland 700–1900 m. Voucher: FOKP 11342 (EA, HIB).

**F70. Lythraceae**

2 Genera, 2 Species

***Rotalatenella* (Guill. & Perr.) Hiern** Habit: Herb. Habitat: Grassland, 1250–2100 m. Vouchers: SAJIT 006913 (EA, HIB), FOKP 11013 (EA, HIB).

***Woodfordiauniflora* (A. Rich.) Koehne** Habit: Shrub. Habitat: Bushland, Rocky slopes, along dry rivers, 1350–2300 m. Voucher: Gardner 3717 (EA).

**F71. Onagraceae**

2 Genera, 4 Species

***Epilobiumhirsutum* L.** Habit: Herb. Habitat: Swamps, upland grassland, 1800–2450 m. Vouchers: FOKP 11332 & 11720 (EA, HIB), Mbuni 068, 720 (EA).

***Epilobiumsalignum* Hausskn.** Habit: Herb. Habitat: Swampy places, 1800–3550 m. Voucher: Brodhurst 505 (EA).

***Epilobiumstereophyllum* Fresen.** Habit: Herb. Habitat: Upland grassland, 2600–3500 m. Vouchers: Symes 626 (EA), Thulin et al. 106 (EA), Muthama et al. 170 (EA).

***Ludwigiaabyssinica* A. Rich.** Habit: Herb. Habitat: Swampy places, 1000–2300 m. Vouchers: FOKP 974 (EA, HIB), Symes 517 (EA).

**F72. Myrtaceae**

2 Genera, 3 Species

***Psidiumguajava* L.** Habit: Tree. Habitat: Bushland, 1000–2500 m. Vouchers: Mbuni 207 (EA), FOKP 11471 (EA).

***Syzygiumguineense* (Willd.) DC.** Habit: Tree. Habitat: Wooded grassland, Riverine, 1–2500 m. Vouchers: SAJIT 005086 (EA, HIB), FOKP 11612 (EA), Mbuni 612 (EA), Thomas 2110 (EA), Dale 902 (EA).

***Syzygiumcordatum* Hochst. ex Krauss** Habit: Tree. Habitat: Riverine, 1–2500 m. Vouchers: FOKP 11272, 11443 & 11669 (EA, HIB), Mbuni 179 & 669(EA).

**F73. Melastomataceae**

2 Genera, 6 Species

***Antherotomanaudinii* Hook. f.** Habit: Herb. Habitat: Shallow soils, watercourses, 500–2300 m. Voucher: Bogdan 5280 (EA).

***Dissotisdebilis* Triana** Habit: Herb. Habitat: Swampy grassland, 10–1800 m. Voucher: FOKP 1242 (EA, HIB).

***Dissotisspeciosa* Taub.** Habit: Shrub. Habitat: Swampy areas, 1000–2250 m. Vouchers: Verdcourt 734 (EA), Webster 8749, Gardner 3718 (EA).

***Dissotiscanescens* (E. Mey. ex Graham) Hook. f.** Habit: Herb. Habitat: Swampy grassland, 800–2200 m. Vouchers: Webster 748 & 8747 (EA).

***Dissotissenegambiensis* (Guill. & Perr.) Triana** Habit: Herb. Habitat: Grassland, 1350–2650 m. Voucher: Webster 8746 (EA).

***Dissotisirvingiana* Hook.** Habit: Herb. Habitat: Swampy grassland, 800–2200 m. Voucher: Webster 8745 (EA).

**F74. Penaeaceae**

1 Genera, 1 Species

***Oliniarochetiana* A. Juss.** Habit: Tree. Habitat: Dry upland forest, 1700–3050 m. Vouchers: FOKP 1024 & 1216 (EA, HIB), Mbuni 007 & 745 (EA), Rauh 577 (EA), Part II Botany 47 (EA).

**F75. Burseraceae**

1 Genus, 3 Species

***Commiphoraeminii* Engl.** Habit: Tree. Habitat: Dry evergreen forest, 600–1000 m. Voucher: SAJIT Z0062 (EA, HIB).

***Commiphorahabessinica* (O. Berg) Engl.** Habit: Shrub. Habitat: Bushed grassland, 500–1900 m. Voucher: Tanaka 234 (EA).

***Commiphoraholtziana* Engl.** Habit: Tree. Habitat: Bushland, 20–1100 m. Voucher: FOKP 11341 (EA, HIB).

**F76. Anacardiaceae**

4 Genera, 10 Species

***Lanneafulva* (Engl.) Engl.** Habit: Shrub. Habitat: Bushland, Wooded grassland, 950–1900 m. Voucher: FOKP 11335 (EA, HIB).

***Lanneaschimperi* (Hochst. ex A. Rich.) Engl.** Habit: Tree. Habitat: Wooded grassland, 1300–1900 m. Voucher: FOKP1818 (EA, HIB).

***Lanneatriphylla* (Hochst. ex A. Rich.) Engl.** Habit: Shrub. Habitat: Wooded grassland, 50–1650 m. Vouchers: FOKP 11674 (EA), Mbuni 674 (EA).

***Ozoroainsignis* Delile** Habit: Tree. Habitat: Wooded grassland, 1–2500 m. Vouchers: SAJIT 004740 (EA, HIB), FOKP 11746 (EA, HIB), Mbuni 746 (EA), Kefri 24 (EA).

**Ozoroainsignissubsp.reticulata (Baker f.) J.B. Gillett** Habit: Tree. Habitat: Wooded grassland, 1500–2500 m. Voucher: Rindsay 169 (EA).

***Rhuslongipes* Engl.** Habit: Tree. Habitat: Wooded grassland, 1000–2400 m. Vouchers: SAJIT 006808 (EA, HIB), FOKP 10908 & 11308 (EA, HIB).

***Searsianatalensis* (Bernh. ex C. Krauss) F.A. Barkley** Habit: Shrub. Habitat: Dry forest wooded grassland, forest margin, forest thicket, 1050–2700 m. Vouchers: FOKP 1096, 11436 & 11290 (EA, HIB), Mbuni 172 (EA).

***Searsiapyroides* (Burch.) Moffett** Habit: Tree. Habitat: Wooded grassland; 1200–2700 m. Vouchers: FOKP 11352 & 11650 (EA, HIB), Mbuni 088 & 650 (EA).

***Searsiatenuinervis* (Engl.) Moffett** Habit: Shrub. Habitat: Wooded grassland, 850–1500 m. Vouchers: FOKP 11740 (EA, HIB), Mbuni 740 (EA).

***Searsiaruspolii* (Engl.) Moffett** Habit: Shrub. Habitat: Evergreen bushland, 1500–2400 m. Vouchers: SAJIT 006871 (EA, HIB), FOKP 1021, 11324 & 11575 (EA, HIB), Mbuni 312 (EA), Kokwaro 2537 (EA).

**F77. Sapindaceae**

8 Genera, 9 Species

***Allophylusabyssinicus* (Hochst.) Radlk.** Habit: Tree. Habitat: Moist forest, river line, 1000–2550 m. Vouchers: SAJIT 006927 (EA, HIB), FOKP 1130, 11359 & 11542 (EA, HIB), Mbuni 279 (EA).

***Cardiospermumgrandiflorum* Sw.** Habit: Climber. Habitat: Upland forest edges, 750–1800 m. Vouchers: Bogdan 3834 (EA), Mabberley & McCall 99 (EA).

***Deinbolliaborbonica* Scheff.** Habit: Tree. Habitat: Secondary bushland, 1200–2550 m. Vouchers: FOKP 1068 (EA, HIB).

***Dodonaeaviscosa* (L.) Jacq.** Habit: Shrub. Habitat: Secondary bushland, 1000–2700 m. Vouchers: SAJIT 004844 (EA, HIB), FOKP 11295 (EA, HIB).

**Dodonaeaviscosasubsp.angustifolia (L. f.) J.G. West** Habit: Shrub. Habitat: Secondary bushland, 1000–2700 m. Vouchers: FOKP 1278 & 11686 (EA, HIB), Mbuni 686 (EA).

***Haplocoelumfoliolosum* (Hiern) Bullock** Habit: Tree. Habitat: Secondary bushland, 900–1800 m. Voucher: SAJIT Z0048 (HIB).

**Lecaniodiscusfraxinifoliussubsp.vaughanii (Dunkley) Friis** Habit: Tree. Habitat: Woodland, 1–1200 m. Voucher: Dale 1093 (EA).

***Lepisanthessenegalensis* (Poir.) Leenh.** Habit: Tree. Habitat: Woodland, riverine forest, 1–1900 m. Voucher: Tweedie 2961 (EA).

***Pappeacapensis* Eckl. & Zeyh.** Habit: Tree. Habitat: Bushland, Wooded grassland, 1050–2300 m. Voucher: Aggundey 5 (EA).

**F78. Rutaceae**

5 Genera, 6 Species

***Clausenaanisata* (Willd.) Hook. f. ex Benth.** Habit: Shrub. Habitat: Secondary bushland, 1–2650 m. Vouchers: FOKP 11460, 11554 & 11657 (EA, HIB), Mbuni 196, 291 & 657 (EA).

***Harrisoniaabyssinica* Oliv.** Habit: Tree. Habitat: Dry bushland, wooded grassland, 1–1650 m. Voucher: Tweedie 3793 (EA).

***Tecleanobilis* Delile** Habit: Tree. Habitat: Wooded grassland, 1050–2550 m. Vouchers: FOKP 11637 (EA, HIB), Mbuni 637 (EA), Lind 5083 (EA).

***Toddaliaasiatica* (L.) Lam.** Habit: Liana. Habitat: Forest margin, grassland thicket, 1200–3000 m. Vouchers: SAJIT 005074 & Z0004 (EA, HIB), FOKP 11401 & 957 (EA, HIB), Nappier 1994 (EA).

***Veprisnobilis* (Delile) Mziray** Habit: Tree. Habitat: Woodland, 1–1400 m. Vouchers: SAJIT 004823 & 005083 (EA, HIB), FOKP 1028 (EA, HIB), Mbuni 194 (EA).

***Veprissimplicifolia* (Engl.) Mziray** Habit: Tree. Habitat: Woodland, 1–1400 m. Voucher: FOKP 1872 (EA, HIB).

**F79. Meliaceae**

2 Genera, 2 Species

***Ekebergiacapensis* Sparrm.** Habit: Tree. Habitat: Dry forest and riverine, 1300–2600 m. Voucher: SAJIT 005079 (EA, HIB).

***Lepidotrichilliavolkensii* (Gurke) Leroy** Habit: Tree. Habitat: Dry forest, Forest margin, 1500–2000 m. Vouchers: FOKP 1068, 11411 & 11623 (EA, HIB), Mbuni 147 & 623 (EA).

**F80. Malvaceae**

10 Genera, 34 Species

***Abutilonbidentatum* Hochst. ex A. Rich.** Habit: Herb. Habitat: Savanna, rocky places, 500–2000 m. Voucher: Lye 9057 (EA).

***Abutilonholstii* K. Schum. ex Engl.** Habit: Shrub. Habitat: Bushland up to 2000 m. Voucher: Webster 8758 (EA).

***Abutilonhirtum* (Lam.) Sweet** Habit: Herb. Habitat: Dry Bushland, 1–1350 m. Voucher: Mas 201 (EA).

***Abutilonlongicuspe* Hochst. ex A. Rich.** Habit: Shrub. Habitat: Upland forest, 1650–3300 m. Voucher: SAJIT 004752 (EA, HIB).

***Abutilonmauritianum* (Jacq.) Medik.** Habit: Shrub. Habitat: Riverine, 0–2300 m. Voucher: Lindsay 2 (EA).

***Dombeyaburgessiae* Gerrard ex Harv.** Habit: Tree. Habitat: Riverine, bushland, Woodland, thickets, 1200–3000 m. Vouchers: SAJIT 004764 (EA, HIB), FOKP 1286 & 11572 (EA, HIB), Mbuni 309 (EA).

***Dombeyatorrida* (J.F. Gmel.) Bamps** Habit: Tree. Habitat: Hagenia forest, 1850–2700 m. Vouchers: FOKP 937 (EA, HIB), Lind et al. 5080 (EA).

***Dombeyaquinqueseta* (Delile) Exell** Habit: Tree. Habitat: Wooded grassland, 1650–1750 m. Voucher: Webster 8757 (EA).

**Dombeyatorridasubsp.erythroleuca (K. Schum.) Seyani** Habit: Tree. Habitat: Secondary bushland, 1850–2700 m. Voucher: FOKP 11363 (EA, HIB).

***Dombeyarotundifolia* (Hochst.) Planch.** Habit. Tree. Habitat: Wooded grassland, 900–2250 m. Voucher: Webster 8757 (EA).

***Hibiscusaponeurus* Sprague & Hutch.** Habit. Shrub. Habitat: Grassland, 600–2600 m. Vouchers: Agnew et al. 10300 (EA), Lindsay 164 (EA).

***Hibiscusberberidifolius* A. Rich.** Habit. Shrub. Habitat: Upper forest edge, 1500–3600 m. Vouchers: SAJIT 007069 (EA, HIB), FOKP 973 (EA, HIB), Mbuni 080 (EA).

***Hibiscusdiversifolius* Jacq.** Habit: Shrub. Habitat: Disturbed places, 1330–2800 m. Voucher: Brodhurst–Hill 8765 (EA).

***Hibiscusfuscus* Garcke** Habit: Shrub. Habitat: Permanent grassland, Disturbed places, 1400–2650 m. Vouchers: FOKP 11291 & 11498 (EA, HIB), Mbuni 234 & 687 (EA), Maas 6358 (EA), Symes 72 (EA).

***Hibiscusludwigii* Eckl. & Zeyh.** Habit. Shrub. Habitat: Cleared forest, Roadside, 1800–3000 m. Vouchers: SAJIT 004472 & 006923 (EA, HIB), FOKP 11504 (EA), Mbuni 059, 117 & 240B (EA), Mabberley & McCall 2003 (EA).

***Hibiscusmicranthus* L. f.** Habit: Shrub. Habitat: Grassland, 1500–2900 m. Vouchers: Agnew et al. 10333 (EA), Bogdan 4769 (EA), Mora 4039 (EA), Mus 97 (EA), Webster 8763 (EA).

***Grewiasimilis* K. Schum.** Habit. Shrub. Habitat: Bushland, Grassland, 700–2250 m. Vouchers: FOKP 1025 & 11667 (EA, HIB), Mbuni 667 (EA), Webster 8752 (EA).

***Grewiatephrodermis* K. Schum.** Habit. Tree. Habitat: Bushland, 700–2250 m. Voucher: Mbuni 226 & 672 (EA), FOKP 11490 & 11672 (EA, HIB).

***Grewiadensa* K. Schum.** Habit. Shrub. Habitat: Bushland, 350–1500 m. Voucher: FOKP 11726 (EA, HIB), Mbuni 726 (EA).

***Kosteletzkyaadoensis* (Hochst. ex A. Rich.) Mast.** Habit: Shrub. Habitat: Grassland, 1200–2650 m. Voucher: FOKP 1294 (EA, HIB).

***Kosteletzkyabegoniifolia* Ulbr.** Habit: Shrub. Habitat: Grassland, 750–2100 m. Habitat: Voucher: FOKP 985 (EA, HIB).

***Malvaverticillata* L.** Habit: Herb. Habitat: Grassland, 1500–3500 m. Voucher: Thulin & Tidings 241 (EA).

***Pavoniaburchellii* (DC.) R.A. Dyer.** Habit. Shrub. Habitat: *Commiphora* Woodland, 100–2300 m. Voucher: Symes 65 (EA).

***Pavoniapropinqua* Garcke** Habit. Shrub. Habitat: *Commiphora* Bushland, 550–1400 m. Voucher: SAJIT 004720 (EA, HIB).

***Pavoniaurens* Cav.** Habit: Shrub. Habitat: Riverine Woodland, 1200–3000 m. Vouchers: FOKP 975 & 11403 (EA, HIB), Mbuni 073 & 103 (EA), Symes 513 (EA), Brown 12809 (EA), Kerfoot 8771 (EA).

***Sidaacuta* Burm. f.** Habit: Herb. Habitat: Grassland, 5–2400 m. Disturbed places on grassland forest, 5–1300 m. Vouchers: Mbuni 245 (EA), FOKP 11509 (EA, HIB).

***Sidaschimperiana* Hochst. ex A. Rich.** Habit: Shrub. Habitat: Dry grassland, bushland forest edges, 1390–2480 m. Voucher: Mbuni 245 (EA).

***Sidaovata* Forssk.** Habit. Herb. Habitat: Dry grassland, 15–2500 m. Voucher: Leippert 5042 (EA).

***Sidarhombifolia*** L. Habit. Herb. Habitat: Grassland, 900–2600 m. Vouchers: SAJIT 004735 (EA, HIB), Napier 1901 (EA), Agnew et al. 10275 (EA).

***Sidaternata* L. f.** Habit. Herb. Habitat: Bamboo forest, montane rain forest, 2000–3280 m. Vouchers: FOKP 11545 (EA, HIB), Mbuni 282 (EA), Symes 100 (EA).

***Sparmanniaricinocarpa* (Eckl. & Zeyh.) Kuntze** Habit: Shrub. Habitat: Forest edge, 2015–3380 m. Vouchers: FOKP 11711 (EA, HIB), Mbuni 711 (EA).

***Triumfettarhomboidea* Jacq.** Habit. Shrub. Habitat: Upland forest, 1100–2200 m. Vouchers: FOKP 11445 (EA, HIB), Mbuni 181 (EA), Symes 215 (EA).

***Triumfettabrachyceras* K. Schum.** Habit. Shrub. Habitat: Forest edge, roadside, 1500–3000 m. Voucher: Hepper & Field 4983 (EA).

***Triumfettacordifolia* A. Rich.** Habit. Shrub. Habitat: Montane forest edges, 1000–2600 m. Vouchers: FOKP 11333 (EA, HIB), Mbuni 069 (EA).

**F81. Thymelaeaceae**

2 Genera, 8 Species

***Englerodaphnesubcordata* (Meisn.) Engl.** Habit: Shrub. Habitat: Dry forest, Bushland, 1450–2500 m. Voucher: SAJIT 005147 (EA, HIB).

***Gnidiachrysantha* (Solms ex Schweinf.) Gilg** Habit: Herb. Habitat: Woodland, 2000–2350 m. Voucher: Symes 382 (EA).

***Gnidiafastigiata* Rendle** Habit: Shrub. Habitat: Subalpine grassland, 2300–3000 m. Vouchers: Dale 3175 (EA), Townsend 2370 (EA).

***Gnidiaglauca* (Fresen.) Gilg** Habit: Tree. Habitat: Forest margin, Bamboo Zone, 2250–3300 m. Vouchers: FOKP 1244 & 11266 (EA), Mbuni 002 (EA), Birnie 480 (EA), Kokwaro 2524 (EA).

***Gnidiakraussiana* Meisn.** Habit: Herb. Habitat: Grassland & rocky hillside, 1650–2650 m. Voucher: Lind 2845 (EA).

***Gnidialatifolia* (Oliv.) Gilg.** Habit: Shrub. Habitat: Bushed grassland, 1–1950 m. Vouchers: SAJIT 006814 (EA, HIB), FOKP 10914 (EA, HIB).

***Gnidialamprantha* Gilg.** Habit: Shrub. Habitat: Woodland, Bushland, 1200–2150 m. Vouchers: FOKP 11267 (EA, HIB), Webster 8740 (EA).

***Struthiolathomsonii* Oliv.** Habit: Shrub. Habitat: Rocky grassland, 2000–3500 m. Vouchers: SAJIT 004793 (EA, HIB), Mbuni 104 (EA), Bogdan 4516 (EA), Wamukoya 128 (EA), Rauh 676 (EA).

**F82. Moringaceae**

1 Genus, 1 Species

***Moringaoleifera* Lam.** Habit: Tree. Habitat: Grassland, Bushland, 15–1350 m. Voucher: Bogdan 4517A (EA).

**F83. Salvadoraceae**

1 Genus, 1 Species

***Salvadorapersica* L.** Habit: Herb. Habitat: Grassland, Bushland, 1–1850 m. Voucher: FOKP 1253 (EA, HIB).

**F84. Resedaceae**

1 Genus, 1 Species

***Cayluseaabyssinica* (Fresen.) Fisch. & C.A. Mey.** Habit: Herb. Habitat: Disturbed, medium altitude, 1100–2800 m. Vouchers: FOKP 11511 (EA, HIB), Mbuni 247 (EA).

**F85. Capparaceae**

4 Genera, 6 Species

***Cleomegynandra* L.** Habit: Herb. Habitat: Dry bushland, 1–2200 m. Voucher: Mabberley & McCall 100 (EA).

***Cadabafarinosa* Forssk.** Habit: Shrub. Habitat: Bushland, 1–1900 m. Vouchers: FOKP 1254 & 11338 (EA, HIB).

***Cappariserythrocarpos* Isert.** Habit: Shrub. Habitat: Bushed or wooded grassland, 1100–1800 m. Voucher: Tweedie 1931 (EA).

***Maeruadecumbens* (Brongn.) DeWolf** Habit: Shrub. Habitat: Bushland, 1–1800 m. Voucher: FOKP 1248 (EA, HIB).

***Maeruaparvifolia* Pax** Habit: Shrub. Habitat: Bushland, 700–1650 m. Voucher: Tweedie 1571 (EA).

***Maeruatriphylla* A. Rich.** Habit: Shrub. Habitat: Bushland, 1–2300 m. Vouchers: SAJIT Z0060 (HIB), FOKP 11304 & 11332 (EA, HIB).

**F86. Brassicaceae**

4 Genera, 4 Species

***Arabisalpina* L**. Habit: Herb. Habitat: Highland grassland, 2800–3500 m. Voucher: Mabberley & McCall 211 (EA).

***Cardamineafricana* L.** Habit: Herb. Habitat: Highland grassland, 2200–3300 m. Vouchers: FOKP 1723 & 11615 (EA, HIB), Mbuni 615 (EA).

***Nasturtiumofficinale* R. Br.** Habit: Herb. Habitat: Highland streamside, 1500–2700 m. Voucher: FOKP 1731 (EA, HIB).

***Thlaspialliaceum* L**. Habit: Herb. Habitat: Bamboo forest, 3000–3500 m. Voucher: Mabberley & McCall 258 (EA).

**F87. Santalaceae**

4 Genera, 5 Species

***Osyridicarposschimperianus* (Hochst. ex A. Rich.) A. DC.** Habit: Shrub. Habitat: Rocky site, thickets Bushland, Grassland, 1550–2415 m. Vouchers: Mbuni 750 (EA), FOKP 11750 (EA).

***Osyrislanceolata* Hochst. & Steud.** Habit: Shrub. Habitat: Rocky site, forest thicket, bushland, grassland, 900–2550 m. Vouchers: SAJIT 004835 (EA, HIB), FOKP 1093, 1094, 11269 & 11270 (EA, HIB), Mbuni 006 (EA).

***Thesiumradicans* Hochst. ex A. Rich.** Habit: Herb. Habitat: Grassland, 1800–3000 m. Voucher: SAJIT 006858 (EA, HIB).

***Thesiumussanguense* Engl.** Habit: Herb. Habitat: Grassland, around 2100 m. Voucher: Friis & Hansen 2508 (EA).

***Viscumtuberculatum* A. Rich.** Habit: Parasitic herb. Habitat: Dry woodland forest, 1650–2400 m. Vouchers: SAJIT 006852 & 007078 (EA, HIB), FOKP 1106 & 10952 (EA, HIB), Mbuni 727 (EA).

**F88. Loranthaceae**

6 Genera, 11 Species

***Englerinawoodfordioides* (Schweinf.) Balle.** Habit: Shrub. Habitat: Montane forest, 1600–3050 m. Vouchers: SAJIT 006885 & 006905 (EA, HIB), FOKP 1029 & 11631 (EA, HIB), Mbuni 631 (EA), Bogdan 4512 (EA), Mabberley 33 (EA).

***Erianthemumdregei* (Eckl. & Zeyh.) Tiegh.** Habit: Shrub. Habitat: Bushland, 0–2950 m. Vouchers: SAJIT 004846 & 004847 (EA, HIB), FOKP 1103 & 11329 (EA, HIB).

***Oncocalyxfischeri* (Engl.) M.G. Gilbert** Habit: Shrub. Habitat: Bushland, 550–2100 m. Vouchers: Lucas 175 (EA), Kazuaki 28 (EA).

***Oncocalyxsulfureus* (Engl.) Wiens & Polhill** Habit: Shrub. Habitat: Dry upland forest, Bushland, 550–2680 m. Voucher: SAJIT Z0024 (EA, HIB).

***Oncocalyxugogensis* (Engl.) Wiens & Polhill** Habit: Shrub. Habitat: Dry Bushland 10–1425 m. Voucher: Carter & Stannard 79 (EA).

***Phragmantheradschallensis* (Engl.) M.G. Gilbert.** Habit: Shrub. Habitat: Wooded grassland, 1400–1900 m. Vouchers: SAJIT 006865 (EA, HIB), FOKP 11373 (EA, HIB), Mbuni 109 (EA), Wiens 4504 (EA).

***Phragmantherausuiensis* (Oliv.) M.G. Gilbert.** Habit: Shrub. Habitat: Montane forest, 1150–2600 m. Vouchers: SAJIT 004841 (EA, HIB), FOKP 11323 (EA, HIB).

***Plicosepaluscurviflorus* (Benth. ex Oliv.) Tiegh.** Habit: Shrub. Habitat: Wooded grassland, 500–2320 m. Vouchers: FOKP 11340 & 1258 (EA, HIB), Webster 8813 (EA).

***Plicosepalusmeridianus* (Danser) Wiens & Polhill** Habit: Shrub. Habitat: Dry bushland, 100–1550 m. Vouchers: Tweedie 2974 (EA), Archer 494 (EA).

***Plicosepalussagittifolius* (Engl.) Danser** Habit: Shrub. Habitat: Dry bushland & wooded grassland, 150–2100 m. Voucher: Lucas 186 (EA) .

***Tapinanthusbuvumae* (Rendle) Danser** Habit: Climber Habitat: Wooded grassland, up to 1980 m. Vouchers: SAJIT 004831 & 005060 (EA, HIB).

**F89. Plumbaginaceae**

1 Genus, 1 Species

***Plumbagozeylanica* L.** Habit: Shrub. Habitat: Dry bushland, 700–1860 m. Voucher: Mabberley 87 (EA).

**F90. Polygonaceae**

3 Genera, 9 Species

***Harpagocarpussnowdenii* Hutch. & Dandy** Habit: Herb. Habitat: Highland forest up to 1650 m. Vouchers: FOKP 11541 (EA, HIB), Mbuni 278 (EA).

***Persicariadecipiens* (R. Br.) K.L. Wilson** Habit: Herb. Habitat: Waterside grassland, 1000–3170 m. Vouchers: FOKP 978 & 1129 (EA, HIB).

***Persicarianepalensis* (Meisn.) Miyabe** Habit: Shrub. Habitat: Wet forest zones, 2250–3500 m. Vouchers: SAJIT 004740 (EAHIB), Mbuni 043 (EA), Ivens 1250 (EA).

***Persicariasenegalensis* (Meisn.) Soják** Habit: Herb. Habitat: Waterside grassland up to 2850 m. Vouchers: FOKP 11730 (EA, HIB), Mbuni 730(EA).

***Persicariasetosula* (A. Rich.) K.L. Wilson** Habit: Herb. Habitat: Higher altitudes in waterside, 1330–3000 m. Vouchers: FOKP 1734, 11307 & 11357 (EA, HIB), Mbuni 043 & 093 (EA).

***Persicariastrigosa* (R. Br.) Nakai** Habit: Herb. Habitat: Grassland, 1600–3000 m. Voucher: Ivens 1250 (EA).

***Rumexabyssinicus* Jacq.** Habit: Herb. Habitat: Upper forest levels, 1600–2800 m. Voucher: Townsend 2385 (EA).

***Rumexsteudelii* Hochst. ex A. Rich.** Habit: Herb. Habitat: Upper forest, streamside, around 1200 m. Vouchers: FOKP 1203 & 1225 (EA, HIB), Mbuni 089 (EA, HIB).

***Rumexusambarensis* (Dammer) Dammer.** Habit: Herb. Habitat: Secondary shrubland, 900–2300 m. Vouchers: FOKP 11565 (EA, HIB), Mbuni 302 (EA).

**F91. Caryophyllaceae**

4 Genera, 7 Species

***Cerastiumafromontana* T.C.E. Fr.** Habit: Herb. Habitat: Grassland, 2300–3500 m. Voucher: Symes 648 (EA).

***Cerastiumlanceolatum* (Poir.) Volponi** Habit: Herb. Habitat: Wet Grassland, 1500–3500 m. Vouchers: SAJIT 006860 (EA, HIB), FOKP 1063 (EA, HIB).

***Cerastiumoctandrum* Hochst. ex A. Rich.** Habit: Herb. Habitat: Grassland, 2500–3500 m. Voucher: Knight 95 (EA).

***Lychniscrassifolia* (T.C.E. Fr.) M. Popp** Habit: Herb. Habitat: Grassland, 3150– 3350 m. Voucher: Thulin & Tidigs 232 & 3150 (EA).

***Silenegallica* L.** Habit: Herb. Habitat: Alpine zone, 1900–3500 m. Vouchers: FOKP 1801 & 11306 (EA, HIB), Mbuni 042 (EA), Symes 125 (EA).

***Stellariamannii* Hook. f.** Habit: Herb. Habitat: Wet highland forest, 1550–2500 m. Vouchers: FOKP 1121 & 1224 (EA, HIB).

***Stellariasennii* Chiov.** Habit: Herb. Habitat: Wet highland forest, 2020–3500 m. Vouchers: Mabberley & McCall 203 (EA), Thulin & Tidigs 238 (EA).

**F92. Amaranthaceae**

7 Genera, 11 Species.

***Achyranthesaspera* L**. Habit: Herb. Habitat: Woodland, disturbed dry places, 0–3080 m. Vouchers: Mbuni 81 (EA), FOKP 11345 (EA, HIB).

**Achyranthesasperavar.sicula L.** Habit: Herb. Habitat: Disturbed dry places, 0–3080 m. Voucher: Part II Botany 15 (EA)

***Aervalanata* (L.) Juss.** Habit: Shrub. Habitat: Open ground, 1–2200 m. Vouchers: FOKP 11485 (EA, HIB), Mbuni 221 (EA).

***Amaranthushybridus* L.** Habit: Herb. Habitat: Disturbed ground, 1500–2600 m. Vouchers: FOKP 11516 (EA, HIB), Mbuni 252 (EA).

***Amaranthusspinosus* L.** Habit: Herb Habitat: Wooded grassland, 0–2000 m. Vouchers: FOKP 11486 (EA, HIB), Mbuni 222 (EA).

***Celosiaanthelminthica* Asch**. Habit: Climber. Habitat: Dry bushland 500–2300 m. Voucher: Mabberley & McCall 107 (EA).

***Chenopodiumopulifolium* Schrad. ex W.D.J. Koch & Ziz** Habit: Herb. Habitat: Cultivated land, roadside, waste places, 1600–2300 m. Vouchers: FOKP 11342 (EA, HIB), Mbuni 078 (EA).

***Cyathulacylindrica*** M**oq.** Habit: Herb. Habitat: Rocky scarps with bushland, 1300–3240 m. Voucher: SAJIT 007115 (EA, HIB).

***Cyathulapolycephala* Baker** Habit: Herb. Habitat: Grassland, woodland, 1600–3000 m. Voucher: FOKP 1126 (EA, HIB).

***Cyathulauncinulata* (Schrad.) Schinz.** Habit: Herb. Habitat: Forest edges, 1500–2900 m. Vouchers: FOKP 1126 (EA, HIB), Part II Botany 26 (EA).

***Pupalialappacea* (L.) Juss.** Habit: Herb. Habitat: Dry bushland, 10–2060 m. Vouchers: FOKP 11693 & 11721 (EA, HIB), Mbuni 693 & 721 (EA).

**F93. Phytolaccaceae**

1 Genus, 2 Species

***Phytolaccadodecandra* L’Hér.** Habit: Shrub. Habitat: Moist forest margin, Riverine forest, 1650–2450 m. Vouchers: SAJIT 004749 & 006884 (EA, HIB), FOKP 969 & 1038 (EA, HIB).

***Phytolaccaoctandra* L.** Habit: Shrub. Habitat: Moist forest margin, 1500–2400 m. Vouchers: SAJIT 004776, 005085 (EA, HIB), FOKP 1111 & 11327 (EA, HIB), Mbuni 063 (EA).

**F94. Nyctaginaceae**

1 Genus, 1 Species

***Boerhaviaplumbaginea* Cav.** Habit: Herb. Habitat: Grassland, 600–2030 m. Voucher: FOKP 1250 (EA, HIB).

**F95. Molluginaceae**

1 Genus, 1 Species

***Corrigiolalitoralis* L.** Habit: Herb. Habitat: Mountain grassland roadside, 2350–3500 m. Voucher: Mabberley & McCall 220 (EA).

**F96. Basellaceae**

1 Genus, 1 Species

***Basellaalba* L.** Habit: Shrub. Habitat: Riverine forest margin and hedges, 1500–3000 m. Vouchers: FOKP 982 & 11355 (EA, HIB), Mbuni 091 (EA).

**F97. Talinaceae**

1 Genus, 2 Species

***Talinumcaffrum* (Thunb.) Eckl. & Zeyh.** Habit: Herb. Habitat: Bushland, 1000–2000 m. Voucher: Greenway 9890 (EA).

***Talinumarnotii* Hook. f.** Habit: Herb. Habitat: Grassland, 800–2000 m. Voucher: Carter & Stannard 62 (EA).

**F98. Cactaceae**

1 Genus, 1 Species

***Rhipsalisbaccifera* (J.S. Muell.) Stearn** Habit: Epiphyte. Habitat: Dry forest, 150–2300 m. Voucher: FOKP 988 (EA, HIB).

**F99. Cornaceae**

1 Genus, 1 Species

***Cornusvolkensii* Harms** Habit: Tree. Habitat: Bamboo zone, wet upland, 2100–3200 m. Vouchers: SAJIT 006804 & 007123 (EA, HIB), FOKP 1058, 11445, 10904 & 11544 (EA, HIB), Mbuni 181 & 281 (EA).

**F100. Balsaminaceae**

1 Genus, 9 Species

***Impatienselegantissima* Gilg** Habit: Herb. Habitat: Streamsides in wet forest, 1000–2750 m. Vouchers: Hepper & Field 5017, Maas 4774 (EA), Webster 8737 (EA).

***Impatienshochstetteri* Warb.** Habit: Herb. Habitat: Streamsides in wet forest, 1000–2750 m. Vouchers: SAJIT 004858, 005097 & Z0014 (EA, HIB), FOKP 1303 & 11382 (EA, HIB), Knox 3355 (EA), Mabberley 485 (EA).

***Impatienshoehnelii* T.C.E. Fr.** Habit: Herb. Habitat: Upper forest level, 1500–3470 m. Vouchers: SAJIT 006810 (EA, HIB), FOKP 10910 (EA, HIB), Hughes 2 (EA).

***Impatiensirvingii* Hook. f.** Habit: Herb. Habitat: Wet forest floor up to 2000 m. Voucher: Webster 8738 (EA).

***Impatiensmeruensis* Gilg.** Habit: Herb. Habitat: Highland forest, marshes and streamside, 1000–3550 m. Vouchers: SAJIT 004780 (EA, HIB), FOKP 1720, 11385 & 11528 (EA, HIB), Mbuni 120 & 264 (EA), Lind 2828 (EA).

**Impatiensmeruensissubsp.cruciata (T.C.E. Fr.) Grey–Wilson.** Habit: Herb. Habitat: Highland forest, marshes & streamside, 1000–3550 m. Vouchers: SAJIT 005089 (EA, HIB), FOKP 11643 (EA, HIB), Mbuni 643 (EA), Lind 2828 (EA), Knox 3356 (EA).

***Impatienspseudoviola* Gilg** Habit: Herb. Habitat: Lower Highland forest, 1500–3300 m. Voucher: Hughes 1 (EA).

***Impatienssodenii* Engl. & Warb.** Habit: Herb. Habitat: Escarpment zones with frequent mist, 1000–2700 m. Vouchers: SAJIT 004733 (EA, HIB).

***Impatienstinctoria* A. Rich** Habit: Herb. Habitat: Highland forest areas, streamside, 1800–3550 m. Vouchers: SAJIT 004789 & 006819 (EA, HIB), FOKP 10919, 11356 &11644 (EA, HIB), Webster 8737 (EA), Mbuni 092 & 644 (EA).

**F101. Sapotaceae**

2 Genera, 2 Species

**Chrysophyllumoliviformesubsp.angustifolium (Lam.) T.D. Penn.** Habit: Tree. Habitat: Moist forest, 1500–2200 m. Voucher: FOKP 1067 (EA, HIB).

***Pouteria adolfi–friedericii* (Engl.) A. Meeuse** Habit: Tree. Habitat: Moist forest, 1500–2400 m. Vouchers: SAJIT 006904 (EA, HIB), FOKP 11004 (EA, HIB), Colby H134 (EA).

**F102. Ebernaceae**

2 Genera, 3 Species

***Diospyrosscabra* (Chiov.) Cufod.** Habit: Tree. Habitat: Rocky hillsides, 400–1050 m. Voucher: FOKP 11331 (EA, HIB).

***Eucleadivinorum* Hiern** Habit: Shrub. Habitat: Bushed grassland, 1–2700 m. Vouchers: FOKP 1099, 1107 & 11461 (EA, HIB), Mbuni 197 & 743 (EA).

***Euclearacemosa* L.** Habit: Tree. Habitat: Bushland, 1–2250 m. Vouchers: FOKP 11659 (EA, HIB), Mbuni 659 (EA).

**F103. Primulaceae**

4 Genera, 5 Species

***Anagallishexamera* P. Taylor**. Habit: Herb. Habitat: streamside marshes, 2275–2600 m. Voucher: Hale 209 (EA).

***AnagallisSerpens* subsp. *meyeri-johannis* (Endl.) P. Taylor** Habit: Herb. Habitat: Upland dry forest, 2600–3550 m. Vouchers: FOKP 988 (EA, HIB), Thulin & Tidigs 221 (EA).

***Maesalanceolata* Forssk.** Habit: Shrub. Habitat: Widespread secondary forest, Forest margin, 1300–2853 m. Vouchers: SAJIT 004770 & 005138 (EA, HIB), FOKP 1033 (EA, HIB).

***Myrsineafricana* L.** Habit: Shrub. Habitat: Upland dry forest, 1500–3000 m. Vouchers: SAJIT 004801 (EA, HIB), FOKP 11385 (EA, HIB), Mbuni 009 & 757 (EA), Hepper & Field 5027 (EA), Lind & Agnew 5160 (EA).

***Rapaneamelanophloeos* (L.) Mez.** Habit: Tree. Habitat: Upland forest, to the edge of moorland, 1200–3550 m. Vouchers: SAJIT 006806 (EA), FOKP 10906 & 11267 (EA, HIB), Mbuni 003 (EA), Knight 87 (EA).

**F104. Ericaceae**

2 Genera, 6 Species

***Agaristasalicifolia* (Lam.) G. Don** Habit: Tree. Habitat: Secondary forest, high altitude bushland, forest edge, 2100–3300 m. Vouchers: SAJIT 004758 (EA, HIB), FOKP 1086 & 1717 (EA, HIB), Friis & Hansen 2513 (EA), Thulin & Tidigs 257 (A).

***Ericaarborea* L.** Habit: Shrub. Habitat: Heath, woodland, Rocky, bushland, 2300–2700 m. Vouchers: SAJIT 004801 (EA, HIB), Thulin & Tidigs 92 (EA).

***Ericafilago* (Alm & T.C.E. Fr.) Beentje** Habit: Shrub. Habitat: Heath zones, 2500–3550 m. Voucher: Thulin & Tidings 257 (EA).

***Ericasilvatica* (Welw. ex Engl.) Beentje** Habit: Shrub. Habitat: Moorlands, alpine zones, 2300–3550 m. Voucher: Bogdan 4984 (EA).

***Ericaventricosa* Thunb.** Habit: Shrub. Habitat: Boggy moorlands, heath zones, 2500–3550 m. Voucher: SAJIT 004807 (EA, HIB).

***Ericawhyteana* Britten** Habit: Shrub. Habitat: Wooded grassland, 2700–3550 m. Vouchers: Thulin & Tidings 89 (EA), Bogdan 4999 (EA).

**F105. Rubiaceae**

25 Genera, Species 64

***Agathisanthemumglobosum* (Hochst. ex A. Rich.) Klotzsch** Habit: Herb. Habitat: Wooded grassland, 1600–2170 m. Vouchers: Lewis 5987 (EA), Blake 2132 (EA).

***Anthospermumusambarense* K. Schum.** Habit: Shrub. Habitat: Ericaceous belt, alpine bushland, 2150–3350 m. Voucher: Thulin &Tidigs 251 (EA).

***Anthospermumherbaceum* L. f.** Habit: Herb. Habitat: Forest edge, wet forest zone, 1560–2650 m. Vouchers: Mbuni 029 (EA. HIB), Symes 211 (EA).

***Canthiumpseudosetiflorum*Bridson** Habit: Shrub. Habitat: Evergreen bushland, dry woodland, 750–1750 m. Voucher: Bridson 1079 (EA).

***Coffeaeugenioides* S. Moore** Habit: Shrub. Habitat: Dry bushland, 1450–2250 m. Voucher: Thomas 232 (EA).

***Conostomiumquadrangulare* (Rendle) Cufod**. Habit: Herb. Habitat: Rocky bushland, 60–1950 m. Voucher: Napier 2067 (EA).

***Fadogiacienkowskii* Schweinf**. Habit: Shrub. Habitat: Wooded grassland, 1800–1950 m. Voucher: Napier 2005 (EA).

***Fagaropsisangolensis* (Engl.) H.M. Gardner** Habit: Tree. Habitat: Dry Forest, 1150–2100 m. Voucher: Lindsay 16 (EA).

***Galinieracoffeoides* Delile** Habit: Shrub. Habitat: Moist forest, 1850–2400 m. Vouchers: Friis & Hansen 2552 (EA), Dale 907(EA).

***Galinierasaxifraga* (Hochst.) Bridson** Habit: Shrub. Habitat: Moist forest, 1850–2400 m. Vouchers: SAJIT 005072 & 006795 (EA, HIB), FOKP 936, 1074, 10895, 11361 & 11412 (EA, HIB), Mbuni 148 (EA).

***Galiumaparine* L.** Habit: Climber. Habitat: Grassland, 1600–3200 m. Voucher: SAJIT 006856 (EA, HIB).

***Galiumaparinoides* Forssk.** Habit: Climber. Habitat: Montane forest edges, 1600–3500 m. Vouchers: FOKP 11404 (EA, HIB), Mbuni 030 (EA), Verdcourt & Taylor et al. 2434 (EA).

***Galiumscioanum* Chiov.** Habit: Herb. Habitat: Moist woodland, 1800–2700 m. Vouchers: FOKP 1138 & 11536 (EA, HIB), Mbuni 272 (EA), Thulin & Tidings 175 (EA), Tweedie 2699 (EA).

***Galiumkenyanum* Verdc**. Habit: Herb. Habitat: Alpine grassland, 2880–3540 m. Voucher: Tweedie 3864 (EA), Mabberley 51 (EA).

***Galiumruwenzoriense* (Cortesi) Ehrend.** Habit: Herb. Habitat: Forest edges & bushland, 2700–3500 m. Vouchers: Knox 3387 (EA), Thulin & Tidigs 101 (EA).

***Galiumthunbergianum* Eckl. & Zeyh**. Habit: Herb. Habitat: Montane forest, 2050–3550 m. Vouchers: SAJIT 006857 (EA, HIB), FOKP 1060 (EA, HIB), Knox 3376 (EA).

***Gardeniaternifolia* Schumach. & Thonn.** Habit: Tree. Habitat: Wooded grassland, riverine, 1100–2500 m. Voucher: Champion 189 (EA).

***Gardeniavolkensii* K. Schum.** Habit: Shrub. Habitat: Riverine woodland, wooded grassland, 1–1750 m. Vouchers: SAJIT 006878 (EA, HIB), FOKP 11310 (EA, HIB), Rindsay 1958 (EA).

***Keetiagueinzii* (Sond.) Bridson.** Habit: Climber. Habitat: Moist forest, bushland, riverine, 1300–2500 m. Vouchers: SAJIT 004819 & 005066 (EA, HIB), FOKP 1017 (EA, HIB), Mbuni 052 (EA, HIB), Mabberley 155 (EA), Lind 2849 (EA), Friis & Hansen 2545 (EA).

***Kohautiacoccinea* Royle** Habit: Shrub. Habitat: Riverine woodland, rocky bushland, dry grassland, 1250–2500 m. Vouchers: Faden 144 (EA), Symes 208 (EA).

***Meynatetraphylla* (Schweinf. ex Hiern) Robyns.** Habit: Shrub. Habitat: Riverine woodland, rocky bushland, 1–1400 m. Voucher: Faden 77 & 792 (EA).

***Mitracarpusvillosus* (Sw.) DC.** Habit: Herb. Habitat: As weed on cultivated ground, 60–1400 m. Voucher: SAJIT Z0008 (HIB).

**Oldenlandiacorymbosavar.nana (Bremek.) Verdc.** Habit: Herb. Habitat: Dry areas, 1–2500 m. Voucher: Symes 754 (EA).

**Oldenlandiacorymbosavar.caespitosa (Benth).** Habit: Herb. Habitat: Dry areas, 1–2500 m. Voucher: Verdcourt 736 (EA).

***Oldenlandiaherbacea* (L.) Roxb.** Habit: Herb. Habitat: Dry grassland, 75–2400 m. Vouchers: FOKP 11315 (EA, HIB), Mbuni 051 (EA).

***Oldenlandialancifolia* (Schumach.) DC.** Habit: Herb. Habitat: Wooded grassland, 400–2350 m. Voucher: SAJIT 007055 (EA, HIB).

***Oldenlandiamonanthos* (Hochst. ex A. Rich.) Hiern**: Habit: Herb. Habitat: Montane grassland, 1650–3150 m. Vouchers: SAJIT 006820 (EA, HIB), FOKP 10920 (EA, HIB).

***Oxyanthusspeciosus* DC.** Habit: Tree. Habitat: Forest riverine, 1400–2200 m. Vouchers: SAJIT 006898 (EA, HIB), FOKP 1275, 1736 & 11463 (EA, HIB), Mbuni 199 (EA).

**Oxyanthusspeciosussubsp.stenocarpus (K. Schum.) Bridson** Habit: Tree. Habitat: Riverine forest, 1400–2200 m. Voucher: SAJIT 007095 (EA, HIB).

***Pavettaabyssinica* Fresen.** Habit: Shrub. Habitat: Forest riverine, secondary bushland, 1500–2550 m. Vouchers: SAJIT 005069 (EA, HIB), FOKP 1084, 1719 & 11607 (EA, HIB), Mbuni 607 (EA).

***Pavettacrassipes* K. Sch.** Habit: Shrub. Habitat: Wooded grassland, 900–1500 m. Voucher: Nyamwaya 28 (EA).

***Pavettadolichantha* Bremek.** Habit: Shrub. Habitat: Bushland and rocky slopes, 750–1650 m. Voucher: Tweedie 3029 (EA).

***Pavettaelliottii* K. Schum. & K. Krause.** Habit: Shrub. Habitat: Bushland, grassland, forest margin, 1400–1900 m. Voucher: Pole et al. 1512 (EA).

***Pavettagardeniifolia* Hochst. ex A. Rich.** Habit: Shrub. Habitat: Rocky, bushland, 7005–2500 m. Vouchers: FOKP 11429 (EA, HIB), Mbuni 165 (EA).

***Pentasdecora* S. Moore.** Habit: Herb. Habitat: Wooded grassland, 1590–2700 m. Voucher: Lucas 200 (EA).

***Pentaslanceolata* (Forssk.) Deflers.** Habit: Shrub. Habitat: Forest edge, 1200–2830 m. Voucher: Paswa 49 (EA).

**Pentaslanceolatavar.leucaster (K. Krause) Verdc.** Habit: Herb. Habitat: Forest edges, 1200–2830 m. Vouchers: Napier 1902 (EA), Lind 5093 (EA).

***Pentaslongiflora* Oliv.** Habit: Herb. Habitat: Bushed grassland, 500–2500 m. Vouchers: FOKP 1095, 11299 & 11621 (EA, HIB), Mbuni 621 (EA).

***Pentasparvifolia* Hiern.** Habit: Shrub. Habitat: Bushed grassland, 650–2400 m. Vouchers: FOKP 11467 (EA, HIB), Mbuni 203 (EA).

***Pentaspubiflora* S. Moore** Habit: Shrub. Habitat: Montane and riverine forest, 1800–2400 m. Vouchers: FOKP 11290 (EA, HIB), Mbuni 026 (EA).

***Pentaszanzibarica* (Klotzsch) Vatke** Habit: Shrub. Habitat: Riverine forest, 1800–2440 m. Vouchers: FOKP 11620 (EA, HIB), Mbuni 620 (EA).

***Pentasschimperiana* Vatke** Habit: Herb. Habitat: Forest clearing, 2100–2700 m. Vouchers: SAJIT 004741 (EA, HIB), FOKP 1008 (EA, HIB), Tweedie 2801 (EA), Gardener 2852 (EA).

***Pentanisiaouranogyne* S. Moore**. Habit: Herb. Habitat: Grassland, 300–2400 m. Voucher: Thomasson & Hansson 91A (EA).

***Pentanisiaschweinfurthii* Hiern.** Habit: Herb. Habitat: Wooded grassland, 1500–2480 m. Voucher: Symes 278 (EA).

***Psychotriafractinervata* E.M.A. Petit** Habit: Shrub. Habitat: *Ocotea* forest, 1900–2550 m. Voucher: FOKP 1873 (EA, HIB).

***Psychotriakirkii* Hiern.** Habit: Shrub. Habitat: Bushland, Wooded grassland, 250–2250 m. Vouchers: FOKP 11298 & 11437 (EA, HIB), Mbuni 173 (EA).

**Psychotriamahoniivar.puberula (E.M.A. Petit) Verdc**. Habit: Tree. Habitat: Swampy forest, riverine, 1400–2400 m. Voucher: Gardener 1398a (EA).

**Psychotriamahoniivar.pubescens (Robyns) Verdc.** Habit: Tree. Habitat: Swampy forest, riverine, 1400–2400 m. Voucher: Tweedie 2801 (EA).

***Pyrexschimperiana* (A. Rich.) Bridson** Habit: Tree. Habitat: Riverine, 1400–2400 m. Vouchers: FOKP 11474 (EA, HIB), Mbuni 210 (EA).

***Richardiabrasiliensis* Gomes** Habit: Herb. Habitat: Grassland, 1500–2010 m. Voucher: FOKP 11489 (EA, HIB), Mbuni 225 (EA).

***Rubiacordifolia* L.** Habit: Herb. Habitat: Woodland clearings, riverine bushland, 1240–3120 m. Voucher: Mungai 84 & 128 (EA).

***Rutideaorientalis*Bridson** Habit: Shrub. Habitat: Woodland, 1400–2400 m. Vouchers: SAJIT 006891 (EA, HIB), FOKP 10991 (EA, HIB).

***Rytigynianeglecta* (Hiern) Robyns** Habit: Tree. Habitat: Riverine forest, 1350–2200 m. Vouchers: FOKP 1809 (EA, HIB), Paswa 54 (EA).

***Rytigyniauhligii* (K. Schum. & K. Krause) Verdc.** Habit: Tree. Habitat: Wet forest, 1050–2400 m. Vouchers: FOKP 11404, 11452 & 11603 (EA, HIB), Mbuni 140, 188 & 603 (EA, HIB).

***Spermacoceminutiflora* (K. Schum.) Verdc.** Habit: Herb. Habitat: Woodland, grassland, 1500–2350 m. Voucher: Napier 1973 (EA).

***Spermacoceprinceae* (K. Schum.) Verdc.** Habit: Herb. Habitat: Wet forest, 1470–2650 m. Vouchers: SAJIT 006926 (EA, HIB), FOKP 1129 (EA, HIB).

***Spermacocesphaerostigma* (A. Rich.)** Habit: Herb. Habitat: Grassland, 900–2200 m. Voucher: Symes 198 & 756 (EA).

***Coptospermagraveolens* (S. Moore) Bremek.** Habit: Tree. Habitat: Bushland, forest thicket, 1–2500 m. Vouchers: FOKP 11429 (EA, HIB), Mbuni 165 (EA).

***Coptospermapavettoides* (Harv.) Sim** Habit: Shrub. Habitat: Secondary bushland, 1200–1750 m. Voucher: FOKP 1009 (EA, HIB).

***Vangueriaapiculata* K. Schum.** Habit: Tree. Habitat: Thickets, riverine, wooded grassland, bushland, 950–2300 m. Vouchers: SAJIT 004742 & 005061 (EA, HIB), Nyamwaya 31 (EA).

***Vangueriainfausta* Burch.** Habit: Shrub. Habitat: Thickets, riverine, wooded grassland, bushland, 1–2100 m. Vouchers: SAJIT 006853 (EA, HIB), FOKP 10953 (EA, HIB).

***Vanguerialinearisepala* K. Schum.** Habit: Shrub. Habitat: Wooded grassland, bushland, 750–2100 m. Voucher: Friis & Hansen 2539 (EA).

***Vangueriamadagascariensis* J.F. Gmel.** Habit: Tree. Habitat: Riverine, wooded grassland, bushland, 750–2100 m. Vouchers: Mbuni 751 (EA), Maundu & Musya 31 (EA).

***Vangueriavolkensii* K. Schum**. Habit: Tree. Habitat: Riverine, bushland, 900–2300 m. Vouchers: FOKP 956, 1023, 1075 & 11405 (EA, HIB), Mbuni 141, 738 (EA), Bridson 105 (EA).

**F106. Gentianaceae**

3 Genera, 11 Species

***Chironiaelgonensis* Bullock**. Habit: Herb. Habitat: Swamps, 1705–2325 m. Voucher: Symes 1406 (EA).

***Sebaeabrachyphylla* Griseb**. Habit: Herb. Habitat: Wooded grassland, 1650–3470 m. Vouchers: Thulin 218 (EA), Verdcourt 2439 (EA).

***Sebaeagrandis* (E. Mey.) Steud.** Habit: Herb. Habitat: Wooded grassland on shallow soil, 1550–2160 m. Voucher: Symes 1010 (EA).

***Swertiaabyssinica* Hochst.** Habit: Herb. Habitat: Alpine zone, 2000–3300 m. Vouchers: Mabberley 580 (EA), Nemomissa 970830 (EA).

***Swertiabrownii* J. Shah.** Habit: Herb. Habitat: Wooded grassland, 1800–2600 m. Vouchers: SAJIT 006925 (EA, HIB), FOKP 1128 (EA, HIB).

***Swertiacrassiuscula* Gilg** Habit: Herb. Habitat: Wet stony, heath, lower alpine zones, 2700–3500 m. Vouchers: Dale 3263 & 3264 (EA).

***Swertiakilimandscharica* Engl.** Habit: Herb. Habitat: Montane, subalpine, 2100–3550 m. Vouchers: FOKP 11282 (EA), Mbuni 018 (EA), Mabberley & McCall 124 (EA), Lucas 169 (EA), Mainwaring 46 (EA).

***Swertialugardiae* Bullock** Habit: Herb. Habitat: Montane and subalpine grassland, 2100–3500 m. Vouchers: Sileshi 4 & 5 (EA).

***Swertiatetrandra* Hochst.** Habit: Herb. Habitat: Disturbed upland grassland, 1800–2200 m. Voucher: SAJIT 005101 (EA, HIB).

***Swertiausambarensis* Engl.** Habit: Herb. Habitat: Montane grassland, 1600–3500 m. Vouchers: Mabberley & McCall 264 (EA), Thulin & Tidigs 39 (EA), Symes 623 (EA).

***Swertiawelwitschii* Engl.** Habit: Herb. Habitat: Roadside, 1845–2400 m. Voucher: Jex–Blake 2122 (EA).

**F107. Loganiaceae**

1 Genus, 2 Species

***Strychnoshenningsii* Gilg.** Habit: Tree. Habitat: Forest thicket on rocky hills, 850–2100 m. Vouchers: SAJIT Z0035 (HIB), FOKP 11661 (EA), Mbuni 661(EA).

***Strychnosmitis* S. Moore** Habit: Tree. Habitat: Riverine forest, 1–1950 m. Vouchers: FOKP 11464 (EA), Mbuni 200 (EA).

**F108. Apocynaceae**

20 Genera, 37 Species

***Acokantheraoppositifolia* (Lam.) Codd** Habit: Shrub. Habitat: Woodland, 1450–2100 m. Voucher: FOKP 1097 (EA, HIB).

***Acokantheraschimperi* (A. DC.) Schweinf.** Habit: Tree. Habitat: Wooded grassland, 1200–2300 m. Voucher: FOKP 11306 (EA, HIB).

***Carallumaarachnoidea* (P.R.O. Bally) M.G. Gilbert.** Habit: Herb. Habitat: Rocky grassland, 100–1300 m. Voucher: Hartmann & Newton 284046 (EA).

***Carallumadicapuae* (Chiov.) Chiov.** Habit: Herb. Habitat: Rocky bushland, 700–1700 m. Voucher: FOKP 1260 (EA, HIB).

***Carallumapeckii* P.R.O. Bally** Habit: Herb. Habitat: Rare in rocky bushland, 900–1150 m. Voucher: Bally 12359 (EA).

***Carissaedulis* (Forssk.) Vahl** Habit: Shrub. Habitat: Bushland, thicket, grassland, 1–2550 m. Vouchers: FOKP 11681 (EA), Mbuni 681 (EA).

***Carissaspinarum* L.** Habit: Shrub. Habitat: Forest edges woodland, 1–2550 m. Vouchers: FOKP 1109, 1272 & 11439 (EA, HIB), Mbuni 175 (EA).

***Ceropegiaballyana* Bullock** Habit: Climber. Habitat: Dry bushland, 900–1400 m. Voucher: Masinde 683 (EA).

***Ceropegiaracemosa* N.E. Br.** Habit: Climber. Habitat: Evergreen forest, 1–1900 m. Voucher: FOKP 1822 (EA, HIB).

**Gomphocarpusfruticosussubsp.flavidus (N.E. Br.) Goyder** Habit: Shrub. Habitat: Upland grassland, 900–2900 m. Vouchers: Napier 2039 (EA), Hepper 5056 (EA).

***Gomphocarpusphillipsiae* (N.E. Br.) Goyder** Habit: Shrub. Habitat: Upland grassland, 1600–3050 m. Vouchers: FOKP 11279 (EA, HIB), Mbuni 015 (EA).

***Gomphocarpusphysocarpus* E. Mey.** Habit: Shrub. Habitat: Upland grassland, 1800–3000m. Vouchers: SAJIT 004771 (EA, HIB), FOKP 11300 (EA, HIB), Mbuni 056 (EA, HIB).

***Gomphocarpusstenoglossus* Schltr.** Habit: Herb Habitat: Grassland, rocky soils, 1600–3050 m. Voucher: FOKP 1817 (EA, HIB).

***Landolphiabuchananii* (Hallier f.) Stapf** Habit: Climber. Habitat: Riverine, 1350–2150 m. Vouchers: FOKP 1270 & 11273 (EA, HIB).

***Leptadenialancifolia* (Schumach. & Thonn.) Decne.** Habit: Climber. Habitat: Alluvial & Riverine bushland, 1650–2600 m. Voucher: Balley 12360 (EA).

***Margarettarosea* Oliv.** Habit: Herb. Habitat: Grasslands, 1600–2500 m. Voucher: Symes 280 (EA).

***Marsdeniarobusta* Balf. f** Habit: Climber. Habitat: bushland, 1600–2000 m. Voucher: FOKP 11310 (EA, HIB).

***Marsdeniaangolensis* N.E. Br.** Habit: Climber. Habitat: Shrubland and Riverine bushland, 1600–2000 m. Voucher: Tweedie 2403 (EA).

***Marsdeniarubicunda* N.E. Br.** Habit: Climber. Habitat: Riverine, bushland in dry areas, 0–1300 m. Voucher: Goyder & Masinde 3952 (EA).

***Marsdeniaschimperi* Decne.** Habit: Climber. Habitat: Upland forest edge, 1800–2650 m. Vouchers: SAJIT 006886 (EA, HIB), FOKP 1884 & 10986 (EA, HIB).

***Mondiawhitei* (Hook. f.) Skeels.** Habit: Climber. Habitat: 1600–2000 m. Voucher: FOKP 998 (EA, HIB).

***Pachycarpuseximius* (Schltr.) Bullock.** Habit: Herb. Habitat: Uncommon in grassland, 1600–2500 m. Voucher: Haper & Field 5038 (EA).

***Pachycarpusgrantii* (Oliv.) Bullock** Habit: Herb. Habitat: Montane grassland, 1850–2950 m. Voucher: Hepper et al. 5038 (EA).

***Pachycarpuslineolatus* (Decne.) Bullock** Habit: Herb. Habitat: Dry wooded grassland, 1000–2700 m. Voucher: Napier 2019 (EA).

***Pachycarpuspetherickianus* (Oliv.) Goyder.** Habit: Herb. Habitat: 1000–2000 m. Voucher: Tweedie 380 (EA).

***Pentarrhinumgonoloboides* (Schltr.) Liede** Habit: Climber. Habitat: Montane forest, 2500–3200 m. Voucher: Tweedie 3912 (EA).

***Pergulariadaemia* (Forssk.) Chiov.** Habit: Herb. Habitat: Upland forest, around 2050 m. Vouchers: FOKP 1268 & 11481 (EA, HIB), Mbuni 217 & 749 (EA).

***Periplocalinearifolia* Quart.–Dill. & A. Rich.** Habit: Climber. Habitat: Upland forest, 1900–2900 m. Vouchers: SAJIT 005059 (EA, HIB), FOKP 1076 (EA, HIB), Mbuni 044 & 622 (EA, HIB), Bridson 104 (EA), Fattan 13876 (EA).

***Sabacomorensis* (Bojer ex A. DC.) Pichon.** Habit: Shrub. Habitat: Riverine forest, 1–1800 m. Voucher: Mabberley & McCall 98 (EA).

***Secamoneattenuifolia* Goyder** Habit: Shrub. Habitat: Bushland, wooded grassland, 1000–1850 m. Voucher: Tweedie 3638 (EA).

***Stathmostelmapedunculatum* (Decne.) K. Schum.** Habit: Herb. Habitat: Sandy grassland, 0–2300 m. Voucher: Lucas 190 (EA).

***Tabernaemontanastapfiana* Britten** Habit: Tree. Habitat: Wet forest, 1500–2300 m. Vouchers: SAJIT 006887 (EA, HIB), FOKP 1112, 10987 (EA, HIB).

***Tylophoraheterophylla* A. Rich.** Habit: Climber. Habitat: Upper forest, 2600–3500 m. Vouchers: FOKP 11600 (EA), Mbuni 596 & 600 (EA), Mabberley & McCall 65 (EA).

***Tylophoralugardae* Bullock** Habit: Climber. Habitat: Upland forest, 2000–2500 m. Voucher: Archer 303 (EA).

***Tylophoratenuipedunculata* K. Schum**. Habit: Climber. Habitat: Upland forest up to 1500 m. Vouchers: FOKP 11453 (EA, HIB), Mbuni 189 (EA).

***Xysmalobiumheudelotianum* Decne.** Habit: Herb. Habitat: Dry grassland, 1500–2000 m. Voucher: Napier 1974 (EA).

**F109. Boraginaceae**

6 Genus, 11 Species

***Cordiaafricana* Lam.** Habit: Tree. Habitat: Riverine, forest, wooded grassland, 1050–2100 m. Voucher: SAJIT 005062 (EA, HIB).

***Cynoglossumamplifolium* Hochst. ex A. DC.** Habit: Herb. Habitat: Montane forest, 2100–3430 m. Voucher: Irwin 423 (EA).

***Cynoglossumaequinoctiale* T.C.E. Fr.** Habit: Herb. Habitat: Montane grassland, 1770–2480 m. Voucher: Thulin & Tidigs 77 (EA).

***Cynoglossumcheranganiense* Verdc.** Habit: Herb. Habitat: Rare in alpine grassland, heath and upper forest zone, 2480–3255 m. Vouchers: Tweedie 2984 (EA), Mabberley 207 (EA), Lucas 168 (EA).

***Cynoglossumcoeruleum* Hochst. ex A. DC.** Habit: Herb. Habitat: Montane forest, 1100–3200 m. Voucher: FOKP 1245 & 11435 (EA, HIB), Mbuni 171 (EA), Dale 15331 (EA).

***Ehretiacymosa* Thonn.** Habit: Shrub. Habitat: Evergreen forest, dry or wet forest patches, 1050–2300 m. Vouchers: FOKP 1882 & 11625 (EA, HIB), Mbuni 625 (EA).

***Lithospermumafromontanum* Weim.** Habit: Shrub. Habitat: Montane forest edges, heath woodland, 2100–3350 m. Vouchers: Tweedie 3828 (EA), Thulin & Tidigs 239 (EA).

***Myosotisabyssinica* Boiss. & Reut.** Habit: Herb. Habitat: Montane forest edges, heath woodland, 1560–3590 m. Voucher: Thulin & Tidings 236 (EA).

***Myosotiskeniensis* T.C.E. Fr.** Habit: Herb. Habitat: Alpine zones, 3150–3350 m. Voucher: Thulin & Tidigs 236 (EA).

***Trichodesmaphysaloides* (Fenzl) A. DC.** Habit: Herb. Habitat: Wooded grassland, 700–2400 m. Voucher: Mortimer 275 (EA).

***Trichodesmazeylanicum* (Burm. f.) R. Br.** Habit: Herb. Habitat: Dry bushland, 250–1710 m. Vouchers: FOKP 11281 (EA), Mbuni 017 (EA).

**F110. Convolvulaceae**

7 Genera, 15 Species

***Astripomoealachnosperma* (Choisy) A. Meeuse** Habit: Herb. Habitat: Bushland, 750–2600 m. Voucher: Napier 2035 (EA, HIB).

***Astripomoeamalvacea* (Klotzsch) A. Meeuse**. Habit: Shrub. Habitat: Wooded grassland, 725–2240 m. Voucher: Napier 2074 (EA).

***Convolvuluskilimandschari* Engl.** Habit: Climber. Habitat: Bamboo thicket, 2400–3500 m. Voucher: FOKP 1062 (EA, HIB).

***Convolvuluswightii* Wall.** Habit: Herb. Habitat: Grassland, 1500–2500 m. Voucher: FOKP 1292 (EA, HIB).

***Cuscutakilimanjari* Oliv.** Habit: Climber. Habitat: Common in forest areas, 1500–2770 m. Voucher: SAJIT 006901 (EA, HIB).

***Cuscutaepilinum* Weihe** Habit: Climber. Habitat: Cultivated ground, 1800–2250 m. Voucher: Schouten 58 & H348 (EA).

***Cuscutaplaniflora* Ten.** Habit: Climber. Habitat: Occurring on various hosts, 1500–3000 m. Vouchers: FOKP 11553 (EA, HIB), Mbuni 290 (EA).

***Ipomoeacairica* (L.) Sweet.** Habit: Herb. Habitat: Swampy grassland, 6000–1950 m. Voucher: SAJIT Z0056 (EA).

***Ipomoeatenuirostris* Choisy** Habit: Herb. Habitat: Bushland and thicket, 1650–2710 m. Vouchers: FOKP 1885 & 11510 (EA, HIB), Mbuni 246 (EA), Tweedie 3115 (EA), Webster 8924 (EA).

***Ipomoeacrepidiformis* Hallier f.** Habit: Herb. Habitat: Wooded grassland, 900–2150 m. Voucher: Champion T3 (EA).

***Ipomoeaspathulata* Hallier f.** Habit: Shrub. Habitat: *Acacia–Commiphora* Bushland, 610–1640 m. Vouchers: FOKP 11440 (EA) & Mbuni 176 (EA).

***Ipomoeakituiensis* Vatke.** Habit: Herb. Habitat: Dry bushland, 180–1860 m. Vouchers: SAJIT 006850 (EA, HIB), FOKP 11510 & 10950 (EA, HIB), Mbuni 246 (EA).

***Ipomoeatenuirostris* Choisy** Habit: Herb. Habitat: Bushland and thicket, 1650–2710 m. Voucher: Tweedie 3115 (EA).

***Stictocardiaberaviensis* (Vatke) Hallier f.** Habit: Shrub. Habitat: Bushland, 900–1800 m. Voucher: Honore 3451 (EA).

***Turbina stenosiphon* (Hallier f.) A. Meeuse** Habit: Climber. Habitat: Bushland and in rocky areas, 900–1800 m. Voucher: Tweedie 1926 (EA).

**F111. Solanaceae**

5 Genera, 18 Species

***Cestrumaurantiacum* Lindl.** Habit: Shrub. Habitat: Cultivated and disturbed ground, 1000–2500 m. Vouchers: FOKP 1020 (EA, HIB), Mbuni 085 (EA, HIB).

***Discopodiumpenninervium* Hochst.** Habit: Shrub. Habitat: Bamboo zone, 2900–3300 m. Voucher: FOKP 1891 (EA, HIB).

***Physalislagascae* Roem. & Schult.** Habit: Herb. Habitat: Cultivated and disturbed ground, 40–1850 m. Voucher: Meyerhoff 12 (EA).

***Physalisperuviana* L.** Habit: Herb. Habitat: Cultivated and naturalized, 1700–2480 m. Voucher: Mabberley & McCall 108 (EA).

***Solanumaculeastrum* Dunal.** Habit: Shrub. Habitat: Forest margin, 1800–2650 m. Vouchers: FOKP 935 & 11364 (EA, HIB), Mbuni 100 (EA), Symes 635 (EA).

***Solanumaculeatissimum* Jacq.** Habit: Herb. Habitat: Montane forest, 1425–2640 m. Voucher: FOKP 11397 (EA), Mbuni 133 (EA), Hepper & Field 4985 (EA).

***Solanumanguivi* Lam.** Habit: Shrub. Habitat: Cultivated, 1230–2700 m. Vouchers: SAJIT 005143 (EA, HIB), FOKP 966 & 11535 (EA, HIB), Mbuni 271 (EA), Hepper & Field 4986 (EA).

***Solanumbetaceum* Cav.** Habit: Shrub. Habitat: Cultivated, 1200–2800 m. Voucher: FOKP 1879 (EA, HIB).

***Solanumcampylacanthum* Hochst.** Habit: Shrub. Habitat: Cultivated, 1030–2000 m. Vouchers: FOKP 1296 & 11480 (EA, HIB), Mbuni 216 (EA).

***Solanumgiganteum* Jacq.** Habit: Shrub. Habitat: Forest margin, Undergrowth, 1500–2400 m. Vouchers: FOKP 11365 (EA, HIB) & Mbuni 100 (EA).

***Solanumhastifolium* Dunal** Habit: Shrub. Habitat: Dry bushland or grassland, 50–1400 m. Voucher: Carter & Stannard 59 (EA).

***Solanummauense* Bitter** Habit: Shrub. Habitat: Secondary forest, 1800–2700 m. Vouchers: SAJIT 004754 & 004774 (EA, HIB), FOKP 11291 (EA, HIB), Mbuni 027 (EA).

***Solanummauritianum* Scop.** Habit: Tree. Habitat: Cultivation, 1450–2750 m. Vouchers: FOKP 1069 & 11397 (EA, HIB), Mbuni 133 (EA).

***Solanumnakurense* C.H. Wright.** Habit: Shrub. Habitat: Upland bushland, 900–2950 m. Vouchers: FOKP 1006 & 11605 (EA, HIB), Mbuni 605 (EA), Hepper & Field 4976 (EA), Webster 8918 (EA).

***Solanumnigrum* L.** Habit: Shrub. Habitat: Cultivated, 0–3070 m. Vouchers: FOKP 11341 (EA, HIB), Mbuni 077 (EA), Webster 8918 (EA).

***Solanumsessilistellatum* Bitter**. Habit: Shrub. Habitat: Montane rain forest, 2130–2990 m. Vouchers: FOKP 11379 (EA, HIB), Mbuni 115 (EA), Jackson 2424 (EA).

***Solanumterminale* Forssk.** Habit: Shrub. Habitat: Riverine, thickets, 1350–2950 m. Vouchers: SAJIT 004736 (EA, HIB), FOKP 1888, 11297 (EA, HIB), Mbuni 033 (EA), Irwin 139 (EA).

***Withaniasomnifera* (L.) Dunal.** Habit: Shrub. Habitat: Forest margin, 450–2250 m. Vouchers: FOKP 11384 & 14477 (EA, HIB), Mbuni 119 & 213 (EA).

**F112. Oleaceae**

2 Genera, 5 Species

***Jasminumabyssinicum* Hochst. ex DC.** Habit: Climber. Habitat: Upland forest edges, bushland, open and riverine, 900–3000 m. Vouchers: SAJIT 005088 (EA, HIB), FOKP 11353 (EA, HIB), Webster 8823 (EA).

***Jasminumfluminense* Vell.** Habit: Climber. Habitat: Dry *Combretum, Albizia* woods, 280–2700 m. Vouchers: SAJIT 006807 (EA, HIB), FOKP 10907 (EA, HIB).

**Oleaeuropaeasubsp.africana (Mill.) P.S. Green.** Habit: Tree. Habitat: Upland forest, 1150–2550 m. Voucher: SAJIT 004756 (EA, HIB).

**Oleaeuropaeasubsp.cuspidata (Wall. & G. Don) Cif.** Habit: Tree. Habitat: Upland evergreen forest, 950–2400 m. Voucher: FOKP 1798 (EA, HIB).

***Oleaafricana* Mill.** Habit: Tree. Habitat: Upland forest, 1150–2550 m. Voucher: Lind 5088 (EA).

**F113. Plantaginaceae**

4 Genera, 5 Species

***Callitricheoreophila* Schotsman.** Habit: Herb. Habitat: Bamboo zone, 1900–3500 m. Voucher: Townsend 2389 (EA).

***Plantagopalmata* Hook. f.** Habit: Herb. Habitat: Montane rain forest, Path sides, 1704–3550 m. Vouchers: FOKP 1065 & 11552 (EA, HIB), Mbuni 289 (EA), Pudwa 67 (EA).

***Plantagolanceolata* L.** Habit: Herb. Habitat: Path sides, 1650–2750 m. Voucher: Tweedie 2806 (EA).

***Sibthorpiaeuropaea* L.** Habit: Herb. Habitat: Alpine zone, 1860–2345 m. Voucher: Mabberley 576 (EA).

***Veronicaabyssinica* Fresen.** Habit: Herb. Habitat: Upland grassland, woodland edges, 1560–3500 m. Voucher: Symes 516 (EA).

**F114. Scrophulariaceae**

7 Genera, 7 Species

***Alectraasperrima* Benth.** Habit: Herb. Habitat: Montane wet grassland, 2000–3000 m. Voucher: Thorold 2761 (EA).

***Buddlejapolystachya* Fresen.** Habit: Tree. Habitat: Montane forest, Bushland, 1000–2700 m. Vouchers: SAJIT 006895 (EA, HIB), FOKP 965, 11319, 11327 & 11393 (EA, HIB), Mbuni 055 & 128 (EA).

***Cycniumtenuisectum* (Standl.) O.J. Hansen** Habit: Tree. Habitat: Bushland, 1000–2700 m. Voucher: SAJIT 007119 (EA, HIB).

***Hebenstretiadentata* L.** Habit: Shrub. Habitat: Grassland at around 2300 m. Voucher: Thulin 119 (EA).

***Limosellaafricana* Glück** Habit: Herb. Habitat: Upland grassland, 1600–3500 m. Voucher: Mabberley 570 (EA).

***Selagothomsonii* Rolfe.** Habit: Shrub. Habitat: Dry subalpine heathland, 1860–3380 m. Vouchers: Dale 3395 (EA), Part II Botany 5159 (EA).

***Verbascumscrophulariifolium* (Hochst.) Hub.-Mor.** Habit: Herb. Habitat: Upland grassland, 1950–2635 m. Voucher: Thulin & Tidigs 44 (EA).

**F115. Stilbaceae**

3 Genera, 4 Species

***Hallerialucida* L.** Habit: Tree. Habitat: Montane forest, Drier forests associated with cedar, podo and Bamboo, 1550–2750 m. Vouchers: SAJIT 005142 & 006802 (EA, HIB), FOKP 1014, 10902 & 11354 (EA, HIB), Mbuni 619 (EA), Agnew et al. 5076 (EA).

***Hebenstretiaangolensis* Rolfe.** Habit: Shrub. Habitat: Rocky heathlands, 1500–3500 m. Vouchers: Mbuni 125 (EA), Bogdan 4390 (EA).

***Nuxiacongesta* R. Br. ex Fresen.** Habit: Tree. Habitat: Montane forest, bamboo Zone, 1550–2850 m. Vouchers: SAJIT 005068 (EA, HIB), FOKP 11350 & 11370 (EA, HIB).

***Nuxiaoppositifolia* (Hochst.) Benth.** Habit: Tree. Habitat: Riverine forest, bushland, 1550–2850 m. Voucher: Honare 3716 (EA).

**F116. Linderniaceae**

3 Genera, 7 Species

***Craterostigmaplantagineum* Hochst.** Habit: Herb. Habitat: Over rocks, 1160–2300 m. Voucher: Hepper & Field 5063 (EA).

***Craterostigmapumilum* Hochst.** Habit: Herb. Habitat: Over rocks, in montane, 1800–3300 m. Voucher: Hepper & Field 5026 (EA).

***Cycniumadonense* E. Mey. ex Benth.** Habit: Herb. Habitat: Wooded grassland, 0–2550 m. Vouchers: Lucas 194 (EA), Symes 32 (EA).

***Linderniaabyssinica* Engl.** Habit: Herb. Habitat: Shallow soil, 1800–2600 m. Voucher: Maas 4744 (EA).

***Linderniapulchella* (Skan) Philcox** Habit: Herb. Habitat: Short grassland, 400–1800 m. Voucher: Tweedie 3810 (EA).

***Linderniarotundifolia* (L.) Alston** Habit: Herb. Habitat: Grassy marshes, 1650–1900 m. Vouchers: FOKP 1146, 1236 & 1795 (EA, HIB).

***Linderniaschweinfurthii* (Engl.) Dandy** Habit: Herb. Habitat: Shallow soils, 1700–1850 m. Voucher: Lucas 174 (EA).

**F117. Pedaliaceae**

1 Genus, 2 Species

***Sesamumangolense* Welw.** Habit: Shrub. Habitat: Grassland and roadside, 400–2400 m. Voucher: FOKP 1039 (EA, HIB).

***Sesamumcalycinum* Welw.** Habit: Herb. Habitat: Grassland and wasteland, 310–1340 m. Voucher: FOKP 1039 (EA, HIB).

**F118. Lamiaceae**

32 Genera, 68 Species

***Achyrospermumschimperi* (Hochst. ex Briq.) Perkins** Habit: Shrub. Habitat: Wet streamside, 1600–3000 m. Vouchers: FOKP 11311 (EA), Mbuni 047 (EA).

***Ajugaremota* Benth.** Habit: Herb. Habitat: Montane forest, 1500–3150 m. Vouchers: FOKP 11473 (EA, HIB), Mbuni 209 (EA).

***Aeollanthusdensiflorus* Ryding** Habit: Herb. Habitat: wooded grassland, Rocky slopes 1730–2300 m. Voucher: FOKP 1813 (EA, HIB).

***Aeollanthusrepens* Oliv**. Habit: Shrub. Habitat: Wooded grassland, 1550–2700 m. Vouchers: FOKP 1031, Thulin & Tidigs 196 (EA).

***Beciumdecumbens* (Gürke) A.J. Paton** Habit: Herb. Habitat: grassland, 1550–3200 m. Voucher: Townsend 2355 (EA).

***Bridsonrotundifolium* (Briq.) A.J. Paton** Habit: Herb. Habitat: Wooded grassland, 1500–2800 m. Voucher: Bridson 102 (EA).

***Clerodendrumcapense* Eckl. & Zeyh. ex Schauer** Habit: Shrub. Habitat: Wooded grassland, 1200–1900 m. Voucher: SAJIT 005081 (EA, HIB).

***Clerodendrumjohnstonii* Oliv.** Habit: Shrub. Habitat: Wet forest margin, 1200–2500 m. Vouchers: SAJIT 004723, 005081 & 006817 (EA, HIB), FOKP 10917 & 11432 (EA, HIB), Mbuni 168 & 739 (EA, HIB), Tweedie 4201 (EA).

***Clerodendrumumbellatum* Poir.** Habit: Herb. Habitat: Wooded grassland, up to 1860 m. Vouchers: Napier 2007 (EA), Tweedie 2732 (EA).

***Clinopodiumabyssinicum* (Benth.) Kuntze** Habit: Herb. Habitat: Woodland, 1000–3000 m. Voucher: Verdcourt 2439 (EA).

***Fuerstiaafricana* T.C.E. Fr.** Habit: Herb. Habitat: Grassland, 800–2400 m. Vouchers: FOKP 11295, 11431 & 11728 (EA, HIB), Mbuni 031, 167 & 728 (EA).

***Geniosporumpaludosum* Baker.** Habit: Herb. Habitat: Grassland, 1000–2500 m. Voucher: Ivens 1253 (EA).

***Hoslundiaopposita* Vahl.** Habit: Shrub. Habitat: Bushland wooded grassland, 1–2000 m. Vouchers: FOKP 11441 (EA, HIB), Mbuni 177 (EA), Webster 8969 AE.

***Leonotismollissima* Gürke.** Habit: Herb. Habitat: Disturbed grassland, 1500–2600 m. Voucher: SAJIT 004845 (EA, HIB).

***Leonotisnepetifolia* (L.) R. Br.** Habit: Shrub. Habitat: Disturbed grassland, 900–2300 m. Voucher: SAJIT 004845 (EA, HIB).

**Leonotisocymifoliavar.raineriana (Vis.) Iwarsson.** Habit: Shrub. Habitat: Montane forest zone, 1770–3360 m. Vouchers: FOKP 942 & 11311 (EA, HIB), Part II Botany 53 (EA).

***Leucas argentea Gürke.*** Habit: Shrub. Habitat: Upland grassland, 1200–2710 m. Vouchers: Thulin & Tidigs 123 (EA), Dale 3408 (EA).

**Leucascalostachysvar.fasciculata (Baker) Sebald.** Habit: Herb. Habitat: Grassland, 1600–2800 m. Vouchers: FOKP 1217, 11348 & 11371 (EA, HIB), Mbuni 10 & 754 (EA).

***Leucasdeflexa* Hook. f.** Habit: Herb. Habitat: Upland grassland, 1600–2600 m. Vouchers: SAJIT 006803 (EA, HIB), FOKP 10903 & 11312 (EA, HIB), Mbuni 048 (EA).

***Leucasmasaiensis* Oliv.** Habit: Herb. Habitat: Upland grassland, 1280–2735 m. Voucher: Thulin & Tidigs 272 (EA).

***Leucasmartinicensis* (Jacq.) R. Br.** Habit: Herb. Habitat: Disturbed soil especially on farmland, 0–2200 m. Vouchers: FOKP 11696 (EA), Mbuni 696 (EA).

***Leucasoligocephala* Hook. f.** Habit: Shrub. Habitat: Wooded grassland, 1600–2990 m. Voucher: Symes 753 (EA).

***Lippiajavanica* (Burm. f.) Spreng.** Habit: Shrub. Habitat: Wooded bushland, 1300–2100 m. Vouchers: FOKP 11448 (EA, HIB), Mbuni 184 (EA).

***Menthaaquatica* L.** Habit: Herb. Habitat: Upland forest, 1950–2600 m. Voucher: Symes 213 (EA).

***Micromeriaimbricata* (Forssk.) C. Chr.** Habit: Herb. Habitat: Upland dry grassland, 1580–3600 m. Vouchers: FOKP 11546 & 14499 (EA, HIB), Mbuni 235 & 283 (EA), Thulin & Tidigs 74 (EA).

***Nepetaazurea* R. Br. ex Benth.** Habit: Herb. Habitat: Bushland, 1800–3600 m. Voucher: Mabberley & McCall 248 (EA).

***Ocimumamericanum* L.** Habit: Herb. Habitat: Bushland, Disturbed grassland, 0–1850 m. Vouchers: FOKP 11472 (EA, HIB), Mbuni 208 (EA).

***Ocimumdecumbens* Gürke.** Habit: Shrub. Habitat: Bushland, 1950–3000 m. Voucher: Maas 4754b (EA).

***Ocimumgratissimum* L.** Habit: Shrub. Habitat: Disturbed grassland, 1100–2325 m. Voucher: Symes 156 (EA).

***Ocimumkilimandscharicum* Gürke** Habit: Shrub. Habitat: Stony, Riverine grassland, 1100–2350 m. Voucher: SAJIT 004746 (EA, HIB).

***Ocimumlamiifolium* Hochst. ex Benth.** Habit: Shrub. Habitat: Montane forest, 1550–2340 m. Vouchers: FOKP 11350, 11355 & 11633 (EA, HIB), Mbuni 086 & 633 (EA, HIB), Webster 8975 (EA).

***Orthosiphonrubicundus* (D. Don) Benth.** Habit: Herb. Habitat: Wooded grassland, 1600–2635 m. Voucher: Symes 73 (EA, HIB).

***Orthosiphonschimperi* Benth.** Habit: Herb. Habitat: Wooded grassland, 1600–2635 m. Voucher: Napier 1935 (EA).

***Platostomarotundifolium* (Briq.) A.J. Paton.** Habit: Herb. Habitat: Swampy grassland, 1500–2800 m. Vouchers: SAJIT Z0021 (EA, HIB), FOKP 1222 (EA, HIB).

***Plectranthusagnewii* Lukhoba & A.J. Paton.** Habit: Herb. Habitat: Rocky grassland, 1700–1900 m. Voucher: Thomas 3396 (EA).

***Plectranthusamboinicus* (Lour.) Spreng.** Habit: Shrub. Habitat: Rocky, bushland, 130–1870 m. Voucher: Tweedie 4264 (EA).

***Plectranthusautranii* (Briq.) Erhardt, Götz & Seybold** Habit: Shrub. Habitat: Wet forest, streamside, 1500–3000 m. Voucher: Thulin & Tidigs 176 (EA).

***Plectranthusbarbatus* Andrews.** Habit: Shrub. Habitat: Bushland, rocky grassland, 880–2950 m. Vouchers: FOKP 967, 1037, 1883, 11360, 11368, & 11425 (EA, HIB), Mbuni 160 (EA), Tweedie 4263 (EA), Symes 655 (EA).

***Plectranthusbojeri* (Benth.) Hedge** Habit: Herb. Habitat: Grassland, 1200–2670 m. Voucher: Pole et al. 1514 (EA).

***Plectranthuscaninus* Roth.** Habit: Herb. Habitat: Disturbed dry rocky area, 1100–2500 m. Vouchers: SAJIT 004820 (EA, HIB), FOKP 11343 (EA, HIB).

***Plectranthusdefoliatus* Hochst. ex Benth.** Habit: Herb. Habitat: Open grassland, 2000–2880 m. Voucher: Tweedie 3728 (EA).

***Plectranthusdupuisii* (Briq.) A.J. Paton** Habit: Shrub. Habitat: Grazed grassland, 2032–3000 m. Vouchers: SAJIT Z008, Symes 752.

***Plectranthusedulis* Agnew.** Habit: Shrub. Habitat: Grassland, 2032–3000 m. Voucher: FOKP 994 (EA, HIB).

***Plectranthusgrandicalyx* (E.A. Bruce) J.K. Morton.** Habit: Shrub. Habitat: Grassland, 2000–3000 m. Voucher: Rauh 652 (EA).

***Plectranthuskamerunensis* Gürke** Habit: Shrub. Habitat: Montane forest zone, 1500–2950 m. Vouchers: FOKP 11365 (EA, HIB), Mbuni 413 (EA).

***Plectranthuslactiflorus* (Vatke) Angew** Habit: Shrub. Habitat: Wooded grassland, 1100–2500 m. Voucher: Webster 8980 (EA).

***Plectranthuslongipes* Baker** Habit: Herb. Habitat: Woodland, 700–2440 m. Voucher: SAJIT Z0046 (EA, HIB).

***Plectranthusornatus* Codd.** Habit: Herb. Habitat: *Acacia, Commiphora* bushland, 1000–2790 m. Voucher: Gillett 18495 (EA).

***Plectranthuspunctatus* (L. f.) L’Hér.** Habit: Herb. Habitat: Disturbed montane, bamboo forest, 1950–3350 m. Vouchers: FOKP 1132 (EA, HIB), Mbuni 090 (EA), Thulin & Tidigs 51 (EA).

***Plectranthustetradenifolius* A.J. Paton** Habit: Herb. Habitat: Rocky places, 950–2325 m. Voucher: Mathew 6170 (EA).

***Plectranthusxylopodus* Lukhoba & A.J. Paton** Habit: Herb. Habitat: Dry grassland, bushland, 100–1400 m. Vouchers: SAJIT 004837 (EA, HIB), Cymes 293 (EA).

***Pycnostachyseminii* Gürke** Habit: Shrub. Habitat: Riverine and forest margin, 1600–1800 m. Vouchers: FOKP 11734 (EA), Mbuni 734 (EA).

***Pycnostachysmeyeri* Gürke ex Engl.** Habit: Herb. Habitat: Bamboo zone, secondary bushland, 1900–3000 m. Vouchers: FOKP 11274, 11301 & 11346 (EA, HIB), Mbuni 010 (EA).

***Pycnostachysstuhlmannii* Gürke**. Habit: Herb. Habitat: Marshes, 1860–2400 m. Voucher: Symes 202 (EA).

***Rothecamyricoides* (Hochst.) Steane & Mabb.** Habit: Shrub. Habitat: Bushland, 1180–2120 m. Vouchers: FOKP 11566 & 11691 (EA), Mbuni 303, 691 (EA).

***Salviamerjamie* Forssk.** Habit: Herb. Habitat: Grassland, 1800–3500 m. Voucher: Lucas 167 (EA).

***Salvianilotica* Juss. ex Jacq.** Habit: Herb. Habitat: Grassland, 1800–3500 m. Vouchers: FOKP 11534 & 11704 (EA, HIB), Mbuni 270 & 704 (EA), Symes 75 (EA).

***Scutellariapaucifolia* Baker** Habit: Herb. Habitat: Short grassland, 1550–2200 m. Voucher: Webster 8978 (EA).

***Scutellariaschweinfurthii* Briq.** Habit: Herb. Habitat: Short grassland, 1550–1950 m. Voucher: Symes 33 (EA).

***Scutellariaviolascens* Gürke** Habit: Herb. Habitat: wooded grassland, 1920–2480 m. Vouchers: SAJIT 005107 (EA, HIB), Chater 1932 (EA).

***Saturejaabyssinica* (Benth.) Briq.** Habit: Herb. Habitat: Wooded grassland, 1920–2500 m. Vouchers: FOKP 1085, Bridson 101 (EA).

***Micromeriabiflora* (Buch.-Ham. ex D. Don) Benth.** Habit: Habitat: Grassland, 1580–3300 m. Voucher: FOKP 1223 (EA, HIB).

***Saturejaimbricata* (Forssk.) Briq.** Habit: Shrub. Habitat: Dry grassland, 1580–3500 m. Voucher: Part II Botany 79 (EA).

***Saturejapseudosimensis* Brenan.** Habit: Shrub. Habitat: Grassland, 1400–2900 m. Voucher: Symes 622 (EA).

***Stachysalpigena* T.C.E. Fr.** Habit: Herb. Habitat: Alpine zone, 2940–3550 m. Vouchers: FOKP 11325 (EA, HIB), Mbuni 061 (EA).

***Tetradeniariparia* (Hochst.) Codd.** Habit: Shrub. Habitat: Rocky slopes, 600–2200 m. Voucher: SAJIT 004839 (EA, HIB).

***Tinneaaethiopica* Kotschy ex Hook. f.** Habit: Shrub. Habitat: Forest thicket, forest margin, Upland woodland up to 2200 m. Vouchers: FOKP 11692, Mbuni 692 (EA), Lucas 160 (EA).

***Rothecamyricoides* (Hochst.) Steane & Mabb.** Habit: Shrub. Habitat: Grazed bushland, 1180–2120 m. Vouchers: FOKP 11566 (EA, HIB), Mbuni 303 (EA).

**F119. Orobanchaceae**

9 Genera, 14 Species

***Alectraparasitica* A. Rich.** Habit: Parasitic herb. Habitat: Dry areas, 434–1670 m. Voucher: Mabberley & McCall 276 (EA).

***Alectrasessiliflora* (Vahl) Kuntze.** Habit: Parasitic herb. Habitat: Dry areas, 500–2000 m. Voucher: Bogdan 5302 (EA).

***Bartsialongiflora* Hochst. ex Benth.** Habit: Shrub. Habitat: Heath vegetation, 2450–3480 m. Voucher: Mabberley 299 (EA).

***Bartsiatrixago* L.** Habit: Herb. Habitat: Heath vegetation, 900–3100 m. Vouchers: Symes 645 (EA), Tweedie 4201 (EA), Mabberley 589 (EA).

***Buchneranuttii* Skan.** Habit: Parasitic herb. Habitat: Wooded grassland, 1300–2170 m. Vouchers: Gardener 3723 (EA), Webster 8979 (EA).

***Buchnerascabridula* E.A. Bruce** Habit: Herb. Habitat: Grassland, 2640–2940 m. Vouchers: Mabberley & McCall 303 (EA), Irwin 370 (EA).

***Buttonianatalensis* McKen ex Benth** Habit: Climber. Habitat: Dry bushland, 1800 –1860 m. Voucher: SAJIT 005142 (EA, HIB).

***Cycniumtenuisectum* (Standl.) O.J. Hansen** Habit: Herb. Habitat: Upland grassy marshes, 1800–3200 m. Vouchers: SAJIT 005103 (EA, HIB), Thulin &Tidigs 129 (EA), Lucas 213 (EA).

***Hedbergiaabyssinica* (Benth.) Molau** Habit: Herb. Habitat: Upland grassland around, 2700 m. Voucher: Thulin & Tidigs 157 (EA).

***Orobancheramosa* L.** Habit: Herb. Habitat: Upland grassland, 1735–2250 m. Voucher: SAJIT 004782 (EA, HIB).

***Orobancheminor* Sm**. Habit: Herb. Habitat: Upland grassland, 800–2480 m. Vouchers: SAJIT 006900 (EA, HIB), Thulin & Tidigs 177 (EA).

**Sopubiakaraguensisvar.welwitschii (Engl.) O.J. Hansen** Habit: Herb. Habitat: Montane grassland, 500–3400 m. Voucher: Tweedie 2980 (EA).

***Sopubiaramosa* (Hochst.) Hochst.** Habit: Herb. Habitat: Wooded grassland, 1600–2170m. Vouchers: Graham 324 (EA), Agnew et al. 10581 (EA), Gardner 3724 (EA).

***Strigaasiatica* (L.) Kuntze** Habit: Parasitic herb. Habitat: Upland grassland, 0–2480 m. Voucher: Mabberley 86 (EA).

**120. Lentibulariaceae**

2 Genera, 4 Species

***Genliseahispidula* Stapf** Habit: Herb. Habitat: Grassland pools, 1860–2400 m. Voucher: Thulin & Tidings 182 (EA).

***Utriculariaarenaria* A. DC.** Habit: Herb. Habitat: Seasonal swamps, 380–2950 m. Voucher: FOKP 1140 (EA, HIB).

***Utricularialivida* E. Mey.** Habit: Herb. Habitat: Seasonal pools, 2325–2730 m. Vouchers: SAJIT 006914, FOKP 1140 & 11014 (EA, HIB).

***Utriculariaprehensilis* E. Mey.** Habit: Herb. Habitat: Upland marshes, 1860–2400 m. Voucher: Thulin & Tidings 181 (EA).

**F121. Acanthaceae**

17 Genera, 35 Species

***Acanthuseminens* C.B. Clarke** Habit: Shrub. Habitat: Dry forest, amongst bamboo, 1500–2800 m. Vouchers: FOKP 1002 (EA, HIB), Part II Botany Class 11 (EA).

***Asystasiamysorensis* (Roth) T. Anderson** Habit: Herb. Habitat: Bushland, 1160–2100 m. Voucher: SAJIT 005148 (EA, HIB).

***Barleriagrandicalyx* Lindau** Habit: Herb. Habitat: Savannah, Wooded grassland, 1100–2500 m. Vouchers: Napier 1949 (EA), Symes 35 (EA).

***Blepharisedulis* (Forssk.) Pers.** Habit: Herb. Habitat: Grassland, 140–2015 m. Voucher: SAJIT Z0066 (HIB).

***Blepharismaderaspatensis* (L.) B. Heyne ex Roth** Habit: Herb. Habitat: Grassland, bushland, 1000–3300 m. Voucher: SAJIT Z0044 (HIB).

***Crossandramassaica* Mildbr.** Habit: Herb. Habitat: Bushland, 1200–2700 m. Vouchers: FOKP 11455 & 11663 (EA), Mbuni 191 & 663 (EA).

***Crossandranilotica* Oliv.** Habit: Herb. Habitat: Bushland, 1200–1500 m. Voucher: Mabberley 793 (EA).

***Dyschoristethunbergiiflora* (S. Moore) Lindau** Habit: Shrub. Habitat: *Acacia*, *Commiphora* bush, 600–1450 m. Vouchers: SAJIT Z0040 (EA, HIB), Mabberley & McCall 265 (EA).

***Diclipteralaxata* C.B. Clarke** Habit: Herb. Habitat: Forest undergrowth, 1300–2800 m. Voucher: Part II Botany 31 (EA).

***Diclipteralatibracteata* I. Darbysh.** Habit: Herb. Habitat: Forest undergrowth, grassland, 2200–2900 m. Voucher: Hepper 5033 (EA).

***Diclipteramaculata* Nees** Habit: Climber. Habitat: Grassland, 1700–2800 m. Voucher: FOKP 11398 (EA, HIB).

***Hypoestestriflora* (Forssk.) Roem. & Schult.** Habit: Herb. Habitat: Grassland, bushland, 1600–3300 m. Vouchers: FOKP 1153 & 11305 (EA, HIB), Mbuni 041 (EA), Lind 2828 (EA).

**Hypoestesforskaoliisubsp.hildebrandtii (Lindau) I. Darbysh.** Habit: Herb. Habitat: Dry grassland, bushland forest, 0–2820 m. Vouchers: FOKP 932, 11296 & 11626, (EA, HIB), Mbuni 032 & 626 (EA, HIB).

***Hygrophilaauriculata* (Schumach.) Heine** Habit: Herb. Habitat: Grassland, about 2000 m. Voucher: Ivens 1285 (EA).

***Hygrophilaschulli* M.R. Almeida & S.M. Almeida** Habit: Herb. Habitat: Bushland, up to 2170 m. Voucher: Ivens 1285 (EA).

***Isoglossagregoryi* (S. Moore) Lindau** Habit: Herb. Habitat: Montane and bamboo forest, 1900–2820 m. Voucher: FOKP 1745 (EA, HIB).

***Isoglossasubstrobilina* C.B. Clarke** Habit: Herb. Habitat: Montane rain forest, 1600–2200 m. Vouchers: FOKP 948 1120 & 1747 (EA, HIB).

***Justiciaanagalloides* (Nees) T. Anderson** Habit: Herb. Habitat: Grassland, 1020–2500 m. Vouchers: Geesteranus 4785 (EA), Symes 39 (EA).

***Justiciabetonica* L.** Habit: Herb. Habitat: Grassland, Wet forests, riversides, 700–2220 m. Voucher: Tweedie 2958 (EA).

***Justiciacalyculata* Deflers** Habit: Herb. Habitat: Bushland, 1000–2200 m. Voucher: SAJIT Z0043 (HIB).

***Justiciaexigua* S. Moore.** Habit: Herb. Habitat: Grassland, 1000–2200 m. Voucher: Symes 272 (EA).

***Justiciadiclipteroides* Lindau** Habit: Herb. Habitat: Woodland 1600–2325 m. Vouchers: FOKP 1088 & 11555 (EA, HIB), Mbuni 292 & 744 (EA), Geesteranus 4793 (EA).

***Justiciaflava* (*Forssk.*) Vahl** Habit: Herb. Habitat: Wooded grassland, 1400–2300 m., Vouchers: SAJIT 004725 (EA, HIB), Geesteranus 6375 (EA).

***Justicialeikpiensis* S. Moore** Habit: Herb. Habitat: Grassland, 2130–2480 m. Vouchers: Napier 1914 (EA), Mainwaring 18 (EA).

***Justiciapinguior* C.B. Clarke** Habit: Herb. Habitat: Wooded grassland, 1720–2170 m. Voucher: Symes 40 (EA).

***Justiciaunyorensis* S. Moore.** Habit: Herb. Habitat: Montane rain forest, 2170–3100 m. Voucher: Trelawny 4389 (EA).

***Justiciauncinulata* Oliv. ex C.B. Clarke** Habit: Herb. Habitat: Wooded grassland, 1700–2200 m. Voucher: Symes 39 (EA).

***Lepidagathiscollina* (Endl.) Milne–Redh.** Habit: Herb. Habitat: Bushland, 1800–2170 m. Voucher: Heliz et al. 853 (EA).

***Megalochlamyskenyensis* Vollesen** Habit: Shrub. Habitat: Wooded grassland, bushland, 1050–1450 m. Voucher: Tweedie 4265 (EA).

***Megalochlamysviolacea* (Vahl) Vollesen.** Habit: Shrub. Habitat: *Acacia* bushland, 150–1420 m. Voucher: Tweedie 4265 (EA).

***Mimulopsissolmsii* Schweinf.** Habit: Herb. Habitat: Bushland, 1700–2300 m. Vouchers: SAJIT Z0009 (EA, HIB), FOKP 1073 & 1202 (EA, HIB), Tweedie 3229 (EA).

***Schaueriapaniculata* Nees** Habit: Herb. Habitat: Bushland, 1800–2170 m. Vouchers: FOKP 11484 (EA), Mbuni 220 (EA).

***Thunbergiaalata* Bojer ex Sims** Habit: Herb. Habitat: Grassland, bushland, 100–3000 m. Voucher: FOKP 11275 & 11568 (EA, HIB), Mbuni 305 & 662 (EA), Tweedie 2703 (EA).

***Thunbergiapaulitschkeana* Beck** Habit: Herb. Habitat: Grassland, 1200–3000 m. Voucher: Hepper 5024 (EA).

**F122. Bignoniaceae**

1 Genus, 1 Species

***Stereospermumkunthianum* Cham.** Habit: Tree. Habitat: Rocky bushland, wooded grassland, 900–2100m. Voucher: Bogdan 265 (EA).

**F123. Verbenaceae**

4 Genera, 10 Species

***Hebenstreitiarariflora* A. Terracc.** Habit: Herb. Habitat: Heathland to 3550 m. Vouchers: FOKP 11390 (EA, HIB), Mbuni 125 (EA).

***Lantanatrifolia* L.** Habit: Shrub. Habitat: Bushland, 900–2350 m. Vouchers: FOKP 1036, 1287, 11322, 11685 & 11742 (EA, HIB), Mbuni 058, 685 & 742 (EA), Lind 5095 (EA).

***Lippiagrandifolia* Hochst. ex A. Rich.** Habit: Shrub. Habitat: Wooded grassland, 750–2250 m. Voucher: Lind 5096 (EA).

***Lippiajavanica* (Burm. f.) Spreng.** Habit: Shrub. Habitat: Wooded grassland, bushland, 1050–2300 m. Vouchers: FOKP 1886 & 11684 (EA, HIB), Mbuni 684 (EA).

***Lippiakituiensis* Vatke** Habit: Shrub. Habitat: Bushland, 450–2560 m. Voucher: Tweedie 3736 (EA).

***Lippiarehmannii* H. Pearson** Habit: Shrub. Habitat: Grassland, Bushland, 750–2250 m. Voucher: Symes 29 (EA).

***Lippiawoodii* Moldenke** Habit: Herb. Habitat: Burnt grassland, 1550–2170 m. Voucher: Napier 1928 (EA).

***Verbenaaristigera* S. Moore** Habit: Herb. Habitat: Open forest, 1650–2110 m. Voucher: SAJIT 004738 (EA, HIB).

***Verbenabonariensis* L**. Habit: Herb. Habitat: Bushland, Disturbed areas, 1800–2650 m. Vouchers: FOKP 1282 & 11557 (EA, HIB), Mbuni 294 (EA).

***Verbenarigida* Spreng.** Habit: Herb. Habitat: Roadside verge, 1660–2100 m. Voucher: Dyson 568 (EA).

**F124. Aquifoliaceae**

1 Genus, 1 Species

***Ilexmitis* (L.) Radlk.** Habit: Tree. Habitat: Upland riverine forest, 1450–3150 m. Vouchers: SAJIT 006912 (EA, HIB), FOKP 1013, 1227, 11374 & 11549 (EA, HIB), Mbuni 110, 286 (EA).

**F125. Campanulaceae**

4 Genera, 15 Species

***Canarinaeminii* Asch. & Schweinf.** Habit: Epiphyte. Habitat: Wet forest, 2100–2650 m. Voucher: Thulin & Tidigs 59 (EA).

***Lobeliaaberdarica* R.E. Fr. & T.C.E. Fr.** Habit: Shrub. Habitat: Moorland, Swamps, 1860–3350 m. Vouchers: Thulin & Tidigs 105 (EA), Townsend 2387 (EA).

***Lobeliacheranganiensis* Thulin** Habit: Herb. Habitat: Upland Forest & Moorland, 2750–3300 m. Vouchers: SAJIT 006863 (EA, HIB), FOKP 10963 (EA, HIB), Tweedie 3883 (EA), Knox 3379 (EA), Thulin & Tidigs 220 (EA).

***Lobeliadeckenii* (Asch.) Hemsl.** Habit: Shrub. Habitat: Moorland up to 3550 m. Voucher: Mabberley & McCall 186 (EA).

***Lobeliaduriprati* T.C.E. Fr**. Habit: Herb. Habitat: Wet montane, 1700–3550 m. Voucher: Thulin & Tidigs 220 (EA).

***Lobeliagiberroa* Hemsl.** Habit: Shrub. Habitat: Forest margin, 1200–3000 m. Vouchers: FOKP 962, 1232 & 11539 (EA, HIB), Mbuni 275 (EA), Verdcourt 2437 (EA), Knox 3790 & 3852 (EA).

***Lobeliaholstii* Engl.** Habit: Herb. Habitat: Grassland 1800–3520 m. Vouchers: SAJIT 004798, Newbould 2440 (EA).

***Lobeliaminutula* Engl.** Habit: Herb. Habitat: Disturbed alpine, 1700–3500 m. Voucher: Thulin & Tidigs 198 (EA).

***Monopsisstellarioides* (C. Presl) Urb.** Habit: Herb. Habitat: Grassland, 1650–3500 m. Vouchers: FOKP 11532 & 11540 (EA, HIB), Mbuni 276 (EA), Mabberley & McCall 97 (EA).

***Monopsiszeyheri* (Sond.) Thulin** Habit: Herb. Habitat: Wet grassland about 1600 m. Voucher: SAJIT 005137 (EA, HIB).

***Wahlenbergiacapillacea* (L. f.) A. DC.** Habit: Herb. Habitat: Bamboo zone, Rocky soils, 2300m–2950 m. Vouchers: Bogdan 4987 (EA), Symes 620 (EA).

***Wahlenbergiakrebsii* Cham**. Habit: Herb. Habitat: Grassland. Lower alpine, Heath zones, 2130–3600 m. Voucher: Napier 1992 (EA).

**Wahlenbergiakrebsiisubsp.arguta (Hook. f.) Thulin.** Habit: Herb. Habitat: Grassland, in lower alpine and heath zone, 2100–3550 m. Voucher: Thulin & Tidigs 70 (EA).

***Wahlenbergiascottii* Thulin** Habit: Herb. Habitat: Wooded grassland, 2300–2700 m. Vouchers: Thulin & Tidigs 131 (EA), Verdcourt 2427 (EA).

***Wahlenbergiasilenoides* Hochst. ex A. Rich.** Habit: Herb. Habitat: Montane grassland, 1500–2100 m. Voucher: Tweedie 3020 (EA).

**F126. Asteraceae**

66 Genera, 165 Species

***Acanthospermumglabratum* (DC.) Wild** Habit: Herb. Habitat: Grassland, 1300–2500 m. Voucher: SAJIT Z0007 (EA).

***Acmellacaulirhiza* Delile** Habit: Herb. Herb. Habitat: Grassland, 600–2600 m. Vouchers: FOKP 1230 & 11427 (EA) Mbuni 163 (EA).

***Ageratinaadenophora* (Spreng.) R.M. King & H. Rob.** Habit: Shrub. Habitat: Swampy sites, riverine, 1960–2300 m. Vouchers: SAJIT 005063 & 006809 (EA, HIB), FORK 1806 & 10909 (EA, HIB).

***Ageratumconyzoides* (L.) L.** Habit: Herb. Habitat: Disturbed ground, 30–2500 m. Voucher: FOKP 11495 (EA), Mbuni 231 (EA).

***Anthemistigrensis* J. Gay ex A. Rich.** Habit: Herb. Habitat: Moorland zone, 2500–3500 m. Vouchers: Mabberley 214, Thulin 203 (EA).

***Artemisiaafra* Jacq. ex Willd**. Habit: Shrub. Habitat: Dry shrubby montane grassland, 2000–3550 m. Vouchers: SAJIT 005114 (EA, HIB), Mbuni 725 (EA, HIB), Mabberley & McCall 241 (EA), Thulin 252 (EA).

***Aspiliakotschyi* (Sch. Bip. ex Hochst.) Oliv.** Habit: Herb. Habitat: Grassland, waste land, 10–1900 m. Voucher: Carter & Stanard 52 (EA).

***Aspiliapluriseta* Schweinf. ex Schweinf.** Habit: Herb. Habitat: Bushed grassland, 1050–2400 m. Vouchers: FOKP 11492 (EA), Mbuni 228 (EA), Symes 98 (EA).

***Athrixiarosmarinifolia* (Sch. Bip. ex Walp.) Oliv. & Hiern.** Habit: Herb. Habitat: Highland shrubland, 2600–3400 m. Vouchers: SAJIT 005119 (EA, HIB), Tweedie 3904 (EA).

***Baccharoidesdumicola* (S. Moore) “Isawumi, El-Ghazaly & B. Nord.**” Habit: Herb. Habitat: Woodland, grassland edge, 1200–2300 m. Voucher: Webster 8898 (EA).

***Baccharoidesadoensis* (Sch. Bip. ex Walp.) H. Rob.** Habit: Shrub. Habitat: Bushland, 125–2100 m. Voucher: FOKP 11386 (EA, HIB).

***Berkheyaspekeana* Oliv.** Habit: Herb. Habitat: Wooded grassland, 1800–3100 m. Vouchers: SAJIT 005124 (EA, HIB), FOKP 1905 (EA, HIB), Symes 157 (EA).

***Bidensbuchneri* (Klatt) Sherff** Habit: Herb. Habitat: Wooded grassland, 1450–1500 m. Vouchers: Napier 1924 (EA), Agnew et al. 312 (EA).

***Bidenselgonensis* (Sherff) Agnew** Habit: Herb. Habitat: Erica bushland, heath zone, 1900–3550 m. Vouchers: FOKP 11280 (EA, HIB), Mbuni 016 (EA), Tweedie 338 (EA).

***Bidensflagellata* (Sherff) Mesfin** Habit: Herb. Habitat: Grassland, 1600–3300 m. Voucher: Thulin & Tidigs 264 (EA).

***Bidenskilimandscharica* (O. Hoffm.) Sherff** Habit: Shrub. Habitat: Upland forest edges, 1500–2800 m. Vouchers: FOKP 11569 (EA, HIB), Mbuni 306 (EA), Tweedie 4214 (EA).

***Bidenslineariloba* Oliv.** Habit: Herb. Habitat: Bushland, wooded land, 500–1800 m. Voucher: Tweedie 369 (EA).

***Bidenspilosa* L.** Habit: Herb. Habitat: Highland areas, 400–2500 m. Vouchers: FOKP 11434 (EA), Mbuni 170 (EA).

***Blumeaaxillaris* (Lam.) DC.** Habit: Herb. Habitat: Disturbed areas, 1500–2700 m. Voucher: FOKP 11366 (EA), Mbuni 102 (EA).

***Bothrioclinefusca* (S. Moore) M.G. Gilbert** Habit: Shrub. Habitat: Upper forest zones, 2000–3550 m. Voucher: FOKP 11377 (EA, HIB).

***Bothrioclinelongipes* (Oliv. & Hiern) N.E. Br.** Habit: Shrub. Habitat: Forest margin, 1500–2800 m. Vouchers: FOKP 11519 (EA, HIB), Mbuni 255 (EA).

***Bothrioclinemonticola* (M. Taylor) Wech.** Habit: Shrub. Habitat: Forest margin, about 2800 m. Voucher: Tweedie 4200 (EA).

***Bothrioclinetrifoliata* (De Wild. & Muschl.) Wild & G.V. Pope** Habit: Shrub. Habitat: Montane rain forest, 1950–2500 m. Voucher: Thulin & Tidigs 209 (EA).

***Carduuschamaecephalus* (Vatke) Oliv. & Hiern** Habit: Herb. Habitat: Grassland, alpine, subalpine, 2400–3550 m. Voucher: Gillett 18422 (EA).

***Carduuskeniensis* R.E. Fr.** Habit: Herb. Habitat: Grassland, 2950–3550 m. Vouchers: Mbuni 098 (EA), Mabberley & McCall 212 (EA).

***Carduusnyassanus* (S. Moore) R.E. Fr.** Habit: Herb. Habitat: Montane forest, 2700–3550 m. SAJIT 004773 (EA, HIB), Vouchers: FOKP 11363 (EA, HIB), Mbuni 099 (EA), Symes 402 (EA).

***Carduusschimperi* Sch. Bip.** Habit: Herb. Habitat: Grassland & Moorland, 2550–3550 m. Voucher: Gillett 18422 (EA).

***Centaureapraecox* Oliv. & Hierm** Habit: Herb. Habitat: Wooded grassland, 1600–2300 m. Vouchers: Symes 523 (EA), Tweedie 1519 (EA), Thairu 143 (EA), Webster 8856 (EA).

***Cinerariagrandiflora* Vatke** Habit: Herb. Habitat: Wooded grassland, 1800–3500 m. Voucher: Thulin 72 (EA).

***Cinerariadeltoidea* Sond.** Habit: Herb. Habitat: Roadsides, forest edges cliffs, 1890–3550 m. Vouchers: FOKP 1131 & 11339 (EA, HIB), Mbuni 075 (EA), Thulin & Mhoro 72 (EA).

***Cirsiumvulgare* (Savi) Ten.** Habit: Herb. Habitat: Arable land, 1790–2400 m. Vouchers: FOKP 1369 & 11362 (EA, HIB), Mbuni 098 & 105 (EA).

***Conyzaaegyptiaca* (L.) Dryand. ex Aiton** Habit: Herb. Habitat: Disturbed places, 300–2000 m. Voucher: Symes 64 (EA).

***Conyzaagnewii* Mesfin** Habit: Herb. Habitat: Wooded grassland around 1850 m. Voucher: Napier 1931 (EA).

***Conyzanewii* Oliv. & Hiern.** Habit: Shrub. Habitat: Montane forest, 1600–2500 m. Vouchers: FOKP 11321 (EA, HIB) Mbuni, 057 (EA).

***Conyzapyrrhopappa* Sch. Bip. ex A. Rich.** Habit: Shrub. Habitat: Upland bushland, 1500–2200 m. Vouchers: SAJIT 006854 (EA, HIB), FOKP 11442 (EA, HIB), Mbuni 178 (EA).

***Conyzasteudelii* Sch. Bip. ex A. Rich.** Habit: Herb. Habitat: Medium altitude, 1500–3500 m. Vouchers: FOKP 11346 (EA, HIB), Mbuni 082 (EA).

***Conyzasubscaposa* O. Hoffm.** Habit: Herb. Habitat: Alpine and subalpine grassland, 3000–3500 m. Voucher: Symes 618 (EA).

***Conyzavernonioides* (Sch. Bip. ex A. Rich.) Wild** Habit: Shrub. Habitat: Moorland and bamboo, 3000–3550 m. Voucher: Tweedie 3901 (EA).

***Cotulaabyssinica* Sch. Bip. ex A. Rich.** Habit: Herb. Habitat: Alpine, upland grassland, 2700–3500 m. Vouchers: Bogdan 496 (EA), Symes 643 (EA), and Mabberley 216 (EA).

***Crassocephalumbojeri* (DC.) Robyns** Habit: Herb. Habitat: Montane forest, 1500–3200 m. Voucher: Thulin 159 (EA).

***Crassocephalummontuosum* (S. Moore) Milne–Redh**. Habit: Herb. Habitat: Montane forest, 1500–3260 m. Vouchers: FOKP 1724 (EA, HIB), Chetham 1 (EA), Thulin & Tidigs 266 (EA).

***Crassocephalum×picridifolium* (DC.) S. Moore** Habit: Herb. Habitat: Swamps and river edges, 1300–2500 m. Vouchers: SAJIT 004806 (EA, HIB), FOKP 11503 (EA, HIB), Mbuni 239 (EA), Symes 269 (EA).

**Crassocephalumrubensvar.sarcobasis (Bojer ex DC.) C. Jeffrey & Beentje** Habit: Herb. Habitat: Disturbed, cultivated, higher altitude, 1500–2800 m. Vouchers: Verdcourt 746 (EA), Thulin 188 (EA), Symes 205 (EA).

***Crassocephalumvitellinum* (Benth.) S. Moore** Habit: Herb. Habitat: Upland forest and woodland, 1500–2800 m. Vouchers: FOKP 981 (EA, HIB), Thulin 158 (EA).

***Crepisrueppellii* Sch. Bip.** Habit: Herb. Habitat: Highland grassland, 1600–3200 m. Vouchers: FOKP 1221 (EA, HIB), Townsend 2368 (EA).

***Dendroseneciocheranganiensis* (Cotton & Blakelock) E.B. Knox** Habit: Shrub. Habitat: Upper Afromontane evergreen, 3000–3550 m. Vouchers: Dale 3392 (EA), Mabberley 30 (EA).

**Dendroseneciocheranganiensissubsp.cheranganiensis (Cottton & Blakeclock) E.B. Knox** Habit: Shrub. Habitat: Upper Afromontane evergreen, 3000–3550 m. Vouchers: Merbbery 512 (EA), Knox 719 (EA), Beentje 3058 (EA).

**Dendroseneciocheranganiensissubsp.dalei (Cotton & Blakelock) E.B. Knox** Habit: Shrub. Habitat: Upper Afromontane zone, 3000–3550 m. Voucher: Mabberley & McCall 209 (EA).

***Dichrocephalaintegrifolia* (L. f.) Kuntze** Habit: Herb. Habitat: Disturbed wet habitat, 1700–3550 m. Vouchers: FOKP 111406 (EA, HIB), Mbuni 142 (EA, HIB).

***Dicomatomentosa* Cass**. Habit: Herb. Habitat: Dry bushland, 480–1500 m. Voucher: Carter & Stanard 13 (EA).

***Emiliaabyssinica* (Sch. Bip. ex A. Rich.) C. Jeffrey** Habit: Herb. Habitat: Cultivated land, 800–1900 m. Voucher: Tweedie 1922 (EA).

***Emiliacoccinea* (Sims) G. Don** Habit: Herb. Habitat: Roadside, wasteland, 0–2000 m. Voucher: Carter & Stannard 15 (EA).

***Emiliadebilis* S. Moore** Habit: Herb. Habitat: Disturbed marshes, 1600–2200 m. Voucher: Symes 751 (EA)

***Echinopsaberdaricus* R.E. Fr.** Habit: Herb. Habitat: Subalpine grassland, 2900–3300 m. Voucher: Thulin 143 (EA).

***Echinopsangustilobus* S. Moore** Habit: Herb. Habitat: Higher altitudes, grassland, 2000–2800 m. Vouchers: FOKP 1139 (EA, HIB), Bogdan 5008 (EA), Townsend 2376 (EA).

***Echinopsamplexicaulis* Oliv.** Habit: Herb. Habitat: Wooded grassland, 1300–2700 m. Voucher: Thairu 1, Napper 1501 (EA).

***Echinopshispidus* Fresen.** Habit: Herb. Habitat: Upland grassland, 1500–2200 m. Voucher: Symes 403 (EA).

***Echinopshoehnelii* Schweinf.** Habit: Herb. Habitat: High altitude woodland & heartland, 2200–3500 m. Vouchers: FOKP 11381 (EA), Mbuni 116 & 118 (EA).

***Echinopslanatus* C. Jeffrey & Mesfin** Habit: Herb. Habitat: Montane grassland, 2400–3600 m. Voucher: Tweedie 4183 (EA).

***Erigeronbonariensis* L.** Habit: Herb. Habitat: Disturbed soil, 400–2600 m. Vouchers: FOKP 11338 & 11724 (EA, HIB), Mbuni 074 & 724 (EA), Cronquist 2396 (EA).

***Euryopsbrownei* S. Moore** Habit: Shrub. Habitat: Upper forest level and lower heath zone, 2300–3550 m. Vouchers: SAJIT 004794, Tweedie 391 (EA), Lucas 165 (EA), Wamukoya 129 (EA).

***Euryopschrysanthemoides* (DC.) B. Nord.** Habit: Shrub. Habitat: Heath zone, 2500–3500 m. Vouchers: FOKP 11578 & 11624 (EA, HIB), Mbuni 315 & 624 (EA).

***Ethuliavernonioides* (Schweinf.) M.G. Gilbert** Habit: Shrub. Habitat: Grassland, 1650–2750 m. Vouchers: SAJIT 006815 (EA, HIB), FOKP 989, 1803 & 11550 (EA, HIB), Mbuni 287 (EA), Webster 888 (EA).

***Eupatoriumadenophorum* Spreng.** Habit: Herb. Habitat: Riverine, 1750–2500 m. Voucher: Symes 282 (EA).

***Eupatoriumafricanum* Oliv. & Hieron.** Habit: Herb. Habitat: Riverine, 1700–2500 m. Voucher: Symes 31 (EA).

***Feliciaabyssinica* Sch. Bip. ex A. Rich.** Habit: Herb. Habitat: Grassland, 1300–2500 m. Voucher: Symes 527 (EA).

***Galinsogaquadriradiata* Ruiz & Pav.** Habit: Herb. Habitat: Wet highland, 1800–2500 m. Vouchers: FOKP 11426 (EA, HIB), Mbuni 162 (EA).

***Galinsogaparviflora* Cav.** Habit: Herb. Habitat: Wet highland, 300–2300 m. Voucher: Jeffery 272 (EA).

***Gerberahirsuta* (Forssk.) Less.** Habit: Herb. Habitat: Highland grassland, 1500–3000 m. Voucher: Mabberley 208 (EA).

***Gerberaviridifolia* (DC.) Sch. Bip.** Habit: Herb. Habitat: Wooded grassland, 1600–2500 m. Voucher: Symes 52 (EA).

***Gerberapiloselloides* (L.) Cass.** Habit: Herb. Habitat: Upland and highland grassland, 1500–3400 m. Voucher: SAJIT 005106 (EA, HIB).

***Gnaphaliumunionis* Sch. Bip. ex Oliv. & Hiern** Habit: Herb. Habitat: Grassland, 1600–3200 m. Vouchers: FOKP 1794 (EA, HIB), Townsend 238 (EA).

***Gnaphaliumdeclinatum* L. f.** Habit: Herb. Habitat: Grassland, 1600–3000 m. Voucher: Trelawny 4384 (EA).

***Gnaphaliumluteoalbum* L.** Habit: Herb. Habitat: Highland grassland, 300–3050 m. Vouchers: Symes 74 (EA), Thulin & Tidigs 115 (EA).

***Guizotiascabra* (Vis.) Chiov.** Habit: Herb. Habitat: Upland grassland, 1300–3200 m. Vouchers: Thulin 46 (EA), Lind 5098 (EA).

***Guizotiajacksonii* (S. Moore) J. Baagøe** Habit: Herb. Habitat: Bamboo, heath zone, 2400–3500 m. Voucher: Thulin & Tidigs 68 (EA).

***Gutenbergiarueppellii* Sch. Bip.** Habit: Herb. Habitat: Rocky grassland, 900–2200 m. Voucher: Bogdan 3853 (EA).

***Haplocarpharueppellii* (Sch. Bip.) K. Lewin**. Habit: Herb. Habitat: Alpine zone, 2600–3550 m. Voucher: SAJIT 005123 (EA, HIB).

***Helichrysumargyranthum* O. Hoffm.** Habit: Shrub. Habitat: Upper forest edge, streamside, heath zone, 2100–3550 m. Vouchers: FOKP 996, 11264, 11374 & 11340 (EA, HIB), Mbuni 020 (EA).

***Helichrysumbrownei* S. Moore** Habit: Shrub. Habitat: Heath zone, 3000–3550 m. Voucher: SAJIT 005111 (EA, HIB).

***Helichrysumcymosum* (L.) D. Don** Habit: Shrub. Habitat: Wooded land, 1600–2900 m. Voucher: Symes 624 (EA).

***Helichrysumellipticifolium* Moeser** Habit: Shrub. Habitat: Alpine grassland, 2650–3550 m. Voucher: Knox 3390 (EA).

***Helichrysumfoetidum* (L.) Cass.** Habit: Herb. Habitat: Upland forest, forest margin, 2400–3000 m. Vouchers: FOKP 11520 (EA, HIB), Mbuni 256 & 723 (EA), Mabberley 583 (EA), Symes 99 (EA).

***Helichrysumformosissimum* Sch. Bip.** Habit: Shrub. Herb. Habitat: Alpine zone, 2300–3500 m. Voucher: Bogdan 5000 (EA).

**Helichrysumformosissimumvar.guilelmii (Engl.) Mesfin** Habit: Herb. Habitat: Heath zone, moorland, upper bamboo zone, 1800–2500 m. Voucher: Mabberley 571 (EA).

***Helichrysumforskahlii* (J.F. Gmel.) Hilliard & B.L. Burtt** Habit: Herb. Habitat: Rocky grassland, 1200–3500 m. Vouchers: SAJIT 005104 & 006805 (EA, HIB), FOKP 10905 & 11538 (EA, HIB), Mbuni 274 (EA), Knox 3352 (EA), Symes 99 (EA).

***Helichrysumgerberifolium* Sch. Bip. ex A. Rich.** Habit: Herb. Habitat: Wooded grassland, 2600–3500 m. Voucher: Symes 25 (EA).

***Helichrysumglobosum* Sch. Bip.** Habit: Herb. Habitat: Upland grassland, 1800–3300 m. Vouchers: FOKP 11478 (EA, HIB), Mbuni 214 (EA).

***Helichrysum gloria–dei* Chiov**. Habit: Shrub. Habitat: Alpine zone, 3300–3550 m. Voucher: SAJIT 005108 (EA, HIB).

***Helichrysumkilimanjari* Oliv.** Habit: Herb. Habitat: Lower alpine zone, 2800–3500 m. Vouchers: Mabberley & McCall 218 (EA), Knox 3863 (EA).

***Helichrysummaranguense* O. Hoffm.** Habit: Shrub. Habitat: Montane rain forest, 2250–3500 m. Vouchers: SAJIT 006922 (EA, HIB), FOKP 11022 (EA, HIB).

***Helichrysummeyeri-johannis* Engl**. Habit: Herb. Habitat: Alpine grassland, 2900–3550 m. Voucher: Dale 3418 (EA).

***Helichrysumnandense* S. Moore** Habit: Shrub. Habitat: Grassland, 2250–2900 m. Vouchers: Knox 3447 (EA), Blake 3080 (EA).

***Helichrysumodoratissimum* (L.) Sweet** Habit: Shrub. Habitat: Dry areas, 1000–3500 m. Vouchers: FOKP 11537 (EA, HIB), Mbuni 273 (EA), Thulin & Tidigs 254 (EA).

**Helichrysumpanduratumvar.panduratum O. Hofffm.** Habit: Shrub. Habitat: Grassland, 1600–2900 m. Voucher: Symes 396 (EA).

***Helichrysumsetosum* Harv.** Habit: Herb. Habitat: Montane forest, 1900–2750 m. Voucher: FOKP 944 (EA, HIB).

***Helichrysumschimperi* (Sch. Bip. ex A. Rich.) Moeser** Habit: Shrub. Habitat: Dry forest 1600–3400 m. Vouchers: FOKP 11340 (EA, HIB), Mbuni 076 (EA).

***Helichrysumstenopterum* DC.** Habit: Herb. Habitat: Upper forest edge, 2660–3500 m. Vouchers: FOKP 11285 (EA, HIB), Mbuni 021 (EA), Symes 206 (EA).

***Hirpiciumdiffusum* (O. Hoffm.) Roessler** Habit: Herb. Habitat: Disturbed grassland, 500–2200 m. Vouchers: FOKP 11690 & 14476 (EA), Mbuni 212 & 690 (EA).

***Inulaglomerata* Oliv. & Hiern** Habit: Herb. Habitat: Wooded grassland, 1500–2900 m. Vouchers: Freidberg & Kaplan 56 (EA), FOKP 11328 (EA, HIB).

***Inulamannii* (Hook. f.) Oliv. & Hiern** Habit: Herb. Habitat: Wet montane forest, 1700–2750 m. Voucher: Tweedie 4184 (EA).

***Kleiniascottioides* C. Jeffrey.** Habit: Herb. Habitat: Bushland, wooded grassland, 900–1900 m. Voucher: Meyerhoff 112 (EA).

***Lactucacapensis* Thunb.** Habit: Herb. Habitat: Disturbed ground, montane forest, 1900–3200 m. Voucher: Symes 43 (EA).

***Lactucaglandulifera* Hook. f**. Habit: Herb. Habitat: Montane rain forest, 1900–3290 m. Vouchers: FOKP 1040 (EA, HIB), FOKP 938 (EA).

***Lactucainermis* Forssk.** Habit: Herb. Habitat: Medium altitude, 500–3300 m. Vouchers: FOKP 11348 (EA, HIB), Mbuni 084 (EA).

***Laggerabrevipes* Oliv. & Hiern** Habit: Herb. Habitat: Upland grassland, 1050–2400 m. Vouchers: SAJIT 006881 (EA, HIB), Mbuni 240A (EA).

***Laggeracrispata* (Vahl) Hepper & J.R.I. Wood** Habit: Shrub. Habitat: Grassland and forest margin, 150–2400 m. Vouchers: SAJIT 006801 (EA, HIB), FOKP 939, 1011, 10901 & 11505 (EA, HIB), Mbuni 241 (EA).

***Laggeraelatior* R.E. Fr.** Habit: Herb. Habitat: Wet Montane forest, 100–3300 m. Vouchers: FOKP 11372 (EA, HIB), Mbuni 065 (EA), Mabberley 483 (EA).

***Laggerapterodonta* (DC.) Sch. Bip. ex Oliv.** Habit: Herb. Habitat: Upland grassland, 1050–2400 m. Voucher: FOKP 1011 (EA, HIB).

***Melantherapungens* Oliv. & Hiern** Habit: Herb. Habitat: Wooded grassland, 1650–2300 m. Voucher: Napier 1929 (EA, HIB).

***Melantherascandens* (Schumach. & Thonn.) Roberty** Habit: Shrub. Habitat: Upland forest, 1300–2200 m. Vouchers: FOKP 11755 (EA, HIB), Mbuni 755 (EA).

***Micractisbojeri* DC.** Habit: Herb. Habitat: Disturbed streamside, 1700–2300 m. Vouchers: FOKP 11409 (EA, HIB), Mbuni 145 (EA).

***Microglossadensiflora* Hook. f.** Habit: Shrub. Habitat: Bushland, 1700–3200 m. Vouchers: SAJIT 006797 (EA, HIB), FOKP 1219 & 10897 (EA, HIB), Agnew et al. 10462 (EA).

***Mikaniachenopodifolia* Willd.** Habit: Climber. Habitat: Riverine forest, 0–2000 m. Vouchers: SAJIT 006921 (EA, HIB), FOKP 946 & 11021 (EA, HIB).

***Mikaniopsisbambuseti* (R.E. Fr.) C. Jeffrey** Habit: Climber. Habitat: Dry upland forest, bamboo, 2100–3000 m. Vouchers: SAJIT 006929 (EA, HIB), Townsend 2393 (EA).

***Nidorellaspartioides* (O. Hoffm.) Cronquist** Habit: Herb. Habitat: Wooded grassland, 1675–2150 m. Voucher: Napier 1920 (EA).

***Notoniaabyssinica* A. Rich.** Habit: Herb. Habitat: Bushland, 1600–2800 m. Vouchers: FOKP 11559 (EA, HIB) & Mbuni 296 (EA).

***Pentasparvifolia* Hiern** Habit: Shrub. Habitat: Bushland and Wooded grassland, 90–2500 m. Vouchers: FOKP 11467 & 11670 (EA, HIB) & Mbuni 203 & 670 (EA).

**Pseudognaphaliumluteoalbumsubsp.affine (D. Don) Hilliard & Burtt** Habit: Herb. Habitat: Highland grassland, 300–3050 m. Vouchers: FOKP 11303 (EA, HIB), Mbuni 039 (EA), Thulin & Tidigs 115 (EA).

***Psiadiapunctulata* (DC.) Vatke** Habit: Shrub. Habitat: Grassland, bushland, forest edge, 1000–2500 m. Vouchers: FOKP 11465 (EA, HIB), Mbuni 201 & 748 (EA).

***Seneciohedbergii* C. Jeffrey** Habit: Shrub. Habitat: Rocky open grassland, Heath Vegetation, 3300–3550 m. Voucher: Thulin & Tidigs 217 (EA).

***Seneciomaranguensis* O. Hoffm**. Habit: Shrub. Habitat: Upper forest edge, 2700–2900 m. Voucher: SAJIT 005117 (EA, HIB).

***Seneciomoorei* R.E. Fr.** Habit: Shrub. Habitat: Grassland, 1800–3500 m. Voucher: Townsend 2381 (EA), Thulin 167 (EA).

***Senecioplantagineoides* C. Jeffrey** Habit: Herb. Habitat: Grassland, 1700–2000 m. Voucher: Powles 12 (EA).

***Seneciopseudosubsessilis* C. Jeffrey** Habit: Herb. Habitat: Woodland edge, riverine, 1800–3000 m. Vouchers: Tweedie 3379 (EA), Tweedie 2169 (EA).

***Seneciopurtschelleri* Engl.** Habit: Herb. Habitat: Alpine zone, 3200–3550 m. Voucher: Beentje 3378 (EA).

***Seneciopseudosubsessilis* C. Jeffrey** Habit: Shrub. Habitat: Woodland edge, riverine, 1800–3000 m. Voucher: Thulin & Tidigs 217 (EA).

***Senecioschweinfurthii* O. Hoffm.** Habit: Herb. Habitat: Montane, alpine grassland, 2300–3500 m. Vouchers: Tweedie 3763(EA), Thulin & Tidigs 164 (EA), Part II Botany 5157(EA), Symes 640 (EA).

***Seneciosubsessilis* Oliv. & Hiern** Habit: Herb. Habitat: Montane rain forest, 2400–3120 m. Voucher: Thulin 187 (EA).

***Seneciosyringifolius* O. Hoffm.** Habit: Climber. Habitat: Montane rain forest, 1500–3300 m. Voucher: Townsend 2392 (EA).

***Schkuhriapinnata* (Lam.) Kuntze ex Thell.** Habit: Herb. Habitat: Cultivated land, 1000–2220 m. Vouchers: FOKP 11484 (EA), Mbuni 220 (EA).

***Solaneciomannii* (Hook. f.) C. Jeffrey** Habit: Tree. Habitat: Montane forest, 1400–2700 m. Vouchers: FOKP 1110, 1261, 11279 & 11457 (EA, HIB), Mbuni 193 (EA).

***Sonchusafromontanus* R.E. Fr.** Habit: Herb. Habitat: Wet highland grassland, 2200–3700 m. Voucher: Thulin & Tidigs 226 (EA).

***Sonchusschweinfurthii* Oliv. & Hiern** Habit: Herb. Habitat: Montane forest, 1700–2800 m. Voucher: Thulin & Tidigs 69 (EA).

***Spilanthesmauritiana* (A. Rich. ex Pers.) DC.** Habit: Herb. Habitat: Swamp areas, 1500–2000 m. Voucher: Hedberg 79 (EA).

***Sphaeranthussuaveolens* (Forssk.) DC.** Habit: Herb. Habitat: Dry stream bed, 1200–2500 m. Vouchers: FOKP 1729, 11324 & 11653 (EA, HIB), Mbuni 060, 653 (EA).

***Stoebekilimandscharica* O. Hoffm.** Habit: Shrub. Habitat: Heath zone and moorland, 2700–3350 m. Vouchers: FOKP 11317 (EA, HIB), Mbuni 053 (EA), Mwangangi & Kariuki 372 (EA), Thulin & Tidigs 250 (EA).

***Stomatanthesafricanus* (Oliv. & Hiern) R.M. King & H. Rob.** Habit: Herb. Habitat: Wooded grassland, 2000–3000 m. Voucher: Symes 282 (EA).

***Tarchonanthuscamphoratus* L.** Habit: Shrub. Habitat: Bushland, 1500–2300 m. Voucher: FOKP 11297 (EA, HIB).

***Tolpiscapensis* (L.) Sch. Bip.** Habit: Herb. Habitat: Wet grassland, 2000–3100 m. Vouchers: FOKP 11301 (EA, HIB), Mbuni 037 (EA), Townsend 2368b (EA), Hepper & Field 5031 (EA).

***Tridaxprocumbens* (L.) L**. Habit: Herb. Habitat: Roadside, below 2300 m. Voucher: SAJIT Z0038 (EA, HIB).

***Vernoniaamygdalina* Delile** Habit: Shrub. Habitat: Disturbed cultivated area, 200–2300 m. Voucher: FOKP 10934 (EA, HIB).

***Vernoniaauriculata* Griseb.** Habit: Shrub. Habitat: Woodland, bushland, 1600–2850 m. Voucher: FOKP 959 (EA, HIB).

***Vernoniaauriculifera* Hiern** Habit: Shrub. Habitat: Woodland, bushland, 1600–2650 m. Vouchers: SAJIT 006834 (EA, HIB), FOKP 11401 & 11718 (EA, HIB), Mbuni 137 & 718 (EA), Symes 268 (EA).

***Vernoniaholstii* O. Hoffm.** Habit: Shrub. Habitat: Dry forest, 1000–2050 m. Voucher: FOKP 11454 (EA, HIB), Mbuni 190 & 718 (EA).

**Vernoniagalamensissubsp.afromontana (R.E. Fr.) M.G. Gilbert** Habit: Shrub. Habitat: Woodland edge, 800–2200 m. Vouchers: SAJIT 006896 (EA, HIB), FOKP 11331 & 11400 (EA, HIB), Mbuni 067 & 136 (EA), Napier 1957 (EA).

***Vernoniahymenolepis* A. Rich.** Habit: Herb. Habitat: Bushed grassland, 1600–2850 m. Vouchers: FOKP 11287 & 11402 (EA, HIB), Mbuni 023, 713 (EA), Thulin & Tidigs 125 (EA).

***Vernoniakaraguensis* Oliv. & Hiern** Habit: Herb. Habitat: Wooded grassland, 1200–2500 m. Voucher: Webster 8894 (EA).

***Vernonialasiopus* O. Hoffm.** Habit: Herb. Habitat: Abandoned cultivation, 1100–2800 m. Voucher: Symes 261 (EA).

***Vernoniasmithiana* Less.** Habit: Herb. Habitat: Wooded grassland, 1350–2700 m. Vouchers: Napier 1934 (EA), Symes 27 (EA).

***Vernoniasyringifolia* O. Hoffm.** Habit: Shrub. Habitat: Montane rain forest, 2000–3000 m. Vouchers: SAJIT 006800 & 006818 (EA, HIB), Webster 8897 (EA).

***Vernoniaturbinata* Oliv. & Hiern ex Oliv.** Habit: Herb. Habitat: Wooded grassland, 1400–2400 m. Voucher: Verdcourt 745 (EA).

***Vernoniaurticifolia* A. Rich.** Habit: Shrub. Habitat: Forest margin and bamboo edge, 1400–2800 m. Vouchers: SAJIT Z0084 (EA, HIB), FOKP 11358 (EA, HIB), Mbuni 094 (EA).

***Vernoniawestermanii* Ekman & Dusén ex Malme** Habit: Herb. Habitat: Wooded grassland, 1400–2400 m. Voucher: FOKP 1064 (EA, HIB).

***Xanthiumpungens* Wallr.** Habit: Herb. Habitat: River bed, 1600–1800 m, Voucher: FOKP 11302 (EA, HIB).

**F127. Caprifoliaceae**

3 Genera, 3 Species

***Cephalariapungens* Szabó** Habit: Herb. Habitat: Montane and subalpine grassland, 2400–3000 m. Vouchers: FOKP 11316 (EA, HIB), Irwin 136 (EA).

***Scabiosacolumbaria* L.** Habit: Herb. Habitat: Heathland and upper land forest, 2200–3550 m. Vouchers: FOKP 11314 (EA, HIB), Webster 8843 (EA).

***Valerianavolkensii* Engl.** Habit: Herb. Habitat: streamside and marshes, 3000–3500 m. Voucher: Mabberley McCall 231 (EA).

**F128. Pittosporaceae**

1 Genus, 3 Species

***Pittosporummannii* Hook. f.** Habit: Shrub. Habitat: Dry evergreen forest, 2000–2750 m. Vouchers: SAJIT Z0026 (EA, HIB), FOKP 11570 (EA, HIB), Mbuni 307 & 651 (EA).

***Pittosporumlanatum* Hutch. & E.A. Bruce** Habit: Tree. Habitat: Dry evergreen forest, 2100–2850 m. Vouchers: FOKP 11391 (EA, HIB), Dale 672 (EA).

***Pittosporumviridiflorum* Sims.** Habit: Tree. Habitat: Wooded grassland, 1300–2500 m. Voucher: SAJIT 007050 (EA, HIB).

**F129. Araliaceae**

4 Genera, 8 Species

***Cussoniaarborea* Hochst. ex A. Rich.** Habit: Tree. Habitat: Wooded grassland, 1100–2400 m. Voucher: Napier 1980 (EA).

***Cussoniaspicata* Thunb.** Habit: Tree. Habitat: Riverine, grassland forest, 1450–2500 m. Voucher: FOKP 1026 (EA, HIB).

***Hydrocotylemannii* Hook. f.** Habit: Herb. Habitat: Forest floor, bamboo forest, 600–3370 m. Voucher: Mabberley & McCall 301 (EA).

***Hydrocotyleranunculoides* L. f.** Habit: Herb. Habitat: Common in ponds and marshes, 700–2400 m. Voucher: Barnley via Tweedie 2584 (EA).

***Polysciasfulva* (Hiern) Harms** Habit: Tree. Habitat: Wet highland forest, 1400–2310 m. Vouchers: FOKP 1072 (EA, HIB), SAJIT Z0034 (EA, HIB).

***Polysciaskikuyuensis* Summerh.** Habit: Tree. Habitat: Wet upland forest, 1750–2750 m. Voucher: FOKP 1722 (EA, HIB).

***Scheffleraabyssinica* (Hochst. ex A. Rich.)** Harms. Habit: Tree. Habitat: Wet upland forest, 1840–2770 m. Vouchers: SAJIT 004790 & 005092 (EA, HIB).

***Scheffleravolkensii* (Harms) Harms.** Habit: Tree. Habitat: Wet or dry upland forest, 1700–2250 m. Vouchers: SAJIT 004785 (EA, HIB), FOKP 1007, 1228, 1892 & 11388 (EA, HIB), Friis et al. 2530 (EA).

**F130. Apiaceae**

14 Genera, 22 Species

***Agrocharismelanantha* Hochst.** Habit: Herb. Habitat: High altitude grassland, 2300–2700 m. Voucher: Thulin & Tidings 126 (EA).

***Agrocharispedunculata* (Baker f.) Heywood & Jury.** Habit: Herb. Habitat: Wooded grassland, 1860–2100 m. Voucher: Napier 1922 (EA).

***Alepideapeduncularis* Steud. ex A. Rich.** Habit: Herb. Habitat: Montane grassland, 1530–3550 m. Vouchers: SAJIT 004810 & 005105 (EA, HIB), FOKP 11315 (EA, HIB), Mabberley & McCall 113 (EA), Napper 1503 (EA).

***Apiumleptophyllum* (Pers.) F. Muell. ex Benth.** Habit: Herb. Habitat: Disturbed areas, 1550–1660 m. Vouchers: FOKP 1299 & 11300 (EA, HIB), Mbuni 036 (EA).

***Anthriscussylvestris* (L.) Hoffm.** Habit: Herb. Habitat: Bamboo zone, 2100–3550 m. Voucher: Mabberley McCall 200 (EA).

***Caucalisincognita* C. Norman** Habit: Herb. Habitat: Grassland, 1530–2550m. Voucher: Symes 514 (EA).

***Caucalispedunculata* Baker f.** Habit: Herb. Habitat: Disturbed areas, 1550–2160 m. Voucher: Symes 524 (EA).

***Cryptotaeniaafricana* (Hook. f.) Drude** Habit: Herb. Habitat: Montane forest, 1600–3000 m. Vouchers: FOKP 1741 (EA, HIB), Irwin 3777 (EA), Mabberley & McCall 35 (EA), Irving 377 (EA).

***Diplolophiumafricanum* Turcz.** Habit: Herb. Habitat: Wooded grassland, 1350–2500 m. Voucher: Kokwaro 2535 (EA).

***Haplosciadiumabyssinicum* Hochst.** Habit: Herb. Habitat: Disturbed areas, 2150–4600 m. Voucher: Thulin & Tidigs 152 (EA).

**Heteromorphaarborescensvar.abyssinica (Hochst. ex A. Rich.) H. Wolff** Habit: Herb. Habitat: Wooded grassland, 1300–2600 m. Voucher: Thulin & Tidigs 152 (EA).

***Heteromorphatrifoliata* (H.L. Wendl.) Eckl. & Zeyh.** Habit: Habitat: Wooded grassland, 1250–2500 m. Vouchers: Mbuni 304 (EA, HIB), FOKP 11567 (EA, HIB).

***Oenantheprocumbens* (H. Wolff) Norman** Habit: Herb. Habitat: Bamboo and highland forest, 1830–3200 m. Vouchers: Mabberley 574 (EA, HIB), Thulin &Tidigs 234 (EA).

***Peucedanumaculeolatum* Engl** Habit: Herb. Habitat: Montane forest, 1900–2700 m. Voucher: FOKP 11398 (EA, HIB).

***Peucedanumelgonense* H. Wolff** Habit: Herb. Habitat: Subalpine zone, streamside marshes, 1800–3500 m. Voucher: SAJIT 004797 (EA, HIB).

***Peucedanumwinkleri* H. Wolff** Habit: Herb. Habitat: Montane forest, 1800–3500 m. Voucher: Thulin &Tidigs 45 (EA).

***Peucedanumkerstenii* Engl.** Habit: Herb. Habitat: Montane forest, 1800–3500 m. Voucher: Bogdan 4995 (EA).

***Pimpinellahirtella* A. Rich.** Habit: Herb. Habitat: Upland grassland, 1800–2850 m. Vouchers: Symes 11345 (EA), Webster 8820 (EA).

***Pimpinellalindblomii* H. Wolff** Habit: Herb. Habitat: Upland grassland, 1530–2600 m. Vouchers: FOKP 11695, Mbuni 695 (EA), Lewis 5988 (EA), Symes 755 (EA).

***Pimpinellaoreophila* Hook. f.** Habit: Herb. Habitat: Upper montane forest, heath zone, 3000–3550 m. Voucher: SAJIT 005151 (EA, HIB).

***Saniculaelata* Buch.–Ham. ex D. Don** Habit: Herb. Habitat: Bamboo zone, 1500–3220 m. Vouchers: SAJIT 006902 & 006906 (EA, HIB), FOKP 1083, 11006 & 11614 (EA, HIB), Mbuni 614 (EA), Townsend 2384 (EA).

**Torilisarvensissubsp.purpurea (Guss.) Thell**. Habit: Herb. Habitat: Drier upland forest, 1560–2850 m, Bogdan 5001 (EA).
